# Pivotal role for *S*-nitrosylation of DNA methyltransferase 3B in epigenetic regulation of tumorigenesis

**DOI:** 10.1038/s41467-023-36232-6

**Published:** 2023-02-04

**Authors:** Kosaku Okuda, Kengo Nakahara, Akihiro Ito, Yuta Iijima, Ryosuke Nomura, Ashutosh Kumar, Kana Fujikawa, Kazuya Adachi, Yuki Shimada, Satoshi Fujio, Reina Yamamoto, Nobumasa Takasugi, Kunishige Onuma, Mitsuhiko Osaki, Futoshi Okada, Taichi Ukegawa, Yasuo Takeuchi, Norihisa Yasui, Atsuko Yamashita, Hiroyuki Marusawa, Yosuke Matsushita, Toyomasa Katagiri, Takahiro Shibata, Koji Uchida, Sheng-Yong Niu, Nhi B. Lang, Tomohiro Nakamura, Kam Y. J. Zhang, Stuart A. Lipton, Takashi Uehara

**Affiliations:** 1 Department of Medicinal Pharmacology, Graduate School of Medicine, Dentistry, and Pharmaceutical Sciences, Okayama University, Okayama, Japan; 2 Chemical Genomics Research Group, RIKEN Center for Sustainable Resource Science, Wako, Saitama Japan; 3 Laboratory of Cell Signaling, Tokyo University of Pharmacy and Life Sciences, Hachioji, Tokyo Japan; 4 Laboratory for Structural Bioinformatics, Center for Biosystems Dynamics Research, RIKEN, Yokohama, Kanagawa Japan; 5 Division of Experimental Pathology, Faculty of Medicine, Tottori University, Yonago, Japan; 6 Chromosome Engineering Research Center, Tottori University, Yonago, Japan; 7 Department of Synthetic and Medicinal Chemistry, Graduate School of Medicine, Dentistry, and Pharmaceutical Sciences, Okayama University, Okayama, Japan; 8 Laboratory of Structural Biology, Graduate School of Medicine, Dentistry, and Pharmaceutical Sciences, Okayama University, Okayama, Japan; 9 Department of Gastroenterology and Hepatology, Graduate School of Medicine, Kyoto University, Kyoto, Japan; 10 Division of Genome Medicine, Institute of Advanced Medical Sciences, Tokushima University, Tokushima, Tokushima, Japan; 11 Graduate School of Bioagricultural Sciences, Nagoya University, Nagoya, 464-8601 Japan; 12 Laboratory of Food Chemistry, Department of Applied Biological Chemistry, Graduate School of Agricultural and Life Sciences, The University of Tokyo, Tokyo, Japan; 13 Broad Institute of MIT and Harvard, Cambridge, MA USA; 14 Neurodegeneration New Medicines Center, and Departments of Molecular Medicine and Neuroscience, The Scripps Research Institute, La Jolla, CA USA; 15 Department of Neurosciences, University of California, San Diego, School of Medicine, La Jolla, CA USA

**Keywords:** Nitrosylation, Methylation, Nitrosylation

## Abstract

DNA methyltransferases (DNMTs) catalyze methylation at the C5 position of cytosine with *S*-adenosyl-l-methionine. Methylation regulates gene expression, serving a variety of physiological and pathophysiological roles. The chemical mechanisms regulating DNMT enzymatic activity, however, are not fully elucidated. Here, we show that protein S-nitrosylation of a cysteine residue in DNMT3B attenuates DNMT3B enzymatic activity and consequent aberrant upregulation of gene expression. These genes include Cyclin D2 (*Ccnd2*), which is required for neoplastic cell proliferation in some tumor types. In cell-based and in vivo cancer models, only DNMT3B enzymatic activity, and not DNMT1 or DNMT3A, affects *Ccnd2* expression. Using structure-based virtual screening, we discovered chemical compounds that specifically inhibit *S*-nitrosylation without directly affecting DNMT3B enzymatic activity. The lead compound, designated DBIC, inhibits *S*-nitrosylation of DNMT3B at low concentrations (IC_50_ ≤ 100 nM). Treatment with DBIC prevents nitric oxide (NO)-induced conversion of human colonic adenoma to adenocarcinoma in vitro. Additionally, in vivo treatment with DBIC strongly attenuates tumor development in a mouse model of carcinogenesis triggered by inflammation-induced generation of NO. Our results demonstrate that de novo DNA methylation mediated by DNMT3B is regulated by NO, and DBIC protects against tumor formation by preventing aberrant *S*-nitrosylation of DNMT3B.

## Abstract (web summary)

Here the authors demonstrate that de novo DNA methylation mediated by DNMT3B is regulated by nitric oxide (NO). They also isolate a unique modulator (DBIC) that inhibits S-nitrosylation of DNMT3B, which mitigates cell proliferation and tumorigenic conversion in vivo.

## Introduction

Nitric oxide (NO) and related reactive nitrogen species (RNS) regulate cellular proliferation, apoptosis, and neurotransmission, in large part via protein *S*-nitrosylation^1–5^. This process occurs from oxidative reaction between NO-related species (probably in the form of nitrosonium ion or NO^+^) and a cysteine (Cys) thiol (-SH) group (or more properly thiolate anion, S^-^); alternatively, transnitrosylation can transfer a NO group from one *S*-nitrosylated protein to another^4,5^. *S*-Nitrosylation serves to modulate protein function and localization, affecting processes ranging from enzymatic activity to ion channel permeation to protein-protein interactions^6–8^. Although numerous protein substrates for *S*-nitrosylation have been identified, little is known about the regulation of epigenetic enzymes by this process. Recently, it was demonstrated that *S*-nitrosylation of histone deacetylase 2 (HDAC2) results in neuronal chromatin remodeling and activation of genes associated with neuronal development^9^. In this case, however, *S*-nitrosylation does not affect enzymatic activity directly, but instead induces the release of HDAC2 from chromatin.

The enzyme DNA methyltransferase (DNMT) plays a central role in epigenomic regulation and consists of three highly conserved proteins in mammals: DNMT1, DNMT3A, and DNMT3B^10–18^. During DNA replication, DNMT1 interacts with hemi-methylated DNA at CpG sites and methylates cytosines on newly synthesized strands. DNMT3A and 3B function as de novo DNMTs but are not involved in genome-wide DNA methylation or maintenance like DNMT1. However, the mechanisms regulating DNMT enzyme activity remain largely unknown. Here, we show that DNMT activity is regulated by nitrosative stress resulting in *S*-nitrosylation. Moreover, we identify a chemical modulator capable of attenuating *S*-nitrosylation of DNMT3B.

## Results and discussion

### *S*-Nitrosylation of DNMT in vitro and in vivo

**Fig. 1:**
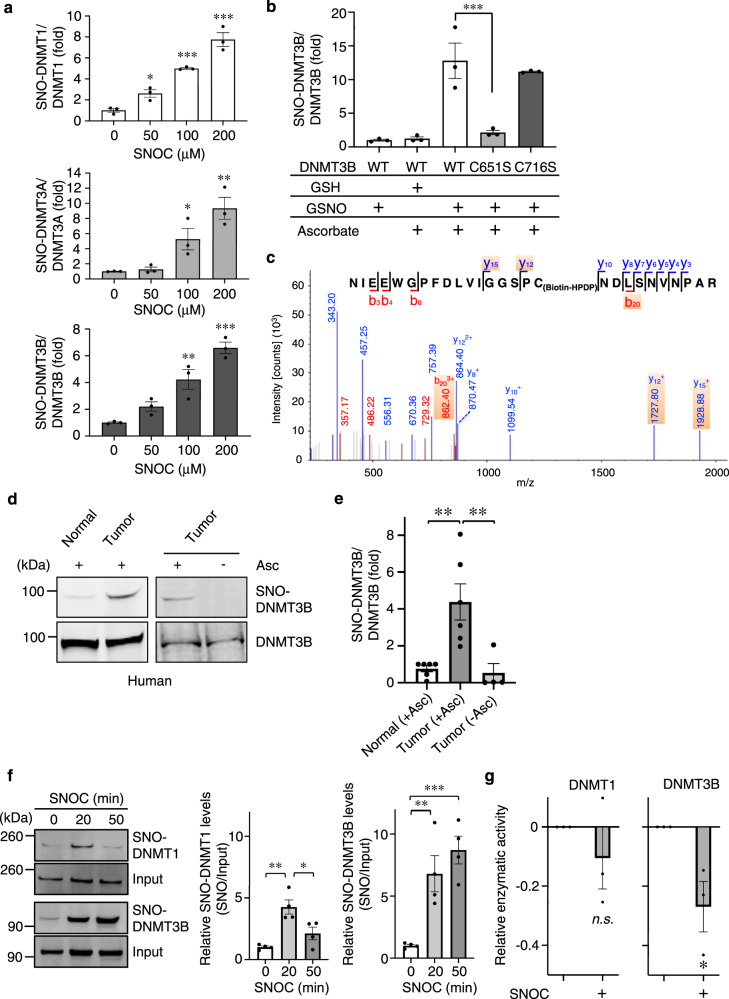
NO regulates DNMT3B activity via *S*-nitrosylation. **a** SNO-DNMT formation after exposure to an NO donor. HEK293 cells were exposed to the indicated concentration of SNOC. After 1 h, SNO-DNMTs were detected by biotin-switch assay. Values are expressed as mean ± s.e.m. (*n* = 3; *P < 0.05, **P < 0.01, ***P < 0.001 vs. SNOC 0 µM by one-way ANOVA with Dunnett’s post hoc test). **b** HEK293 cells, transduced with WT or C-to-S FLAG-tagged DNMT3B mutants, were exposed to 100 μM GSNO or GSH. After 1 h, SNO-DNMT3B was detected using biotin-switch assay with anti-FLAG antibody. Densitometric quantification of SNO-DNMT3B/total DNMT3B formation from biotin-switch and immunoblot data, as illustrated in Supplementary Fig. 1e. Values are mean ± s.e.m. (*n* = 3; ***P < 0.001 by one-way ANOVA with Bonferroni’s post hoc test). **c** Mass spectrometry identification of *S*-nitrosylated Cys-containing peptide in full-length DNMT3B. Representative annotated LC-MS/MS spectrum of biotin-labeled Cys651-containing peptide after trypsin digestion. **d**, **e** Representative human colon cancer or control tissue subjected to biotin-switch assay to detect SNO-DNMT3B that occurred in vivo (**d**). Ratio of human SNO-DNMT3B/total DNMT3B from biotin-switch assay and immunoblot analysis, respectively, quantified by densitometry (**e**). Values are mean ± s.e.m. (*n* = 4–6; **P < 0.01 by one-way ANOVA with Tukey’s post hoc test). **f** Differential temporal levels of *S*-nitrosylation of DNMT1 and DNMT3B in cell-based assays. For cell-based assays, HEK293T cells expressing myc-tagged DNMT1 or DNMT3B were exposed to 200 μM SNOC. After 20 and 50 min, nuclear extracts were isolated, and SNO-DNMTs were detected by biotin-switch assay. Ratio of SNO-DNMT/total DNMT quantified by densitometry. Values are expressed as mean ± s.e.m. (*n* = 4; *P < 0.05, **P < 0.01 by one-way ANOVA with Tukey’s post hoc test). **g** Effect of SNOC on DNMT activity in HEK293T cells. HEK293T cells expressing DNMT1 or DNMT3B were exposed to SNOC. Thirty minutes later, nuclear extracts were isolated, and DNMT enzymatic activity was determined after approximately 1 h. Values are mean ± s.e.m. relative to basal activity (*n* = 3; *P < 0.05, by two-tailed Student’s *t*-test). Source data are provided as a Source data file.

We initially tested whether DNMTs were *S*-nitrosylated using the physiological NO donor *S*-nitrosocysteine (SNOC) in HEK293 cells that had been transfected with each subtype of DNMT. In biotin-switch assays we demonstrated that exposure to SNOC markedly enhanced *S*-nitrosylation of DNMTs (forming SNO-DNMT); importantly, the effect of SNOC was concentration-dependent (Fig. 1a, Supplementary Fig. 1a)^19^. In addition, *S*-nitrosylation of endogenous DNMTs was observed (Supplementary Fig. 1b). We also detected SNO-DNMT3B formation using purified recombinant DNMT3B and found a significant increase in SNO-DNMT3B after exposure to SNOC, suggesting that SNOC can directly modify DNMT3B (Supplementary Fig. 1c, d). We hypothesized that NO-related species may modulate DNMT activity through *S*-nitrosylation. We focused on the Cys residue at position 651 in the catalytic domain of DNMT3B because it is located in proximity to a partial motif for *S*-nitrosylation^20,21^. To determine if indeed this residue is a target of *S*-nitrosylation, we substituted a serine (Ser) for the Cys and assessed SNO-DNMT3B formation by performing biotin-switch assays. After 24 h, cells were exposed to the physiological NO donor *S*-nitrosoglutathione (GSNO), used here because it has a longer half-life than SNOC, or, as a control, to glutathione (GSH). We found that the DNMT3B (C651S) mutant nearly completely abrogated *S*-nitrosylation (Fig. 1b, Supplementary Fig. 1e). Identification of the *S*-nitrosylation site by biotin-switch assay using full-length wild-type (WT) DNMT3B was subsequently confirmed by liquid chromatography coupled with tandem mass spectrometry (LC-MS/MS). MS/MS analysis verified that Cys651 was modified by biotin after exposure to SNOC and replacement of the-SNO moiety by biotin (Fig. 1c). These results are consistent with the notion that Cys651 is the predominant *S*-nitrosylation site on DNMT3B.

To investigate whether endogenously generated NO-related species can also induce SNO-DNMT formation, we used HEK cells stably expressing NOS1 (nNOS). DNMT3B was *S*-nitrosylated by endogenous NO in response to A23187 (to induce calcium-activation of nNOS) in a NOS inhibitor-sensitive manner (Supplementary Fig. 1f). Furthermore, in RAW cells we assessed if NO species derived from NOS2 (iNOS), induced by treatment with lipopolysaccharide (LPS) plus IFN-γ, could *S*-nitrosylate DNMT3B. In this paradigm, we also detected SNO-DNMT3B formation in a NOS inhibitor-dependent manner (Supplementary Fig. 1g). To demonstrate relevance to human carcinomas, we next examined freshly-obtained colon cancer tissues from surgical specimens (Supplementary Fig. 1h). Indeed, we found evidence for SNO-DNMT3B in human colon cancer but not in control tissue (Fig. 1d, e).

Next, we studied the effect of *S*-nitrosylation on DNMT enzymatic activity. Using recombinant DNMT enzymes in an in vitro assay, we found that NO-related species induced SNO-DNMT3B formation and attenuated enzymatic activity (i.e., DNA methylation) in a concentration-dependent manner (Supplementary Figs. 1i, j and 2a), and treatment with the chemical reducing agent dithiothreitol (DTT) abrogated the NO-induced inhibition of DNMT activity under our conditions (Supplementary Fig. 2b), but see refs. ^22,23^. However, when we tested the effect of *S*-nitrosylation on DNMT activity in more physiological cell-based assays, we obtained a different set of results. Strikingly, only DNMT3B but not DNMT1 enzymatic activity was significantly inhibited by *S*-nitrosylation in these cell-based assays. To account for this finding, we found that exposure to SNOC resulted in some degree of *S*-nitrosylation of both DNMT1 and DNMT3B in the cells (Fig. 1f) but noted less nitrosylation and loss of the nitrosylation signal in DNMT1 with time in the cell-based assays (Supplementary Fig. 2c), possibly because of the endogenous redox milieu in cells. In contrast, *S*-nitrosylation of DNMT3B was sustained in the cell-based assays, similar to our findings in vitro with recombinant proteins. In parallel cell-based assays, NO significantly inhibited the enzymatic activity of DNMT3B but not DNMT1 (Fig. 1g, Supplementary Fig. 2d). These results demonstrate that while both DNMT1 and DNMT3B can be *S*-nitrosylated, only the activity of DNMT3B is significantly affected by this reaction in cell-based activity assays. The relatively small extent and short time course of *S*-nitrosylation of DNMT1 compared to that of DNMT3B (Fig. 1f) can explain this lack of significant inhibition of DNMT1 enzymatic activity after exposure to NO in cell-based assays. Moreover, since global methylation is influenced by DNMT1 and not DNMT3B activity, these data predict that *S*-nitrosylation of DNMTs would not affect global methylation in cell-based assays, exactly as we observed (Supplementary Fig. 2e). On the other hand, the persistence of SNO-DNMT3B should affect specific DNMT3B targets of methylation in cells, as we subsequently demonstrate.

Affording further mechanistic insight for these findings, the cysteine residue (C651) that we identified to be *S*-nitrosylated in DNMT3B is known to be in the catalytic site for enzymatic activity^20^. As we have published previously^24^, *S*-nitrosylation of an enzyme’s catalytic site cysteine represents a mechanism of inhibition, and indeed mutation of this catalytic cysteine abrogated enzyme activity similar to *S*-nitrosylation^20^. These findings are consistent with the notion that DNMT enzymatic activity is regulated by redox-mediated posttranslational modification, particularly via reaction with NO-related species to form *S*-nitrosylated DNMT3B.

### Gene expression via DNMT3B dysfunction by NO

DNMT3B is believed to be involved in de novo posttranslational DNA modifications that are associated with the onset of several diseases, including cancers^25^. In order to study the effect of SNO-DNMT3B in this process, we initially conducted a transcriptomic analysis by RNA-sequencing (RNA-seq) to detect genes that were up- or down-regulated after NO exposure in HeLa cell-based assays. This list of these genes is shown in volcano plots (Supplementary Fig. 3a–c). Within 24 h of exposure to an NO donor, we detected 173 genes that were changed >1.5-fold with *P* < 0.05. To confirm these results, we selected four genes (*CA9, EGR1, ALDOC*, and *GPR137C*) that were upregulated by NO and performed quantitative (q)PCR analysis, which confirmed a significant increase in their expression levels (Supplementary Fig. 3d). Notably, with only 173 genes significantly affected, the action of NO appears to be on a rather limited set of genes, not affecting the entire genome.

To confirm this conclusion at an epigenetic level, we next performed DNA methylation profiling using the TruSeq Methyl Capture EPIC Library Prep Kit (Illumina) to elucidate the profile of methylation/demethylation in genome-wide CpG regions in HeLa cells. For each sample, we scored methylated cytosines with ≥10 reads by bisulfite sequence analysis. Of the detected 25,805,689 methylated cytosine sites in the whole genome by this method (Supplementary Data 1), we found a greater than 10% decrease in methylation in only 505,039 (or <2%) of such sites after overexpression of NOS2 (to increase endogenous NO) or exposure to an exogenous NO donor. From these results, we concluded that the vast majority of CpG methylation sites in the whole genome were not changed after exposure to NO, but individual CpG sites were indeed found to be less methylated. Hence, it does not appear that NO affects global methylation status per se. In addition, another index of global DNA methylation employing antibodies to 5-methyl cytosine revealed no significant changes in methylation of whole genomic DNA (Supplementary Fig. 2e). We thus concluded that NO did not affect the vast majority of CpG methylation sites in the whole genome but did result in an increase in individual demethylated CpG sites (Supplementary Figs. 4a–d, 5a, Supplementary Data 1).

Concerning individual genes whose expression is upregulated following NO exposure, we carefully surveyed each such gene for the presence of CpG areas in the 5′-region to determine if there were changes in the methylation/demethylation ratio. As examples, we observed that the vast majority of cytosines in the CpG sites of the genes *CA9*, *EGR1*, *ALDOC*, and *GPR137C* were not demethylated. Some specific sites, however, were found to be demethylated after NOS2 overexpression or SNOC exposure compared to control (Supplementary Figs. 4a–d and 5a). Specifically, the number of demethylated cytosines in the genes *CCND*2, *CA9*, *EG*R1, *ALDOC*, and *GPR137C* was 30, 7, 33, 5, and 20, respectively. In line with these results, recent reports have indicated that the percentage of methylated cytosines in the whole genome of *Dnmt3b*-deficient compared to *Dnmt3b*-WT mice decreased by ~10%, 66.35% compared to 76.94%, respectively^26^. These results suggest that (i) the contribution of DNMT3B may be limited and involves only ~10% of methylation sites in the whole-genome, and (ii) exposure to NO decreased the level of individual methylated cytosines in CpG areas consistent with our finding of inhibition of DNMT3B activity.

**Fig. 2:**
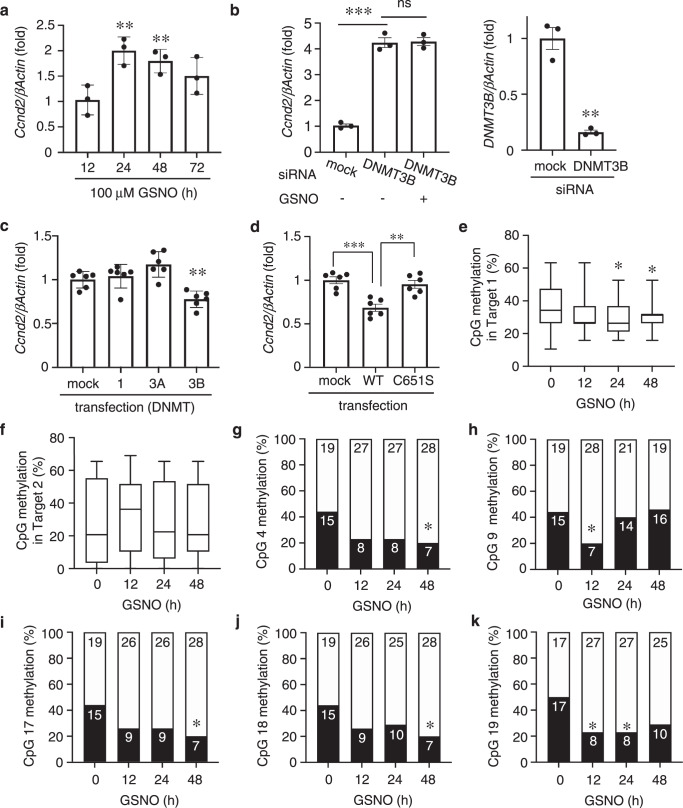
Regulation of Cyclin D2 expression via *S*-nitrosylation of DNMT3B. **a** NO-stimulated Cyclin D2 (*Ccnd2*) mRNA expression. RT-qPCR was performed using specific primers for each mRNA. Values are expressed as mean ± s.e.m. (*n* = 3; **P < 0.01 by two-way ANOVA with Bonferroni’s post hoc test). **b** Left: Level of *Ccnd2* 24 h after exposure to GSNO in cells previously transfected with DNMT3B siRNA or mock control. Values are mean ± s.e.m. (*n* = 3; ***P < 0.001 by one-way ANOVA with Tukey’s post hoc test, ns: not significant). Right: Degree of knockdown of DNMT3B by siRNA vs. mock control (*n* = 3; **P < 0.01 by two-tailed Student’s *t*-test). **c** Cells were transduced with WT DNMT1, DNMT3A or DNMT3B, and then assayed for *Ccnd2* mRNA levels. Values are mean ± s.e.m. (*n* = 6; **P < 0.01 vs. mock by one-way ANOVA with Bonferroni’s post hoc test). **d** HEK cells were transduced with WT or C-to-S mutant DNMT3B and then assayed for *Ccnd2* mRNA levels. Values are mean ± s.e.m. (*n* = 6; **P < 0.01, ***P < 0.001, by one-way ANOVA with Bonferroni’s post hoc test). **e**, **f** HeLa cells were exposed to GSNO and incubated for varying periods. Methylation levels at CpG sites (Targets 1 and 2) within the promoter region of *Ccnd2* were detected by bisulfite sequencing. Experiments were performed using 34–35 samples from triplicate cultures run in parallel. Representative results for the CpG sites of the *Ccnd2* promoter in cells 12–48 h after exposure to GSNO. For box plots, the center lines represent the median, and the box limits are the 25th and 75th percentiles. Whiskers outline minimum to maximum values (*n* = 34–35; *P < 0.05 vs. GSNO 0 h by two-tailed Wilcoxson’s rank-sum test). **g**–**k** Each cytosine in the Target 1 CpG sites (−3621, −3485, −3430, −3426, −3424) was sensitive to NO and significantly less methylated as determined by two-tailed Fisher’s exact (*P < 0.05 vs. GSNO 0 h). Closed bars. methylated CpG sites. Open bars, demethylated CpG sites. Source data are provided as a Source data file.

Our bioinformatic analysis revealed that among the genes that display demethylated CpG sites in response to NO were multiple genes associated with cancer. Hence, we next determined if SNO-DNMT3B formation might enhance mRNA expression encoded by these genes. We initially focused on the cyclin D2 (*Ccnd2*) gene because transcriptional upregulation of this gene and demethylation of CpG in its promoter have been detected in carcinomas, including gastric carcinoma^27,28^. While *Ccnd2* message was not detected by the primer set in the RNA-seq dataset, we found by TruSeq Methyl Capture EPIC analysis that the CpG methylation/demethylation ratio decreased after exposure to NO (Supplementary Fig. 5a). We then monitored temporal expression of *Ccnd2* mRNA in HeLa cells in response to GSNO and found a statistically-significant (~2-fold) elevation in the level of *Ccnd2* mRNA at 24 h. This response to GSNO was sustained for 48 h and then gradually decreased by 72 h (Fig. 2a). In a similar fashion, siRNA knockdown of DNMT3B markedly enhanced the level of *Ccnd2* mRNA (Fig. 2b). Moreover, after DNMT3B knockdown, no further enhancement of *Ccnd2* expression was observed in response to an NO donor (Fig. 2b), consistent with the notion that the major contributor to NO-mediated upregulation of *Ccnd2* involved S-nitrosylation and thus inhibition of DNMT3B rather than another DNMT, such as DNMT3A or DNMT1, which are also present in these cells.

In addition, transduction with DNMT3B, but not DNMT1 or DNMT3A, significantly decreased *Ccnd2* mRNA levels (Fig. 2c). Thus, it is unlikely that *S*-nitrosylation of DNMT3A or DNMT1 could have contributed to the effects on *Ccnd2* associated with tumor formation observed here since altering the level of either of these DNMTs did not influence the level of *Ccnd2* expression. Collectively, these findings are consistent with the notion that DNMT3B is a key enzyme involved in the regulation of *Ccnd2* expression, and inhibition of DNMT3B activity by *S*-nitrosylation results in increased *Ccnd2* at the transcriptional level. To obtain more direct evidence for this conclusion, we investigated the effect of overexpression of non-nitrosylatable DNMT3B (C651S), representing a ‘dead enzyme’, on *Ccnd2* mRNA induction in HEK cells. As expected, compared to WT DNMT3B, overexpression of mutant DNMT3B (C651S) did not inhibit *Ccnd2* mRNA expression^20^ (Fig. 2d).

Next, we measured the methylation/demethylation ratio in various CpG target areas of the *Ccnd2* gene after NOS2 overexpression or NO exposure. We found that the CpG methylation/demethylation ratio in one area (Target 1: −3687 – −3398) was significantly decreased (~25%) within 24 h of exposure to GSNO (Fig. 2e). Moreover, each cytosine in the target region (at locations −3621, −3485, −3430, −3426, −3424) was sensitive to NO (Supplementary Fig. 5b, c), becoming significantly less methylated between 12 to 48 h post GSNO exposure (Fig. 2g–k). Interestingly, in a second target area (Target 2: +642 – +1040), the CpG methylation/demethylation ratio was not greatly affected by GSNO (Fig. 2f). Thus, there may be a heterogeneous response to NO in the DNA methylation/demethylation patterns among different CpG islands within a single gene^29–32^, and such varying patterns have been observed previously after treatment with a DNMT inhibitor^29,30^. These findings are consistent with the notion that some (but not all) CpG sites are targeted by NO-sensitive DNMT3B. Taken together, our data indicate that redox modification of DNMT3B by *S*-nitrosylation can function as a unique and robust regulator of gene expression in cancerous cells.

### Isolation of a specific inhibitor of DNMT3B *S*-nitrosylation

Next, we sought to develop a specific inhibitor of DNMT3B *S*-nitrosylation as a possible therapeutic agent. Hierarchical virtual screening approaches represent an established methodology for filtering complex chemical libraries containing very large numbers of small molecules towards a reasonable number of candidates for further biological testing^33,34^. To this end, in order to identify compounds capable of inhibiting DNMT3B *S*-nitrosylation but not enzymatic activity, we initially created a DNMT3B model based on the known atomic structure of DNMT3A. In the absence of structural information about DNMT3B-DNA binding and Cys651 *S*-nitrosylation, we hypothesized that a compound binding to the loop region, extending from Thr773 to Asn786, might lock DNMT3B in a conformation suitable for DNA binding and subsequent DNA methyltransferase activity but not favorable for Cys651 *S*-nitrosylation. We explored small-molecule binding pockets in the vicinity of Cys651 in three models of DNMT3B. The binding pocket calculations using SiteMap indeed revealed a high-scoring pocket near the loop region from Thr773 to Asn786 in all three DNMT3B models (Supplementary Fig. 6a–e). The Sitescore values of 1.02, 1.05, and 0.91 for the three DNMT3B models indicated the druggability of this region (Supplementary Fig. 6f). We then docked a large collection of chemicals with this model. Virtual screening was carried out separately for all three of these pockets, and the top-ranking compounds from each run were merged and visually analyzed for the interaction they made with the binding pocket. Finally, compounds making interactions with amino-acid residues in the loop region from Thr773 to Asn786 were prioritized for further testing. From 4 × 10^6^ chemicals, we narrowed the candidates to approximately 100 via two independent in silico docking steps.

**Fig. 3:**
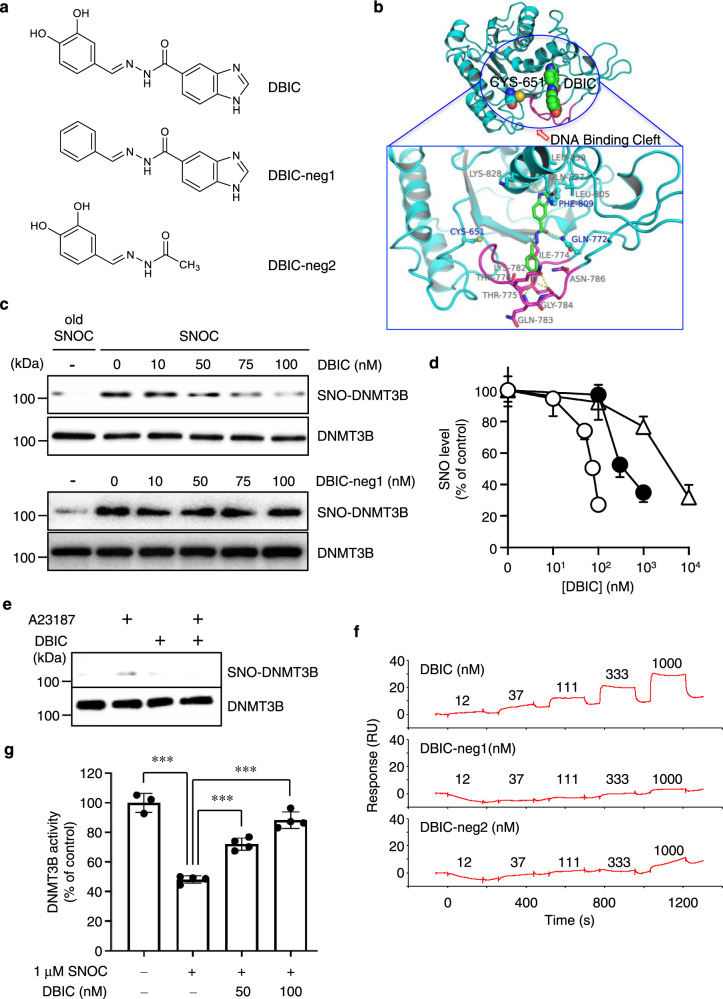
DBIC, a potent inhibitor of SNO-DNMT3 formation. **a** Chemical structures of DBIC and its negative control compounds (DBIC-neg1 and DBIC-neg2). **b** Molecular docking predicted binding mode of DBIC (green) in DNMT3B (cyan). Loop in the region of Thr773 to Asn786 is colored in magenta. **c**, **d** HEK293 cells were preincubated with varying concentrations of DBIC or DBIC-neg1 for 1 h prior to SNOC exposure. SNO-DNMT3B formation was detected by biotin-switch assay. The data shown represent one of three separate experiments as shown in (**d**). Biotin-switch assay and immunoblot analysis were quantified by densitometry; the relative ratio of SNO-DNMT to total DNMT was calculated for each sample (**d**). Values are expressed as mean ± s.e.m. (*n* = 3). Open circles, DNMT3B. Closed circles, DNMT3A. Open triangles, DNMT1. **e** DBIC inhibited SNO-DNMT3B formation generated by endogenous NO in HEK-293 cells stably expressing NOS1. NOS1 was activated by calcium ionophore A23187. **f** Direct binding of DBIC to full-length DNMT3B protein determined by surface plasmon resonance analysis. **g** Effect of DBIC on DNMT3B enzymatic activity attenuated by NO. Values are expressed as mean ± s.e.m. (*n* = 3–4, ***P < 0.001 by one-way ANOVA with Bonferroni’s post hoc test). Source data are provided as a Source data file.

We then tested these compounds for an inhibitory effect on SNO-DNMT3B formation while counterscreening to exclude compounds that affected enzymatic activity. With this paradigm, we identified (*E*)-*N*′-(3,4-dihydroxybenzylidene)-1*H*-benzo[*d*]imidazole-5-carbohydrazide, designated here as DBIC, as fulfilling these criteria (Fig. 3a). Furthermore, molecular docking showed that the catechol and benzimidazole moieties are essential for the interaction between DBIC and DNMT3B (Fig. 3b). Indeed, we demonstrated that loss of the two hydroxyl groups of the catechol (compound DBIC-neg1) or the benzimidazole moiety (compound DBIC-neg2) completely abolished the inhibitory effect on SNO-DNMT3B formation (Fig. 3c, Supplementary Fig. 7a, c). Moreover, DBIC inhibited SNO-DNMT3B formation in a concentration-dependent manner with an IC_50_ value of ~75 nM. While DBIC also impaired *S*-nitrosylation of DNMT1 and DNMT3A, this only occurred at IC_50_ values greater than ~50 μM and ~500 nM, respectively (Fig. 3c, d). In cells, DBIC also attenuated the formation of SNO-DNMT3B induced by endogenous NO (Fig. 3e). In addition, the DNMT3B double mutant (Q772A/F809A), designed to disrupt binding to DBIC, led to the loss of the DBIC inhibitory effect on SNO-DNMT3B formation (Supplementary Fig. 7d).

Next, we employed surface plasmon resonance analysis to assess direct binding of DBIC to DNMT3B protein (Fig. 3f). DBIC manifested binding to immobilized DNMT3B in a concentration-dependent manner and at concentrations as low as 12.3 nM, the lowest concentration used in our experiments. In contrast, DBIC neg-1 and DBIC neg-2 displayed a much lower binding response to DNMT3B than DBIC. These observations suggest that DBIC directly binds to DNMT3B with higher affinity compared to the inactive DBIC derivatives. Critically, at 50–100 nM DBIC prevented formation of SNO-DNMT3B and inhibition of DNMT3B enzymatic activity in the presence of an NO donor (Supplementary Fig. 7e, Fig. 3g).

The core scaffold of DBIC, benzylidene carbohydrazide, is well-known among structures that are pan-assay interference compounds (PAINS), which are usually avoided in drug discovery efforts. Although it is generally accepted that a compound can be predicted as a potential PAINS from its core scaffold, it is also well-known that the chemical properties of a compound can be significantly modified by substitutions on the core scaffold, thus rendering the compound “PAINless.” For example, Baell and Holloway considered the hydroxyphenylhydrazone scaffold, which shares the core structure of benzylidene carbohydrazide^35^. Hydroxyphenylhydrazone scaffold-containing compounds have also been identified as PAINS. However, they described differences between 2- and 4-hydroxyphenylhydrazones, highlighting the effect of substitutions on the core scaffold and how it can render the compound PAINless. Similarly, DBIC contains a 3,4-dihydroxyphenylhydrazone core and an additional *N*-acyl substitution. In fact, this class of *N*-acylhydrazone (NAH)-containing compounds are found in FDA-approved drugs and in several clinical trial candidates that have shown both safety and efficacy^36^. These prior publications highlight the fact that not all NAH-containing compounds are PAINS. Specifically, for DBIC we found that removing the dihydroxyl group (DBIC-neg1) or the benzimidazole group (DBIC-neg2), while retaining the NAH scaffold, rendered the compounds inactive, demonstrating that the NAH core per se in DBIC is not causing PAINS.

In addition, to corroborate these findings we derived a second active compound lacking PAINS structure (Supplementary Fig. 7f). We found that this compound (*N*-(2-(3,4-dihydroxyphenyl)ethyl)-1H-benzo[d]imidazole-6-carboxamide), designated DBIC-der1, can also effectively attenuate SNO-DNMT3B formation in cells (Supplementary Fig. 7g, h). Moreover, in silico simulation revealed that the new derivative (DBIC-der1) can associate with the same amino acids as observed for DBIC itself. Thus, we report that both DBIC and DBIC-der1 can bind to DNMT3B protein to prevent *S*-nitrosylation. We conclude that in this particular case, the PAINS structure of DBIC is involved in a specific interaction with DNMT3B.

Considering potential off-target effects of DBIC, we had previously demonstrated that phosphatase and tensin homolog (PTEN), another protein implicated in the etiology of many cancers as a tumor suppressor, is highly sensitive to *S*-nitrosylation by donation of NO species;^7^ however, unlike its action on DNMT3B, DBIC did not affect the formation of SNO-PTEN (Supplementary Fig. 7b). In addition, in a screen for off-target effects, high concentrations of DBIC (≥10 μM) did not substantially stimulate or inhibit a large number of G-protein coupled receptors (GPCRs), nuclear hormone receptors, transporters, or ion channels in cell-based assays (Supplementary Fig. 8a). We found that treatment of cells with 100 nM DBIC inhibited DNMT3B *S*-nitrosylation without affecting enzymatic activity; however, at very high concentrations (>10 μM), DBIC slightly inhibited DNMT3B activity (Supplementary Fig. 8b). In contrast to DBIC, theaflavin-3,3′-digallate (TF-3), a phenol antioxidant and non-selective DNMT3 inhibitor, strongly disrupted DNMT3B enzymatic activity^37^. We also considered the possibility that the inhibitory effect of DBIC on *S*-nitrosylation might be due to its scavenging ability; however, we found that DBIC, even at micromolar concentrations, did not function as a NO scavenger (Supplementary Fig. 8c). Moreover, at 10 µM, representing 100-times the therapeutic concentration, DBIC manifested no cytotoxicity on several normal cell types (Supplementary Fig. 8d). Thus, these results indicate that DBIC is a unique compound that at low concentrations specifically inhibits SNO-DNMT3B formation without affecting enzyme activity and in the absence of cell toxicity.

As shown above, we found that NO donors could stimulate *Ccnd2* induction via a decrease in the methylation/demethylation ratio of the promoter CpG region in HeLa cells (Fig. 2a, g–k). In contrast, DBIC significantly abrogated NO-induced *Ccnd2* mRNA expression in HeLa cells (Supplementary Fig. 9a). Moreover, at 100 nM DBIC prevented the increase in tumor cell number induced by NO; as a control, DBIC-neg1 had no such effect (Supplementary Fig. 9b). In addition, DBIC significantly ameliorated the decrease in the methylated CpG ratio in the *Ccnd2* promoter engendered by NO (Supplementary Fig. 9c, d).

### Pharmacological characterization of DBIC

**Fig. 4:**
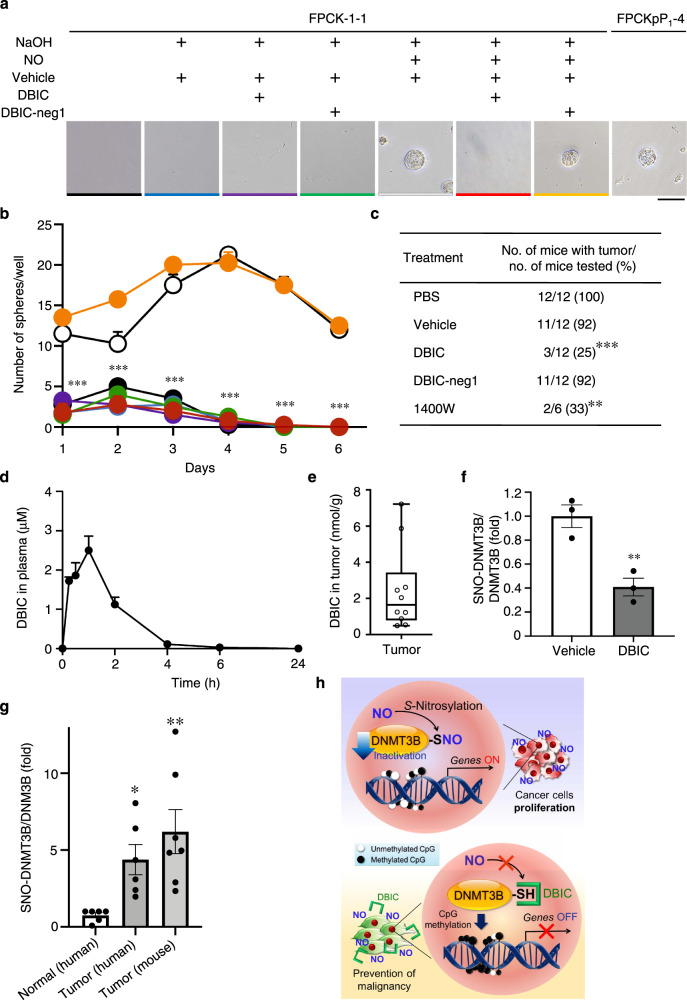
DBIC suppresses NO-induced tumor formation. **a** FPCK-1-1 cells were treated with 100 nM DBIC, DBIC-neg1, or 126 μM NOC18 every 3 days for a month before transfer to 3D culture. Spheroidal aggregate formation of FPCK-1-1 (or FPCKpP1-4 control) cells was assessed 3 d after plating. Scale bar, 50 μm. The data represent one of four separate experiments. **b** DBIC inhibited NO-induced sphere formation assessed 7 days post plating. Values are mean ± s.e.m. (*n* = 4; ***P < 0.001 by two-way ANOVA with Bonferroni’s post hoc test). Treatments: None (black); vehicle (blue); DBIC (purple); DBIC-neg1 (green); NO (white); NO + DBIC (red); NO + DBIC-neg1 (orange). Colors correspond to code in representative micrographs in (**a**). **c** DBIC inhibits inflammation-related carcinogenesis in vivo. DBIC, DBIC-neg1 (25 mg/kg/day), or 1400 W (6 mg/kg/day) administered by i.p. injection from pre-implantation day 2 through day 35 post cell implantation. Regressive QR-32 cells (1 × 10^5^) were implanted into mice on day 0 in a subcutaneously pre-inserted foreign body (gelatin sponge, 10 × 5 × 3 mm). **P < 0.01, ***P < 0.001 vs. vehicle by χ^2^-test. **d** Time course of DBIC concentration in plasma after single i.p. administration (25 mg/kg) measured by LC-MS/MS. Values are mean ± s.e.m. (*n* = 3). **e** Concentration of DBIC i*n* tumor tissue after 25 d of daily administration. Samples were analyzed 1 h after the final dose. For box plots, center line represents median; box limits are 25th and 75th percentiles. Whiskers represent minimum-maximum values (*n* = 10). **f** SNO-DNMT3B in tumor tissue. Values are mean ± s.e.m. (*n* = 3; **P < 0.01 by two-tailed Student’s *t*-test). **g** Relative ratio of SNO-DNMT3B in human and mouse tumors. Biotin-switch and immunoblot assays were quantified by densitometry to calculate relative ratio of SNO-DNMT3B to total DNMT3B. Values are mean ± s.e.m. (*n* = 6–7; *P < 0.05, **P < 0.01 vs Normal by o*n*e-way ANOVA with uncorrected Fisher’s LSD post hoc test). **h** Schematic of SNO-DNMT mechanism of action on expression of specific genes associated with neoplasia via decreased methylation of CpG sites. Source data are provided as a Source data file.

We next investigated if DBIC could inhibit tumor formation, as RNS are known to play a role in the conversion of adenoma to adenocarcinoma in a NOS inhibitor-sensitive manner in human colonic adenoma (FPCK-1-1) cells and in mouse regressive fibrosarcoma (QR-32) cells^38^. We observed significant tumorigenic conversion in FPCK-1-1 cells after exposure to the long-lived NO donor NOC18; large spherical clusters of tumor cells formed in three-dimensional (3D) multicellular cultures (Fig. 4a, b, Supplementary Fig. 10a). Subsequently, both control and vehicle-treated cells underwent anoikis. Strikingly, treatment with DBIC, but not DBIC-neg1, significantly blocked spheroid formation induced by NO in the FPCK-1-1 cells and QR-32 cells (Fig. 4a, b, Supplementary Fig. 10a). Next, we characterized the effect of DBIC on tumor formation in vivo in an inflammation-related mouse model of carcinogenesis^39,40^. For this purpose, we used the regressive tumor cell line QR-32, which grows to lethal proportions after being implanted subcutaneously within a foreign body to induce inflammation^39^. Intraperitoneal (i.p.) administration (25 mg/kg/day for 15 days) of DBIC or 1400 W (NOS2 specific inhibitor), but not DBIC-neg1, significantly decreased the proportion of mice bearing tumors (Fig. 4c).

We then evaluated in vivo pharmacokinetic (PK) properties of DBIC in a mouse model of inflammation-related carcinogenesis (ICR) (Fig. 4d, Supplementary Fig. 10b). A single i.p. administration of DBIC (25 mg/kg) resulted in a plasma maximum concentration (Cmax) of 2.5 μM, which appears to be sufficient for inhibiting tumor formation from our data. Within 6 h of administration, DBIC was nearly completely depleted from the blood (Fig. 4d). This finding may reflect good tissue transportability of the compound because of its large volume of distribution at steady state (Vdss) of 23.8 L/kg (Supplementary Fig. 10b). Indeed, the concentration of DBIC in tumor tissue (mean of 2.5 nmol/g) after continuous i.p. administration was nearly equivalent to the plasma Cmax (Fig. 4e). Despite only partial inhibition of DNMT3B by once-daily i.p. treatment with DBIC, this was sufficient to induce significant inhibition of tumor incidence in treated mice. Hence, improved in vivo dosing of would be predicted to reduce the tumor incidence even further. Moreover, DBIC caused no obvious toxicity (such as weight loss) or alteration in the appearance or behavior of tumor-bearing mice (Supplementary Fig. 10c).

Furthermore, we found that DBIC abrogated formation of SNO-DNMT3B within tumor tissue isolated in vivo from the mouse model (Fig. 4f, Supplementary Fig. 10d). Finally, to determine whether the level of SNO-DNMT3B detected in human colon cancer is of pathophysiological relevance, we calculated the ratio of SNO-DNMT3B (determined by biotin-switch assays) to total DNMT3B (from immunoblots) and found that the ratio in human cancer tissue was similar to that encountered in our in vitro and in vivo cancer models (Figs. 1e, 4g, Supplementary Figs. 10e, f). This finding indicates that pathophysiologically-relevant amounts of SNO-DNMT3B are present in human colon cancers. Collectively, our findings show that DNA methylation is regulated, at least in part, by *S*-nitrosylation of DNMT, forming SNO-DNMT. The resulting decreased methylation within selected promoter CpG sites affects expression of specific genes associated with neoplastic transformation such as *Ccnd2*. A recent study reported that NO modulates DNMT1 degradation in models of breast cancer, but did not find an effect of *S*-nitrosylation of DNMT1 per se on methylation status^41^.

In the present study, we demonstrate that SNO-DNMT3B inhibited its enzymatic activity in cell-based assays, whereas S-nitrosylation of DNMT1 did not affect its enzymatic activity in cell-based or in vivo colon cancer model systems. Moreover, we describe a unique modulator of DNMT3B that can specifically attenuate its modification by SNO, but not its enzymatic activity (Fig. 4h). This chemical modulator and potential therapeutic, designated DBIC, significantly mitigated cell proliferation and tumorigenic conversion in vivo. Prior publications had reported the importance of NO and epigenetic regulation to tumorigenesis^42,43^; however, the link between these two remained unknown. In the present study, we link these events in a mechanistic fashion that also provides important therapeutic insight.

## Methods

### Relevant ethical regulations

Animal experiments were conducted in accordance with the animal experimental protocol and guidelines of the Tokyo University of Pharmacy and Life Sciences Animal Experimentation Regulations after review by the Institutional Animal Care and Use Committee (permission numbers L18-10 and L19-26) and approval by the President of the Tokyo University of Pharmacy and Life Sciences. The experimental protocol (carcinogenesis model) was approved by the Committee of the Institute for Animal Experimentation of Tottori University (14-Y-14).

### Materials

NO donors, *S*-nitrosocysteine (SNOC) and *S*-nitrosoglutathione (GSNO), were prepared freshly before use and kept under dark conditions. SNOC was prepared as previously published^44^. In order to dissipate NO from SNOC (to produce ‘old’ SNOC as a control), SNOC was left at RT for at least 24 h. A23187, *N*^G^-Nitro-l-arginine methyl ester (l-NAME), lipopolysaccharide (LPS from *S. minnesota* R595), and theaflavin 3,3′-digallate were purchased from Wako Pure Chemical Industry, Ltd. Methyl methanethiosulphonate (MMTS) and 5-aza-2′-deoxycytidine (5-Aza) were purchased from Tokyo Chemical Industry, Co., Ltd. Mouse interferon-γ was purchased from PeproTech. *N*-[6-(biotinamido)hexyl]-3′-(2′-pyridyldithio) propionamide (HPDP-biotin) was purchased from Thermo Fisher Scientific. Western blot analysis was performed using the following specific primary antibodies; anti-FLAG M2 covalently conjugated to horseradish peroxidase (HRP) (Sigma-Aldrich, clone M2, 1:50,000), anti-NOS2 (Millipore, ABN26, 1/10,000), anti-myc (Cell Signaling Technology, 71D10, 2278S, 1:1000), and anti-PTEN (Cell Signaling Technology, 138G6, 1:5000). The following primary antibodies were diluted with Can Get Signal Solution 1 (TOYOBO) to improve sensitivity and specificity; anti-GFP (Nacalai Tesque, GF200, 1:4000), anti-DNMT1 (Cell Signaling Technology, D59A4, 1:10,000), anti-DNMT3A (Sigma-Aldrich, D8695, 1:3000), and anti-DNMT3B antiserum (1:4000). Anti-DNMT3B antiserum was produced by immunization of rabbits with the KLH (keyhole limpet hemocyanin)-conjugated peptide derived from N-terminus of human DNMT3B (MKGDTRHLNGEEDAGGRC) (Sigma Genosys). Secondary antibodies used included anti-rabbit IgG HRP-linked F(ab′)_2_ fragment from donkey (GE Healthcare, NA9340, 1:25,000), anti-mouse IgG HRP-linked F(ab′)_2_ fragment from sheep (GE Healthcare, NA9310, 1:25,000), and IRDye® 800CW goat anti-rabbit IgG secondary antibody (LI-COR, 926-32211, 1:15,000). Full-length WT DNMT3B (NP_787045.1) was purchased from Active Motif Inc. (Carlsbad, CA, USA).

### Cell culture

Human embryonic kidney (HEK) 293T cells, human gastric adenocarcinoma AGS cells, human cervical carcinoma (HeLa) cells, and mouse macrophage RAW264.7 cells were maintained in Dulbecco’s modified Eagle’s medium (DMEM; Wako Pure Chemical Industry or Thermo Fisher Scientific) supplemented with 10% (v/v) heat-inactivated fatal bovine serum (FBS; Biosera or Sigma) and 1% penicillin/streptomycin (Wako Pure Chemical Industry) at 37 °C in a humidified atmosphere of 5% CO_2_/95% air. Human colon adenoma FPCK-1-1 cells and adenocarcinoma FPCKpP1-4 cells were maintained in a mixture of 6052 medium and DM-160 (Kyokuto Pharmaceutical Industrial Co., Ltd.) supplemented with 1% dialyzed FBS, ITS premix (354350, Becton Dickinson) and 10 ng/ml human epidermal growth factor (EGF0501, ATGen) at 37 °C in a humidified atmosphere of 5% CO_2_/95% air. Mouse regressive fibrosarcoma QR-32 cells and its derived tumorigenic QRsP-11 cells were maintained in Eagle’s minimum essential medium (EMEM; Nissui) supplemented with 8% FBS, sodium pyruvate (Wako Pure Chemical Industry) and L-glutamine (Wako Pure Chemical Industry) in a humidified atmosphere of 5% CO_2_/95% air.

### Mouse and human tissue specimens

C57BL/6 mice (5-week-old females) obtained from Nippon SLC (Hamamatsu) were maintained under specific-pathogen-free (SPF) conditions with a photoperiod of 7:00 a.m. to 7:00 p.m. at 23 ± 3 °C and 50% ± 10% humidity in the Institute for Animal Experimentation of Tottori University and used after one week acclimatization. Mice were fed with a basal diet (Oriental Yeast Co., Ltd.). The experimental protocol was approved by the Committee of the Institute for Animal Experimentation of Tottori University (14-Y-14). Human colon cancer specimens were obtained from Origene, Inc. The OriGene Tissue Biorepository is comprised of a comprehensive library of over 120,000 high-quality human biospecimens representing over 12,000 donor cases. Tissue was collected from USbased large academic medical centers, and samples were acquired only after going through the stringent IRB-approval (Institutional Review Board) process at each medical center. While consent forms were not provided to OriGene, quality assurance mechanisms were instituted to validate compliance with bioethics policies for patient protection. All samples were banked under what is called the “Common Rule” (Federal policy for the protection of human subjects, 45 CFR 46), and data collected were HIPAA compliant. The collections and storage standard operation procedures are compliant with all existing federal, state, local, and institutional requirements. All tissue samples were excised by licensed medical doctors. All materials and associated clinical information are coded and any patient identifiers removed so that no donor can be identified by OriGene or medical researchers. All samples and clinical data were collected and stored within HIPAA guidelines. As the patient samples were de-identified, this is non-human subject research.

### Plasmids and mutagenesis

Human DNMT1 and human DNMT3A cloned into pCAG-EGFP were kindly provided by Dr. Isao Suetake^45,46^. Human DNMT3B1 cloned into p3 × FLAG-CMV10 was kindly provided by Drs. Motoka Unoki and Hiroyuki Sasaki^47^. Mutants of DNMT3B were generated by substituting serine for cysteine using the QuickChange Site-Directed Mutagenesis Kit (Agilent Technologies) according to the manufacturer’s instructions using the following primers: C651S mutant 5′-GAT TGG CGG AAG CCC AAG CAA CGA TCT CTC AAA TG-3′ and 5′-CAT TTG AGA GAT CGT TGC TTG GGC TTC CGC CAA TC-3′, C716S mutant 5′-CAT CTC ACG GTT CCT GGA GAG TAA TCC AGT GAT TG-3′ and 5′-CAA TCA CTG GAT TAC TCT CCA GGA ACC GTG AGA TG-3′. A DNMT3B Q772A/F809A double mutant was generated by the megaprimer method using PrimerStar MAX Premix (Takara). F809A mutant 5′-CTC GAA AGG ATC GCT GGC TTT CCT GTG-3′ and 5′-GGG AGA TCT CTA TTC ACA TGC AAA G-3′, Q772A mutant 5′-CCC CTC GAG CTG CAG GAC TGC TTG GAA TAC AAT AGG ATA GCC AAG TTA AAG AAA GTA GAG ACA ATA ACC AAG-3′. All constructs were verified by sequencing. pcDNA3/Myc-DNMT1 (Addgene plasmid # 36939) and pcDNA3/Myc-DNMT3B1 (Addgene plasmid # 35522) were a gift from Arthur Riggs^48^. Each plasmid was transiently introduced into cells with polyethylene imine (PEI)-max (Polysciences) or Lipofectamine 2000 (Thermo Fisher Scientific), and the *S*-nitrosylation of proteins was analyzed after 24 h.

### Activation of NO synthase (NOS) and measurement of formation of endogenous NO-related species

To induce NOS2 in RAW264.7 cells, cultured medium was replaced to serum-free DMEM supplemented with LPS (10 μg/ml) and mouse interferon-γ (100 units/ml). Cells were stimulated for 24 h in the presence or absence of l-NAME (1 mM). HEK293T cells expressing human NOS1 were exposed to Ca^2+^ ionophore A23187 (10 μM) for 6 h with or without l-NAME (1 mM). To monitor the production of RNS, nitrite/nitrate in the medium was measured by the Griess assay. Briefly, the cultured medium was reacted with Griess reagent (2% sulfanilamide) and 0.2% *N*-(1-naphthyl)-ethylenediamine dihydrochloride in 1.2 M HCl) in 96-well plates for 15 min at RT. The optical density at 540 nm was then measured with a Model 680 microplate reader (Bio-Rad Laboratories).

### Biotin-switch assay for detection of *S*-nitrosylated proteins

Biotin-switch assays were performed as described previously^22,49^, and procedures were performed under dark conditions. Fresh SNOC or GSNO was diluted in 10 mM HEPES-NaOH (pH 7.7). HEK293T cells were pretreated with the compound DBIC, DBIC-neg, or DBIC-der1 for 1 h prior to stimulation with NO donors. After washing the cells with phosphate-buffered saline (PBS), cell lysates were prepared in HEN buffer [250 mM HEPES-NaOH (pH 7.7), 1 mM EDTA, 0.1 mM neocuproine] supplemented with 1% Triton X-100 (Sigma-Aldrich) and protease inhibitor cocktail (Roche). Blocking buffer (2.5% SDS, 50 mM MMTS in HEN buffer) was mixed with the samples and further incubated for 20 min at 50 °C to block free thiol groups. To remove excess MMTS, pre-chilled acetone was added to the samples. After a 30 min incubation at −20 °C, precipitated proteins were centrifuged at 3200 × *g* for 10 min at 4 °C. Proteins were rinsed twice with 70% acetone and dissolved in HENS buffer (1% SDS in HEN buffer). *S*-Nitrosothiols were then reduced to free thiols with 10 mM fresh sodium ascorbate solution. Newly formed thiols were linked with 2 mM HPDP-biotin (Thermo Fisher Scientific) or Biotin-HPDP(WS) (Dojindo Molecular Technologies) for 1 h at RT. Biotinylated proteins were precipitated with pre-chilled acetone for 30 min at −20 °C and dissolved in HENS buffer. Biotinylated proteins were pulled down with equilibrated streptavidin-agarose beads (Thermo Fisher Scientific) for 12 h at 4 °C. Beads were washed five times with high-salt neutralization buffer [20 mM HEPES-NaOH (pH 7.7), 600 mM NaCl, 1 mM EDTA, 0.5% Triton X-100] and three times with neutralization buffer [20 mM HEPES-NaOH (pH 7.7), 100 mM NaCl, 1 mM EDTA, 0.5% Triton X-100]. Biotinylated proteins were then eluted from beads using 2 × SDS-PAGE sample buffer, and samples were analyzed by immunoblotting.

### LC/MS/MS analysis

Human recombinant full-length WT DNMT3B (N-terminal 6x His-tagged, 8 µg; Active Motif) was incubated with or without 100 µM SNOC and analyzed 30 min later. Biotin-switch assay was performed as above. From the reaction mixture incubated in HPDP-biotin, DNMT3B was purified with a His-tagged Protein Purification Kit. The eluted protein was treated with 0.25 µg/ml trypsin (Promega) at 37 °C overnight, the digested peptides were cleaned with self-made C18 and SCX (3 M Empore Solid Phase Extraction Disk) StageTips, and then processed by liquid chromatography (EASY-nLC 1000) (Thermo Fisher Scientific) coupled to a Q-Exactive hybrid quadrupole-orbitrap mass spectrometer (MS, Thermo Fisher Scientific) with a nanospray ion source in positive mode. The peptides were separated with a nano-HPLC C18 capillary column (0.075 × 150 mm, 3 mm) (Nikkyo Technos). A 120-min gradient was used at a flow rate of 300 nl/min, including 0–30%B in 100 min and then 30–65%B in 20 min (solvent A, 0.1% formic acid; solvent B, 100% CH3CN, 0.1% formic acid). MS and MS/MS scans were acquired with a resolution of 70,000 and 17,500, respectively. The top ten precursor ions were selected for MS/MS by higher energy collisional dissociation (HCD) fragmentation at 28% normalized collision energy. The resulting MS and MS/MS data were used to search the human Swiss Prot database using Proteome Discoverer (version 1.4, Thermo Fisher Scientific) with MASCOT search engine software (version 2.6.0, Matrix Science). Parameters were set as follows: enzyme: trypsin (C-term side of K/R), the maximum number of missed cleavages = 1, variable modifications: oxidation (Met), biotin-HPDP (Cys), methylthio (Cys), cysteinyl (Cys), mass tolerance for precursor = 6 ppm, mass tolerance for fragment ions = 20 mmu, minimum peptide length 4. The proteins were considered to be identified when they have at least one peptide with 5% FDR. This analysis was performed once (*n* = 1). The reliability of this experiment is supported by other reinforcing data.

### RNA interference analysis

The following siRNA specific to human DNMT3B were used: 5′-CAC AGG ACU UGA CAG GCG ATT-3′; 5′-UCG CCU GUC AAG UCC UGU GTT-3′ and 5′-GCA UAA AGG UAG GAA AGU ATT-3′; 5′-UAC UUU CCU ACC UUU AUG CTT-3′. This DNMT3B siRNA was designed and synthesized by Sigma Genosys. MISSION siRNA Universal Negative Control for humans (Sigma-Aldrich) was used as a control siRNA. In this case, AGS cells were reversely transfected with 100 pmol of control siRNA/DNMT3B siRNA using Lipofectamine RNAiMAX (Thermo Fisher Scientific) in 6-well plates, and incubated for 48 h.

### In vitro DNMT activity assay

Recombinant DNMT protein (200–400 ng) was used to assess DNA methyltransferase activity against CpG-rich DNA substrates with a DNMT Direct Activity Assay Kit (BPS Biosciences, catalog #52035). In brief, each DNMT isoform (DNMT1: aa 2–1632 [end]; DNMT3A: aa 623–912 [end]; DNMT3B: aa 564–853 [end]) was incubated with enzyme reaction buffer, the indicated concentrations of decayed/fresh SNOC, and *S*-adenosylmethionine (40 μM) in DNA substrate-coated 96-well plates for 2 h at 37 °C. Theaflavin 3,3′-digallate (10 μM), which is a non-nucleoside DNMT inhibitor, was used as a positive control. After the reaction, methylated DNA substrates were labeled with an anti-5-methylcytosine antibody for 1 h at RT. Finally, samples were treated with an HRP-conjugated secondary antibody for 30 min at RT, followed by the addition of ECL substrates. Chemiluminescence intensity was measured with the Tristar2 LB942 multi-detection microplate reader (BERTHOLD).

### Cell-based nuclear DNMT activity assay

HEK293T cells transfected with myc-tagged DNMT1 or DNMT3B were exposed to SNOC. After 30 min, nuclear proteins were isolated using a published protocol^50^. DNMT activity of freshly prepared nuclear extracts (5 μg) was measured using a Fluorometric EpiQuik DNMT Activity Assay Ultra Kit (Epigentek, P-3010-96) per the manufacturer’s instructions with modification. To perform the DNMT enzymatic reaction under non-reducing conditions, freshly prepared reaction buffer (5% glycerol, 1 mM EDTA, 20 mM Tris HCl (pH 7.4), and 25 mM NaCl) was used instead of the manufacture’s assay buffer in order to avoid reducing equivalents that could affect protein *S*-nitrosylation. The fluorescence intensity reflecting DNMT activity was measured using 530-nm excitation and 590-nm emission readings from a SpectraMax M3 microplate reader (Molecular Devices).

### 2,3-Diaminonaphthalene (DAN) assay

To measure the potential ability of DBIC to scavenge NO, the indicated concentrations of DBIC were incubated with fresh GSNO (10 μM) for 20 min at RT. Samples were then incubated for 30 min after addition of 2,3-diaminonaphthalene (DAN, 100 μM). This assay detects S-nitrosothiols by conversion of DAN to the highly fluorescent compound 2,3-naphthotriazole (NAT). NAT was quantified at an excitation wavelength of 375 nm and emission of 450 nm using a spectrofluorometer (F2700, HITACHI). The fluorescence intensity curve of serial nitrite dilutions was used to construct a standard curve.

### RT-qPCR

A ReverTra Ace qPCR RT kit (TOYOBO) was used to synthesize complementary DNAs (cDNA) according to the manufacturer’s instructions. The qPCR was performed with KOD SYBR qPCR Mix (TOYOBO) under the following conditions: 98 °C for 2 min, followed by 40 cycles of 98 °C for 10 s, 60 °C for 10 s, 68 °C for 30 s. All qPCRs amplified single products, as confirmed by the melting curve and by electrophoresis using 2% agarose gels with 0.5 ng/ml ethidium bromide. The following primer sets were used: human *Ccnd2* 5′-GCG GAG AAG CTG TGC ATT TA-3′ and 5′-GCC AGG TTC CAC TTC AAC TTC-3′; human *Dnmt3b* 5′-GCC TCA AAC CCA ACA CG-3′ and 5′-AAT TTC CTA CTG CCT GCA CGA-3′; human *ACTB* 5′-TCA CCC ACA CTG TGC CCA TCT ACG A −3′ and 5′-CAG CGG AAC CGC TCA TTG CCA ATG G-3′; human *ALDOC* 5′-GGC GCT TAC CTT CTC CTA TGG-3′ and 5′-AAG CCC ATT CAC CTC AGC CC-3′; human CA9 5′-GAA ATC GCT GAG GAA GGC TC-3′ and 5′-CGG TGT AGT CAG AGA CCC CT-3′; human EGR1 5′-AGC ACC TGA CCG CAG AGT C-3′ and 5′-ACT GAC CAA GCT GAA GAG GG-3′; human GPR137C 5′-GGT CCT CTT TCT GTG GGA ACA-3′ and 5′-GCC AGC AGG TGC CAA ATT C-3′. The 2^−ΔΔC*t*^ relative quantification method, using ACTB for normalization, was used to estimate the target gene expression. Fold-change was calculated relative to mRNA expression levels in control samples.

### RNA-seq analysis

Total RNA was extracted from HeLa cells using the RNeasy Mini Kit (Qiagen, GmbH, Hilden, Germany). Quality control of RNA was strictly checked with a 2100 bioanalyzer (Agilent Technologies, Inc., Santa Clara, CA, USA). Whole-transcriptome RNA sequencing using the SureSelect strand-specific RNA library preparation kit (Agilent) was performed as previously described^51^. Libraries were pooled and paired-end sequenced (2 × 75) on an Illumina NextSeq 550 system. All sequence data files were analyzed with default parameters using the CLC genomics workbench (Qiagen, GmbH, Hilden, Germany). Quantitative analysis was calculated as transcripts per million. The sequence data files are deposited in the DNA Data Bank of Japan (DRA012332).

### Targeted methylation sequencing

HeLa cells were exposed to 200 µM SNOC and then incubated for the indicated times, or transduced with NOS2 for 48 h using Lipofectamine 3000. Subsequently, genomic DNA was isolated using the Wizard Genomic DNA Purification Kit (Promega). Genomic DNA (1 µg) was fragmented to a peak size of 155–170 bp with a Covaris S220 focused ultrasonicator (Covaris, Inc., Woburn, MA, USA). Targeted bisulfite sequencing libraries were prepared using the TruSeq Methyl Capture EPIC Library Prep kit (Illumina) according to the manufacturer’s protocol, followed by 101 bp paired-end sequencing on a NextSeq550 system (Illumina). Analysis of methylation sites was performed using MethylSeq v2.0.0, which employed Bismark for methyl calling and aligned to the reference human genome (hg19) using Bowtie2. The ratio of the number of sequenced methylated cytosine reads to the total number of reads for each locus was evaluated. We only considered cytosine sites in targeted regions of Illumina-optimized capture probes with at least ≥10 reads and were measured in every sample. The sequence data files have been deposited in the DNA Data Bank of Japan (https://ddbj.nig.ac.jp/resource/sra-submission/DRA012330).

### Bisulfite sequencing for the *Ccnd2* promoter region

Procedures were performed as previously described^52^. Genomic DNA from HeLa cells was extracted using the Wizard Genomic DNA Purification Kit (Promega) and bisulfite-converted with the Epitect Bisulfite Kits (QIAGEN), according to the manufacturer’s protocol. After bisulfite conversion, two different regions within the Ccnd2 promoter that are enriched in CpG sites were amplified by first-step PCR using the following primers: 5′-TTT GAT GTG TTT ATG TGT TTT TT-3′ and 5′-AAA ATA CCT CCC AAC CAA ATA ATT C-3′ (Target 1); 5′-TTT TTA GAT AAA TTG GGG AGG-3′ and 5′-TTT TAA ATT CCC CTA AAA CA-3′ (Target 2); 5′-CAG GAA ACA GCT ATG AC-3′ (M13 RV). PCR amplification was performed using EpiTaq HS (Takara), and the primer sets for Target 1 and 2 amplified products with a size of 290 and 399 bp, respectively. The PCR products were purified by QIAquick PCR Purification Kit (QIAGEN) and cloned into pMD20-T vector using the Mighty TA-cloning kit (Takara). At least 12 randomly selected clones from each culture dish were sequenced and quantified for methylation status by QUMA (http://quma.cdb.riken.jp/).

### Measurement of 5-methylcytosine levels

For the percentage of 5-methylcytosine in global DNA methylation in AGS cells, a MethylFlash Global DNA methylation (5-mc) ELISA Easy Kit (Epigentek) was used. Briefly, DNA samples (100 ng) were bound to high DNA affinity strip wells. Methylated DNA was detected using the capture and detection antibodies to 5-methyl cytosine, and then quantified colorimetrical by reading the absorbance at 450 nm using a model 680 microplate reader. The amount of methylated DNA was proportional to the optical density (OD) intensity measured. The absolute amount of methylated DNA was quantified as per protocol using a standard curve, plotting the OD values vs. 5 serial dilution of control methylated DNA (0.5−10 ng).

### Molecular modeling methodology

Comparative modeling of DNMT3B based on DNMT3A crystal structure (PDB code 2QRV) was accomplished using I-TASSER server^53,54^. Modeling the loop in the region of Thr773 to Asn786 was performed utilizing the kinematic loop modeling protocol implemented in Rosetta^55^. Ten thousand loop models were generated that were ranked by Rosetta ‘total score’. Clustering of the top scoring 100 loop models was then performed by the clustering utility in Rosetta^56^. SiteMap was employed to predict small-molecule binding pockets on the surface of DNMT3B models^57,58^.

To identify compounds interfering with the *S*-nitrosylation of DNMT3B without affecting enzymatic activity, structure-based virtual screening was carried out following a hierarchical docking protocol. Initially, the Namiki-Shoji collection (http://www.namiki-s.co.jp) of ~4 million commercially available compounds was docked to each of the three DNMT3B models employing the FRED docking program^59,60^. A conformational ensemble of compounds for FRED docking was generated using OMEGA^61,62^. A maximum of two hundred conformations per compound was generated. Docking was performed at the top scoring SiteMap pockets for all three DNMT3B models. A single pose per compound was generated, and all compounds were rank-ordered utilizing the Chemgauss4 scoring function. The top ranking 10,000 molecules for each DNMT3B model were then redocked using the Glide docking method^63–66^. Ligands for Glide docking were prepared using LigPrep^67^, which added hydrogens, assigned atomic charges using OPLS-2005 forcefield^68^, and generated the ionization and tautomeric state of the ligands. Schrodinger’s Maestro Protein Preparation Utility was employed for receptor preparation^69^. Grids for molecular docking were generated using SiteMap pockets. Molecular docking was performed using the standard precision (SP) and extra precision (EP) modes of Glide. Compounds were rank-ordered based on the Glide ‘docking score’, and the top ranking 200 compounds were selected for each model. All compounds were visually analyzed for the interactions they made with DNMT3B model, and 87 compounds were finally selected for purchase from commercial vendors for further experimental testing.

### Surface plasmon resonance analysis

Recombinant DNMT3B protein was purchased from Active Motif (Catalog No: 31413) and dialyzed against 10 mM acetate buffer (pH 4.4). DNMT3B protein was immobilized onto a CM5 sensor chip at a surface density of ~15,000 RU using an amine coupling kit (GE Healthcare; BR100050) according to the manufacturer’s protocol. A 3-fold dilution series (5 concentrations) of DBIC and its derivatives (DBIC-neg1 and DBIC-neg2) were prepared in running buffer (20 mM HEPES–NaOH, pH 7.4, 150 mM NaCl, 0.05% Surfactant P20, 0.1% DMSO) and flowed over a DNMT3B-immobilized surface at a flow rate of 30 µL/min in single-cycle mode using a BIAcore T200 instrument (GE Healthcare) at 25 °C. The results were evaluated using BIAcore T200 software v.3.0 (GE Healthcare).

### Pharmacological profiling to assess possible off-target effects

Assessment of potentially significant off-target effects of DBIC to major physiologically important targets was performed by DiscoverX (Eurofins) in 68 cell-based assays using the Safety47^TM^ Panel. Each assay in this panel was carried out with 10 μM DBIC, representing 100-fold the therapeutic dose used in efficacy assays.

### Neoplastic cell growth assay by cell counting

Approximately 1.0 × 10^4^ AGS cells were seeded in 12-well plates. After overnight culture, the medium was changed to DMEM supplemented with 2% FBS. The cells were pretreated with 100 nM DBIC or DBIC-neg for 1 h, and exposed to GSNO (10 μM). Every 24 h, reagents were re-added for a total of 72 h (the culture medium was completely replaced with fresh medium at 48 h). Cells were rinsed twice with PBS and fixed in 4% paraformaldehyde (Wako Pure Chemical Industry) in PBS for 30 min at 37 °C. Following fixation, cells were stained with Hoechst 33258 (10 μM diluted in PBS; Sigma-Aldrich) for 30 min at 37 °C. Cell counting was performed using a box-type epifluorescence microscope FX100 (Olympus), and the cell number was quantified with ImageJ software.

### 3D neoplastic cell aggregation assay

NOC-18 (NONOate) was dissolved in a 10 mM NaOH solution to prevent spontaneous release of NO and then diluted with cell culture medium to balance pH with HEPES buffer after addition of NO donors, which can otherwise lower the pH. 1 × 10^4^ FPCK-1-1 or QR-32 cells were plated in 6-well plate and incubated overnight. The attached cells were then repetitively treated with 126 µM NOC-18 every 3 days. Precise procedures were described elsewhere^38^.

FPCK-1-1 cells were filtered through a 20 µm cell strainer mesh (NRS-020, Nippon Rikagaku Kikai Co. Ltd, Tokyo) to obtain single cells, and were resuspended in 1.75% methylcellulose-containing medium. 1 × 10^3^ FPCK-1-1 cells were plated in 96-well ultra-low attachment plate. Sphere-formation was determined at a given day by counting cell aggregates with larger than 30 µm diameter using an inverted phase contrast microscopy (Keyence, Osaka, Japan).

The liquid overlay technique was used to generate spheroids^38^. SeaPlaque GTG agarose (50110, BioWhittaker, Rockland, ME) was diluted to 1% with serum-free medium, and 24-well plate was coated with a thin layer of this solution. 1 × 10^4^ QR-32 cells filtered through a 20 µm cell strainer mesh were then plated onto each solidified well. Under these conditions, they could not attach to the substrate (culture plate). For spheroid formation, the cells were agitated gently once a day for the initial 2 days.

### Inflammation-related carcinogenesis model

Procedures were performed as previously described^39,40^. In brief, mice were divided randomly into five groups for i.p. injection with PBS, vehicle, DBIC, DBIC-neg1, and 1400 W. After mice were anaesthetized, a small incision was made in the right flank of the pelvic region. A piece of gelatin sponge (10 × 5 × 3 mm; Spongel, Astellas Pharma) was inserted, and the wound was closed with clips. QR-32 cells (1 × 10^5^ cells/0.1 ml saline) were then immediately implanted into the pre-inserted sponge in the subcutaneous space of mice on day 0. PBS, vehicle, DBIC (25 mg/kg), DBIC-neg1 (25 mg/kg), or 1400 W (6 mg/kg) were administered i.p. in a volume of 200 μl once daily from pre-implantation day 2 through day 35. Body weight and tumor diameter were recorded twice weekly through day 35 after implantation. Tumor diameter was calculated by the following formula: Tumor diameter = (length + width)/2. Vehicle was composed of 15% DMSO, 17.5% cremophor EL (Wako Pure Chemical Industry), 8.75% ethanol, 8.75% HCO-40 (Wako Pure Chemical Industry), and 50% PBS.

### Pharmacokinetic (PK) analysis

Eight-week-old male Crl:CD1 (ICR) mice were used for pharmacokinetic studies. ICR mice were kept in a dark/light cycle with the light on at 7 a.m. and the light off at 7 p.m. under a temperature of 20–26 °C and humidity of 40–70%. PK studies were performed using male inflammation-related carcinogenesis (ICR) model mice at Nemoto Science Co., Ltd., following animal experimental protocol and procedures approved by the Institutional Animal Care and Use Committee. Plasma concentrations were measured at 0.25, 0.5, 1, 2, 4, 6, and 24 h following a single i.p. injection of 25 mg/kg DBIC. Tumor samples for measuring DBIC concentration were collected from ICR model mice 1 h after the last administration of drug. Compound concentrations in tumor tissues were measured by LC-MS/MS as previously reported^70^.

### Statistical analysis

Data are expressed as the mean ± s.e.m. Statistical comparisons were performed using two-tailed Student’s *t*-test or Fisher’s exact test. The chi-squared test (χ^2^-test) was performed to compare carcinogenic rate in mice. Multiple comparisons were performed using one-way (two-way) analysis of variance (ANOVA) with Bonferroni’s post hoc test, Tukey’s post hoc test, or Dunnett’s post hoc test using GraphPad Prism 8 (GraphPad Software, La Jolla, CA, USA). A *P* value <0.05 was considered to be significant.

### Chemical syntheses

Synthesis of Compound **2**: To a solution of 1*H*-benzo[*d*]imidazole-5-carboxylic acid (**1**, 1.00 g, 6.17 mmol) in EtOH (15 ml), conc. H_2_SO_4_ (0.5 ml) was added dropwise. The mixture was refluxed for 19.5 h. Ice water (170 ml) was added to the reaction solution and adjusted to pH 6 with NaHCO_3_. The solution was extracted by AcOEt (100 ml × 3), the organic layer was washed with brine, dried by MgSO_4_, and the solvent was removed to yield an oily product (1.05 g, 5.54 mmol), which was used without further purification. A mixture of the oily product (1.04 g, 5.33 mmol) and hydrazine hydrate (1.04 ml, 21.3 mmol) in EtOH (5 ml) was refluxed for 66.5 h. After cooling, the resulting crystals were filtered and washed with EtOH to give **2** (0.826 g, 4.69 mmol) in 76% yield, with purple sandiness, mp 253–257 ˚C; ^1^H-NMR (400 MHz, DMSO-*d*_6_) δ: 4.56 (2H, s), 7.68 (1H, d, *J* = 8.4 Hz), 7.79 (1H, dd, *J* = 8.4 Hz), 8.18 (1H, s), 8.40 (1H, s), 9.83 (1H, s), 8.34 (1H, s), 12.69 (1H, s).

Synthesis of Compound **3** (**DBIC**): A mixture of **2** (0.10 g, 0.57 mmol) and 3,4-dihydroxybenzaldehyde (0.79 ml, 0.57 mmol) in DMF (3 ml) was heated at 100 ˚C for 21.5 h. After cooling, water (300 ml) was added to the mixture. The resulting crystals were filtered and washed with water and EtOH to give **4** (0.154 g, 0.52 mmol) in 91% yield, with colorless sandiness, mp 286–290 ˚C; ^1^H-NMR (400 MHz, DMSO-*d*_6_) δ: 6.84 (1H, d, *J* = 8.0 Hz), 6.98 (1H, dd, *J* = 8.0 Hz), 7.30 (1H, d, *J* = 1.2 Hz), 7.72 (1H, d, *J* = 8.4 Hz), 7.84 (1H, d, *J* = 8.4 Hz), 8.27 (1H, s), 8.34 (1H, s), 8.42 (1H, s), 9.34 (1H, s), 9.44 (1H, s), 11.67 (1H, s), 12.80 (1H, s); analysis (% calcd, % found for C_15_H_12_N_4_O_3_·1/2 H_2_O: C (59.01, 59.14), H (4.29, 4.18), N (18.35, 18.19); ^13^C-NMR (400 MHz, DMSO-*d*_*6*_, ppm) δ: 113.88, 113.91, 115.29, 115.43, 116.67, 121.58, 126.51, 126.64, 131.53, 132.21, 133.65, 143.27, 143.28, 146.73, 149.11, 149.94, 162.94.

Synthesis of Compound **4** (**DBIC-neg1**): A mixture of **2** (0.10 g, 0.57 mmol) and benzaldehyde (0.69 ml, 0.68 mmol) in DMF (3 ml) was heated at 100 ˚C for 4.5 h. After cooling, water (200 ml) was added to the mixture. The resulting crystals were filtered and washed with water and EtOH to produce **4** (0.127 g, 0.48 mmol) in 84% yield, with colorless sandiness, mp 298–301 ˚C; ^1^H-NMR (400 MHz, DMSO-*d*_6_) δ: 7.50–7.52 (3H, m), 7.78–7.88 (4H, m), 8.31 (1H, brs), 8.44 (1H, s), 8.54 (1H, s), 11.93 (1H, s), 12.82 (1H, s); analysis (% calcd, % found for C_15_H_12_N_4_O): C (68.17, 67.90), H (4.58, 4.47), N (21.20, 21.05); ^13^C-NMR (400 MHz, DMSO-*d*_*6*_, ppm) δ: 115.53, 115.83, 122.07, 122.81, 127.94, 129.75, 130.86, 135.42, 139.07, 140.52, 144.70, 148.17, 164.57.

Synthesis of Compound **6** (**DBIC-neg2**): To a solution of acetohydrazide (**5**, 0.200 g, 2.70 mmol) and 3,4-dihydroxybenzaldehyde (0.373 mg, 2.70 mmol) in EtOH (5 mL) was added. The mixture was stirred at reflux for 2 h. After cooling, the resulting crystals were filtered to give the *E*,*Z* mixture (3:2) **6** (0.378 g, 1.95 mmol) in 72% yield, mp 208─217 ˚C; ^1^H-NMR (400 MHz, DMSO-*d*_6_) *E*-form; δ: 1.89─2.14 (3H, m), 6.74 (1H, d, J = 8.0 Hz), 6.83─6.87 (1H, m), 7.10 (1H, d, J = 2.0 Hz), 7.78 (1H, s), 9.17 (1H), 9.32 (1H), 10.98─11.09 (1H, m). *Z*-form; δ: 1.89─2.14 (3H, m), 6.74 (1H, d, *J* = 8.0 Hz), 6.83─6.87 (1H, m), 7.15 (1H, d, *J* = 2.0 Hz), 7.92 (1H, s), 9.17 (1H), 9.32 (1H), 10.98─11.09 (1H, m); analysis (% calcd, % found for C_9_H_10_N_2_O_3_): C (55.67, 55.54), H (5.19, 5.14), N (14.43, 14.38); ^13^C-NMR (400 MHz, DMSO-*d*_*6*_, ppm) *E*-form δ: 20.77, 112.96, 116.11, 120.41, 126.29, 143.70, 146.20, 148.07, 172.07; *Z*-form δ: 22.15, 113.13, 116.04, 120.91, 126.32, 143.70, 146.63, 148.28, 165.72.

Synthesis of Compound **7** (**DBIC-der1**): To a solution of 1*H*-benzo[*d*]imidazole-5-carboxylic acid (**1**, 324.3 mg, 2.0 mmol), 2-(3, 4-dihydroxyphenyl)-ethylamine hydrochloride (379.3 mg, 2.0 mmol), 1-(3-dimethylaminopropyl)-3-ethylcarbodiimide (421.7 mg, 2.2 mmol), and 4-dimethylaminopyridine (268.8 mg, 2.2 mmol) in DMF (10 mL) was added. The mixture was stirred at RT for 48 h. The reaction solution was then purified by silica gel column chromatography (CHCl_3_/MeOH, 5:1) to gain **7** (34.1 mg, 0.12 mmol) in 5.7% yield, colorless sandiness; mp 210─218 °C; ^1^H-NMR (400 MHz, DMSO-*d*_6_, ppm) δ: 2.71 (2H, t, *J* = 7.6 Hz), 3.45 (2H, q, *J* = 7.6 Hz), 6.53 (1H, dd, *J* = 8.0 Hz), 6.68–6.70 (2H, m), 7.59─7.80 (2H, m), 8.08─8.39 (2H, m), 8.55 (1H, brs), 8.70 (1H, s), 8.83 (1H, s), 12.69─12.77 (1H, m); HRMS (ESI) *m*/*z*: Calcd for C_24_H_15_N_3_NaO_3_: 320.1011; Found: 320.1006 [M^+^Na]^+^; ^13^C-NMR (100 MHz, DMSO-*d*_6_, ppm) δ: 32.90, 40.87, 115.30, 116.32, 116.59, 119.75, 121.68, 127.58, 128.50, 144.29, 144.56, 145.80, 167.17.

### Reporting summary

Further information on research design is available in the Nature Portfolio Reporting Summary linked to this article.

## Supplementary information

Supplementary Information

Description of additional Supplementary File

Supplementary Data1

Reporting Summary

## Data Availability

The data that support this study are available from the corresponding authors upon reasonable request. All data analyzed by targeted DNA methylation sequencing and RNA-seq were deposited in the DNA Data Bank of Japan under the accession number DRA012230 and DRA012332, respectively. The mass spectrometry data have been deposited to the ProteomeXchange Consortium via the PRIDE^71^ partner repository with the dataset identifier PXD039437. Source data are provided with this paper.

## Source data

Source Data
