# Supplementary material for: Pivotal role for S-nitrosylation of DNA methyltransferase 3B in epigenetic regulation of tumorigenesis
Source: Nat Commun. 2023 Feb 4;14:621. doi: 10.1038/s41467-023-36232-6 (PMC9899281; doi:10.1038/s41467-023-36232-6)
Supplement: Supplementary file 1 — Supplementary Information [file 41467_2023_36232_MOESM1_ESM.pdf]

# Supplementary Figures and Legends

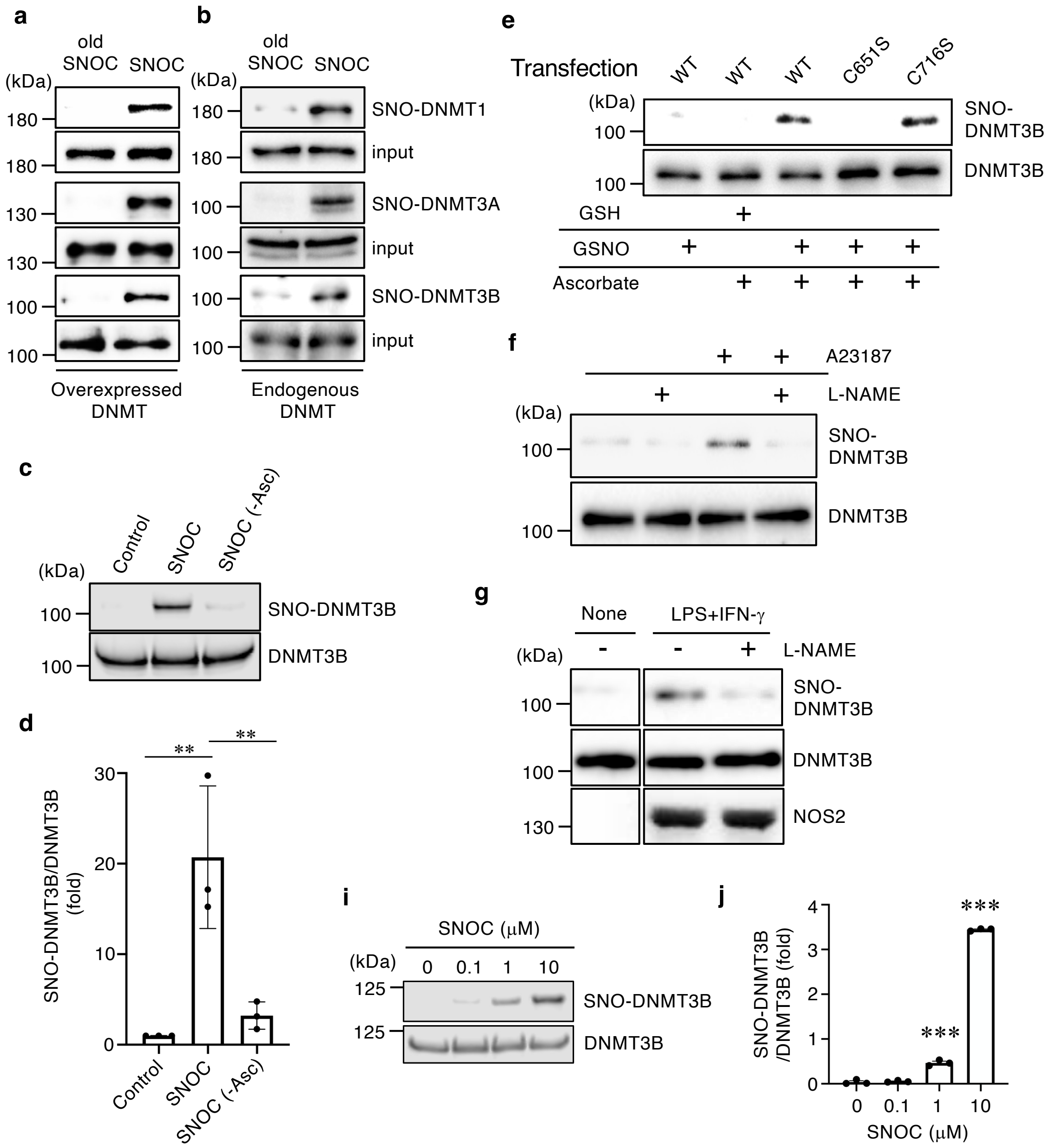


h

| Patient Information | | | | | |
|---|---|---|---|---|---|
| **Sample** | **Age** | **Sex** | **Tissue** | **%Tumor** | **Stage** |
| Normal #1 | 71 | Female | Normal colon | 0 | Not applicable |
| Normal #2 | 40 | Male | Normal colon | 0 | Not applicable |
| Normal #3 | 40 | Female | Normal colon | 0 | Not applicable |
| Normal #4 | 52 | Female | Normal colon | 0 | Not applicable |
| Normal #5 | 75 | Male | Normal colon | 0 | Not applicable |
| Normal #6* | 68 | Male | Normal colon | 0 | Not applicable |
| | | | | | |
| Tumor #1 | 81 | Female | Colorectal cancer | 70 | T4 |
| Tumor #2 | 62 | Female | Colorectal cancer | 90 | T3 |
| Tumor #3 | 78 | Male | Colorectal cancer | 90 | T2 |
| Tumor #4 | 57 | Female | Colorectal cancer | 70 | T3 |
| Tumor #5 | 76 | Female | Colorectal cancer | 70 | T3 |
| Tumor #6* | 68 | Male | Colorectal cancer | 70 | T3 |

*Normal #6 and Tumor #6 are from the same patient

**Supplementary Figure 1 | *S*-Nitrosylation of DNMT. a**, SNO-DNMT formation after exposure to an NO donor. HEK293 cells transfected with GFP-tagged DNMT1, GFP-tagged DNMT3A, or FLAG-tagged DNMT3B were exposed to 100 μM SNOC. After 1 hr, SNO-DNMTs were detected by biotin-switch assay. 'Old' SNOC, from which NO had been dissipated, was used as a control. **b**, *S*-Nitrosylation of endogenous DNMT. Formation of SNO-DNMT1 and SNO-DNMT3A in HEK cells, and SNO-DNMT3B in AGS cells after exposure to 100 μM SNOC. **c,** *S*-Nitrosylation of full-length DNMT3B. Recombinant DNMT3B (1 μg) was incubated in 100 μM SNOC and analyzed 30 min later by biotin-switch assay using anti-DNMT3B antibody in the absence or presence of ascorbate. **d,** Ratio of SNO-DNMT3B/total DNMT3B quantified by densitometry. Values are mean ± s.e.m. ($n$ = 3; $^{**}P < 0.01$ one-way ANOVA with Tukey's *post-ho*c test). **e,** HEK293 cells, transduced with WT or C-to-S FLAG-tagged DNMT3B mutant constructs, were exposed to 100 μM GSNO or GSH as a control. After 1 hr, SNO-DNMT3B was detected using anti-FLAG antibody by biotin-switch assay in the absence or presence of ascorbate. *Top panels*: SNO-DNMT3B. *Bottom panels*: Total DNMT3B. **f**, NOS1 activation increased endogenous SNO-DNMT3B. *Top panels*: HEK-293 cells stably expressing NOS1 were assayed for endogenous SNO-DNMT3B. NOS1 was activated by calcium ionophore A23187 (5 μM) in the presence or absence of NOS inhibitor ($N^{G}$-nitro-L-arginine methyl ester; L-NAME). *Bottom panels*: DNMT3B. **g**, RAW 264.7 cells were exposed to 10 μg/ml lipopolysaccharide (LPS) plus 100 units/ml interferon-γ (IFN-γ) for 24 hr to induce NOS2 in the absence or presence of the NOS inhibitor, $N^{G}$-nitro-L-arginine methyl ester (L-NAME). SNO-DNMT3B formation was detected 24 hr after treatment. *Top Panels*: SNO-DNMT3B. *Middle panels*: Total DNMT3B. *Bottom panels*: Total NOS2. **h**, List of human subjects providing control (normal) and colon cancer tissue for this study. **i,** *In vitro* SNO-DNMT3B formation by low concentrations of SNOC. **j,** Ratio of SNO-DNMT3B/total DNMT3B quantified by densitometry. Values are mean ± s.e.m. ($n$ = 3; $^{***}P < 0.001$ vs. SNOC 0 μM by one-way ANOVA with Dunnett's *post-hoc* test). Source data are provided as a Source Data file.

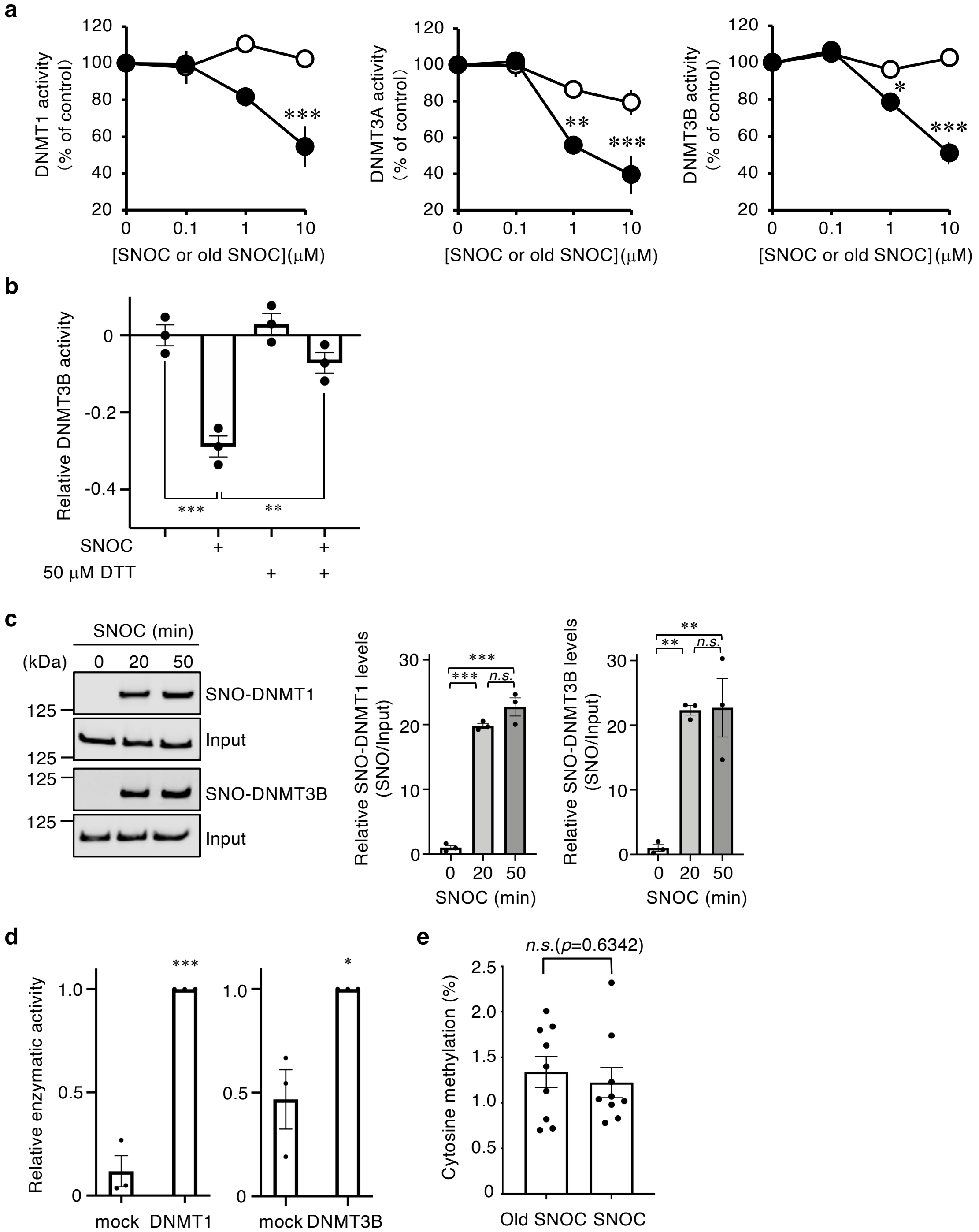


**Supplementary Figure 2 | Effects of NO on DNMT3B-mediated gene expression. a**, Effect of *S*-nitrosylation on DNMT activity. Each recombinant DNMT was incubated with the indicated concentrations of old SNOC (open circles) or SNOC (closed circles). After 1 hr, DNMT enzymatic activity was assayed with the DNMT Direct Activity Assay Kit. Values

are mean ± s.e.m. (*n* = 3; *$P$ < 0.05, **$P$ < 0.01, ***$P$ < 0.001 vs. SNOC 0 μM by one-way ANOVA with Dunnett's *post-hoc* test). **b,** DTT reversed the inhibition of enzymatic activity induced by NO exposure of recombinant DNMT3B. Values are mean ± s.e.m. shown relative to basal activity (*n* = 3; **$P$ < 0.01, ***$P$ < 0.001, by one-way ANOVA with Bonferroni's *post-ho*c test). **c,** Differential temporal levels of *S*-nitrosylation of DNMT1 and DNMT3B *in vitro* assays using recombinant enzymes. Values are expressed as mean ± s.e.m. (*n* = 3; *$P$ < 0.05; **$P$ < 0.01; ***$P$ < 0.001 by one-way ANOVA with Tukey's *post-hoc* test). **d,** Relative DNMT activity in HEK293T cells transfected with DNMT1 or DNMT3B. DNMT enzymatic activity was determined using nuclear extracts of HEK293T cells expressing DNMT1 or DNMT3B. Data are mean ± s.e.m. (*n* = 3; *$P$ < 0.05, ***$P$ < 0.001 by two-tailed Student's *t*-test). **e,** Quantification of global DNA methylation in AGS cells. Values are expressed as mean ± s.e.m. Statistical significance was determined by two-tailed Student's *t*-test (*n* = 9; ns: not significant). Source data are provided as a Source Data file.

**a**

| Gene symbol | Description | Fold change | P-value |
|---|---|---|---|
| Upregulation (Fold change >1.5, P-value <0.05) | | | |
| AC004223.3 | - | 84.4 | 1.E-02 |
| AC004687.2 | - | 9.6 | 6.E-03 |
| AC004832.3 | - | 4.2 | 2.E-02 |
| AC005520.1 | - | 2.1 | 4.E-02 |
| AC009690.1 | - | 3.7 | 4.E-02 |
| AC011452.2 | - | 51.6 | 5.E-02 |
| AC012184.2 | - | 3.0 | 9.E-05 |
| AC012531.3 | - | 10.2 | 4.E-02 |
| AC068896.1 | - | 5.7 | 2.E-04 |
| AC099489.1 | - | 3.4 | 2.E-02 |
| AC126283.2 | - | 7.2 | 2.E-02 |
| AC135178.3 | - | 5.3 | 3.E-03 |
| AC138696.1 | - | 3.9 | 5.E-02 |
| AC138969.1 | - | 1.9 | 8.E-06 |
| ADORA2A | adenosine A2a receptor | 8.6 | 4.E-04 |
| AL109811.3 | - | 1.8 | 3.E-02 |
| AL133500.1 | - | 3.4 | 5.E-02 |
| AL136295.5 | - | 1.6 | 3.E-04 |
| AL162231.3 | - | 66.0 | 2.E-02 |
| ALDOC | aldolase, fructose-bisphosphate C | 1.5 | 2.E-04 |
| AQP7 | aquaporin-7 | 3.0 | 2.E-02 |
| ARHGAP11B | Rho GTPase activating protein 11B | 1.7 | 4.E-02 |
| ATP6V1G2-DDX39B | ATP6V1G2-DDX39B readthrough (NMD Candidate) | 2.1 | 3.E-05 |
| BIVM-ERCC5 | BIVM-ERCC5 readthrough | 1.6 | 3.E-03 |
| BOC | BOC cell adhesion associated, oncogene regulated | 6.6 | 3.E-02 |
| BX470111.1 | - | 2.8 | 5.E-02 |
| C3orf36 | chromosome 3 putative open reading frame 36 | 4.4 | 4.E-02 |
| C8orf44-SGK3 | C8orf44-SGK3 readthrough | 10.4 | 5.E-03 |
| CA9 | carbonic anhydrase 9 | 4.7 | 1.E-10 |
| CCDC153 | coiled-coil domain containing 153 | 10.3 | 3.E-02 |
| CCNI2 | cyclin I family member 2 | 38.9 | 5.E-02 |
| CDRT4 | CMT1A duplicated region transcript 4 | 2.0 | 3.E-03 |
| CFAP206 | cilia and flagella associated protein 206 | 5.8 | 5.E-02 |
| CHRM1 | cholinergic receptor muscarinic 1 | 3.0 | 4.E-02 |
| CLDN7 | claudin 7 | 4.8 | 2.E-02 |
| CSPG5 | chondroitin sulfate proteoglycan 5 | 1.7 | 2.E-02 |
| CTAGE15 | CTAGE family member 15 | 55.8 | 3.E-02 |
| CXCL10 | C-X-C motif chemokine ligand 10 | 3.4 | 5.E-02 |
| CXCL8 | C-X-C motif chemokine ligand 8 | 1.6 | 2.E-05 |
| CYP1A1 | cytochrome P450 family 1 subfamily A member 1 | 2.8 | 5.E-02 |
| EGR1 | early growth response 1 | 1.7 | 4.E-09 |
| EVA1B | eva-1 homolog B | 1.8 | 5.E-02 |
| FAM220A | family with sequence similarity 220 member A | 4.3 | 3.E-04 |
| FAM243A | family with sequence similarity 243 member A | 103.3 | 1.E-02 |
| FIBIN | fin bud initiation factor homolog | 1.6 | 4.E-02 |
| FOXD4 | forkhead box D4 | 2.7 | 2.E-02 |
| GNG3 | G protein subunit gamma 3 | 70.7 | 2.E-02 |
| GPR137C | G protein-coupled receptor 137C | 1.7 | 4.E-05 |
| GRPR | gastrin releasing peptide receptor | 3.1 | 4.E-02 |
| H2AB3 | H2A.B variant histone 3 | 38.9 | 5.E-02 |
| HSH2D | hematopoietic SH2 domain containing | 4.3 | 4.E-02 |
| IL24 | interleukin 24 | 3.3 | 2.E-02 |
| ITGB3 | integrin subunit beta 3 | 3.5 | 2.E-02 |
| ITGBL1 | integrin subunit beta like 1 | 2.6 | 4.E-02 |
| KCNK2 | potassium two pore domain channel subfamily K member 2 | 1.8 | 1.E-02 |
| KCNMB3 | potassium calcium-activated channel subfamily M regulatory beta subunit 3 | 1.5 | 1.E-02 |
| KCNT2 | potassium sodium-activated channel subfamily T member 2 | 2.5 | 1.E-02 |
| KRTCAP3 | keratinocyte associated protein 3 | 43.6 | 4.E-02 |
| LRRC56 | leucine rich repeat containing 56 | 1.7 | 2.E-02 |
| MAP6D1 | MAP6 domain containing 1 | 3.1 | 3.E-02 |
| MS4A7 | membrane spanning 4-domains A7 | 3.2 | 4.E-02 |
| MUC2 | mucin 2, oligomeric mucus/gel-forming | 4.2 | 2.E-02 |
| MYEF2 | myelin expression factor 2 | 3.0 | 3.E-02 |
| NAALADL2 | N-acetylated alpha-linked acidic dipeptidase like 2 | 2.1 | 5.E-02 |
| NMNAT2 | nicotinamide nucleotide adenylyltransferase 2 | 1.8 | 5.E-02 |
| NPIPA7 | nuclear pore complex interacting protein family member A7 | 1.8 | 3.E-05 |
| P2RX5-TAX1BP3 | P2RX5-TAX1BP3 readthrough (NMD Candidate) | 1.7 | 5.E-02 |
| PCDHB11 | protocadherin beta 11 | 2.9 | 4.E-02 |
| PLIN4 | perilipin 4 | 3.1 | 8.E-03 |
| PLSCR2 | phospholipid scramblase 2 | 42.0 | 4.E-02 |
| PPP2R2C | protein phosphatase 2 regulatory subunit Bgamma | 4.6 | 1.E-02 |
| PRSS16 | serine protease 16 | 3.0 | 4.E-02 |
| RAB11FIP4 | RAB11 family interacting protein 4 | 7.3 | 1.E-03 |
| RGPD5 | RANBP2 like and GRIP domain containing 5 | 11.5 | 1.E-06 |
| RNASEK-C17orf49 | RNASEK-C17orf49 readthrough | 2.9 | 3.E-05 |
| SLC34A3 | solute carrier family 34 member 3 | 1.9 | 2.E-02 |
| SLITRK6 | SLIT and NTRK like family member 6 | 1.6 | 4.E-02 |
| SMIM11B | small integral membrane protein 11B | 3.0 | 4.E-04 |
| TBC1D3H | TBC1 domain family member 3H | 40.7 | 5.E-02 |
| TFAP2E | transcription factor AP-2 epsilon | 2.4 | 5.E-02 |
| TLCD4-RWDD3 | TLCD4-RWDD3 readthrough | 7.9 | 1.E-05 |
| TLDC2 | TBC/LysM-associated domain containing 2 | 84.2 | 1.E-02 |
| TMEM139 | transmembrane protein 139 | 1.6 | 2.E-02 |
| TMEM74 | transmembrane protein 74 | 1.6 | 5.E-02 |
| ZNF559-ZNF177 | ZNF559-ZNF177 readthrough | 3.6 | 8.E-03 |
| ZNF816-ZNF321P | ZNF816-ZNF321P readthrough | 1.7 | 2.E-02 |

**b**

| Gene symbol | Description | Fold change | P-value |
|---|---|---|---|
| Downregulation (Fold change <-1.5, P-value <0.05) | | | |
| AC001226.2 | - | -1.7 | 4.E-02 |
| AC002310.4 | - | -107.6 | 6.E-03 |
| AC002996.1 | - | -1.9 | 2.E-02 |
| AC005324.4 | - | -40.7 | 3.E-02 |
| AC005702.1 | - | -30.9 | 5.E-02 |
| AC006538.2 | - | -2.0 | 2.E-02 |
| AC009412.1 | - | -3.7 | 1.E-04 |
| AC010323.1 | - | -1.5 | 3.E-02 |
| AC010531.1 | - | -67.9 | 1.E-02 |
| AC010547.4 | - | -123.1 | 6.E-03 |
| AC020922.1 | - | -43.6 | 2.E-02 |
| AC026954.2 | - | -2.1 | 3.E-03 |
| AC037459.1 | - | -89.2 | 6.E-03 |
| AC078927.1 | - | -146.8 | 2.E-03 |
| AC079594.2 | - | -736.1 | 3.E-05 |
| AC091057.6 | - | -1.6 | 3.E-03 |
| AC091167.2 | - | -3.8 | 2.E-03 |
| AC092718.8 | - | -1.8 | 2.E-02 |
| AC097634.4 | - | -173.8 | 3.E-03 |
| AC104109.3 | - | -2.0 | 1.E-02 |
| AC114490.3 | - | -4.9 | 8.E-04 |
| AC118281.1 | - | -2.0 | 3.E-02 |
| AC131160.1 | - | -6.0 | 8.E-03 |
| AD000671.1 | - | -2.8 | 5.E-02 |
| ADM2 | adrenomedullin 2 | -1.6 | 5.E-02 |
| AL035078.4 | - | -308.1 | 4.E-04 |
| AL353588.1 | - | -3.5 | 5.E-02 |
| AL359736.1 | - | -98.6 | 1.E-02 |
| AL359922.1 | - | -1.6 | 3.E-02 |
| AL445685.3 | - | -6.8 | 2.E-02 |
| AL451062.3 | - | -82.7 | 1.E-02 |
| AL513165.2 | - | -52.7 | 2.E-02 |
| AL691442.2 | - | -42.2 | 5.E-02 |
| AOAH | acyloxyacyl hydrolase | -9.3 | 4.E-02 |
| AP002373.1 | - | -243.0 | 7.E-04 |
| AP3B2 | adaptor related protein complex 3 subunit beta 2 | -13.1 | 3.E-02 |
| APCDD1L | APC down-regulated 1 like | -7.5 | 3.E-02 |
| ARL2-SNX15 | ARL2-SNX15 readthrough (NMD Candidate) | -245.0 | 1.E-03 |
| CARD11 | caspase recruitment domain family member 11 | -1.8 | 3.E-02 |
| CCDC103 | coiled-coil domain containing 103 | -2.0 | 1.E-02 |
| CELF6 | CUGBP Elav-like family member 6 | -3.8 | 3.E-02 |
| CFAP61 | cilia and flagella associated protein 61 | -33.7 | 4.E-02 |
| CIDEB | cell death inducing DFFA like effector b | -89.3 | 2.E-02 |
| CNTNAP3C | contactin associated protein family member 3C | -4.1 | 4.E-02 |
| COMP | cartilage oligomeric matrix protein | -7.9 | 2.E-02 |
| CTSV | cathepsin V | -1.7 | 5.E-02 |
| CXCR5 | C-X-C motif chemokine receptor 5 | -41.9 | 4.E-02 |
| DNAAF8 | dynein axonemal assembly factor 8 | -3.4 | 7.E-03 |
| DNAJC25-GNG10 | DNAJC25-GNG10 readthrough | -1.7 | 5.E-04 |
| EYA2 | EYA transcriptional coactivator and phosphatase 2 | -3.0 | 5.E-02 |
| FNDC5 | fibronectin type III domain containing 5 | -3.7 | 4.E-02 |
| H2AB1 | H2A.B variant histone 1 | -33.7 | 4.E-02 |
| H2AC7 | H2A clustered histone 7 | -170.1 | 1.E-03 |
| H3C8 | H3 clustered histone 8 | -2.0 | 3.E-02 |
| H4C11 | H4 clustered histone 11 | -77.0 | 1.E-02 |
| HUS1B | HUS1 checkpoint clamp component B | -53.7 | 3.E-02 |
| INMT-MINDY4 | INMT-MINDY4 readthrough (NMD Candidate) | -6.8 | 1.E-02 |
| KIF12 | kinesin family member 12 | -31.0 | 4.E-02 |
| KLK14 | kallikrein related peptidase 14 | -4.9 | 4.E-02 |
| LRATD2 | LRAT domain containing 2 | -4.1 | 1.E-02 |
| LRRC29 | leucine rich repeat containing 29 | -7.8 | 2.E-02 |
| MEF2B | myocyte enhancer factor 2B | -3.2 | 2.E-02 |
| NKX2-5 | NK2 homeobox 5 | -28.4 | 5.E-02 |
| NPIPA8 | nuclear pore complex interacting protein family member A8 | -2.4 | 5.E-02 |
| NPIPA9 | nuclear pore complex interacting protein family, member A9 | -1.7 | 3.E-05 |
| OIT3 | oncoprotein induced transcript 3 | -7.5 | 3.E-02 |
| PIF1 | PIF1 5'-to-3' DNA helicase | -1.9 | 5.E-15 |
| PTGDS | prostaglandin D2 synthase | -2.5 | 4.E-02 |
| RIMBP3C | RIMS binding protein 3C | -6.0 | 4.E-03 |
| RXFP4 | relaxin family peptide/INSL5 receptor 4 | -4.7 | 4.E-02 |
| SLC16A11 | solute carrier family 16 member 11 | -43.6 | 3.E-02 |
| SNURF | SNRPN upstream open reading frame | -1.8 | 2.E-02 |
| SPDYE9 | speedy/RINGO cell cycle regulator family member E9 | -5.0 | 5.E-02 |
| SPECC1L-ADORA2A | SPECC1L-ADORA2A readthrough (NMD Candidate) | -69.7 | 2.E-02 |
| SRPX | sushi repeat containing protein X-linked | -3.1 | 4.E-02 |
| STX16-NPEPL1 | STX16-NPEPL1 readthrough (NMD Candidate) | -2.5 | 3.E-03 |
| SYNJ2BP-COX16 | SYNJ2BP-COX16 readthrough | -1.8 | 7.E-05 |
| TBC1D3D | TBC1 domain family member 3D | -1.6 | 9.E-03 |
| TBCEL-TECTA | TBCEL-TECTA readthrough | -7.0 | 7.E-03 |
| TNFAIP8L1 | TNF alpha induced protein 8 like 1 | -1.7 | 4.E-07 |
| TNFRSF6B | TNF receptor superfamily member 6b | -2.1 | 4.E-02 |
| TRIM6-TRIM34 | TRIM6-TRIM34 readthrough | -39.7 | 4.E-05 |
| TSPAN7 | tetraspanin 7 | -32.6 | 5.E-02 |
| UBE2F-SCLY | UBE2F-SCLY readthrough (NMD Candidate) | -6.5 | 4.E-04 |
| UGT1A9 | UDP glucuronosyltransferase family 1 member A9 | -33.5 | 5.E-02 |
| UTP14C | UTP14C small subunit processome component | -309.0 | 9.E-04 |
| ZNF177 | zinc finger protein 177 | -3.1 | 1.E-02 |

**c**

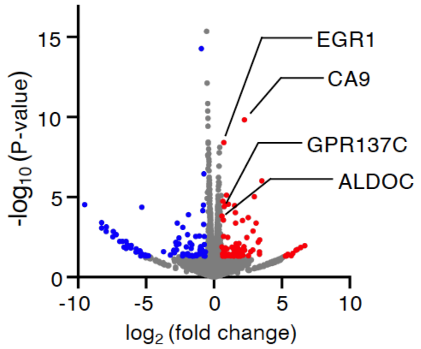


**d**

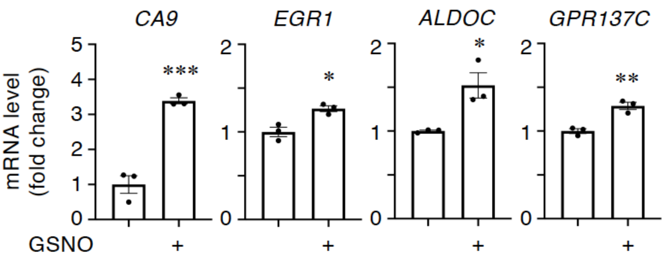

**Supplementary Figure 3 | NO-induced gene expression. a,b,** List of up-regulated (**a**) and down-regulated (**b**) genes after exposure to a NO donor. HeLa cells were exposed to 100 μM GSNO or GSH (as a control) for 24 hr. After RNA extraction, we performed whole-transcriptome RNA sequencing using the SureSelect strand-specific RNA Library Preparation Kit (Agilent) ($n$ = 3). Statistical values of differential expression were calculated with CLC Genomics Workbench (Qiagen) by Wald test. Genes with $P < 0.05$ and a fold-change of absolute value > 1.5 were considered significant. **c**, RNA-seq results shown in volcano plot displaying significantly differentially expressed genes (DEGs) after GSNO exposure compared to control GSH. Upregulated, downregulated, and unaffected genes are highlighted in red, blue, and gray, respectively. **d**, Gene expression upregulated after NO exposure. HeLa cells were incubated in 100 μM GSNO or GSH, and, after 24 hr, RT-qPCR was performed using specific primers for each mRNA. Values are expressed as mean ± s.e.m. ($n$ = 3; *$P < 0.05$; **$P < 0.01$; ***$P < 0.001$ by two-tailed Student's $t$-test). Source data are provided as a Source Data file.

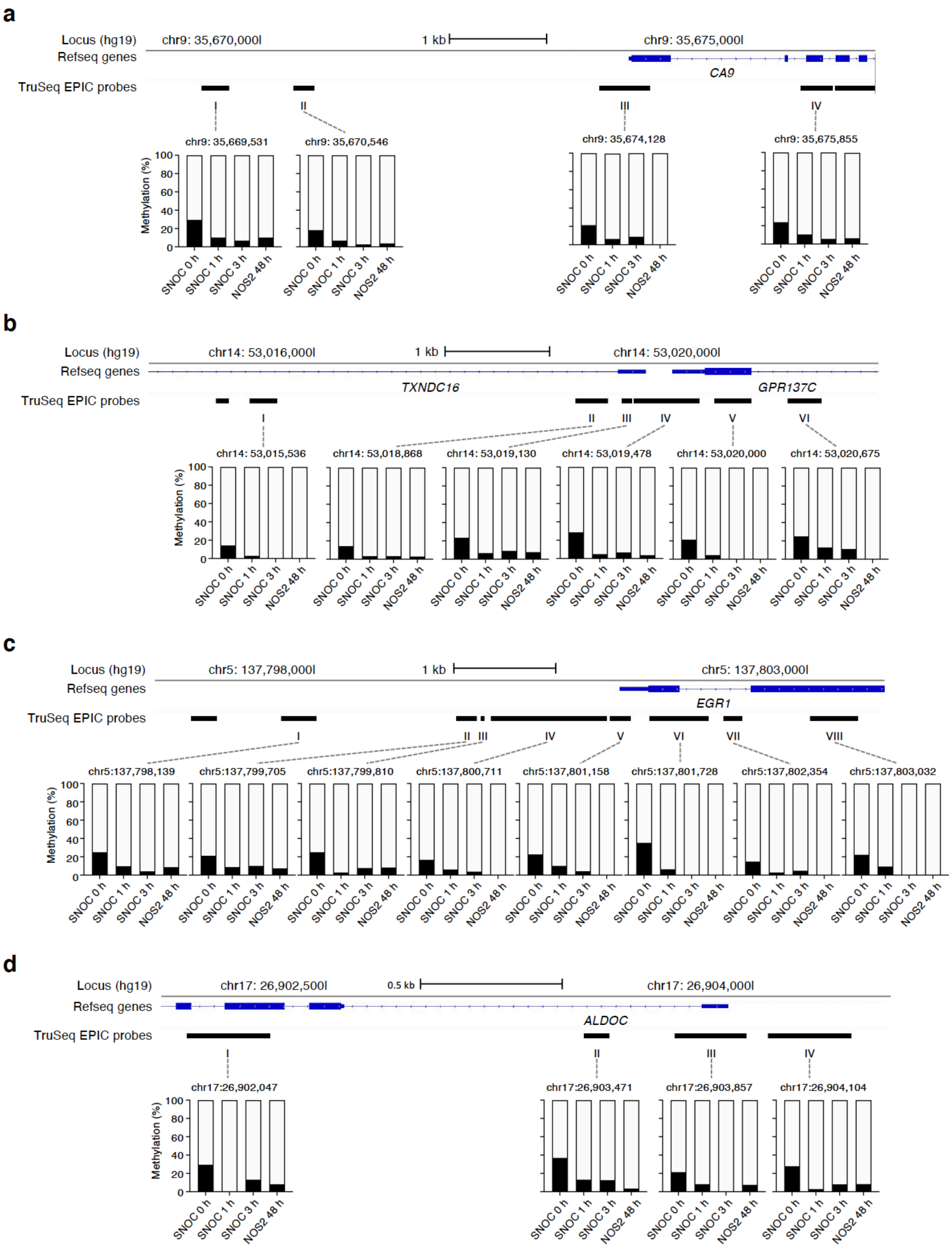
a
Locus (hg19)
chr9: 35,670,000
1 kb
chr9: 35,675,000
Refseq genes
CA9
TruSeq EPIC probes
I
II
III
IV
chr9: 35,669,531
chr9: 35,670,546
chr9: 35,674,128
chr9: 35,675,855
Methylation (%)
SNOC 0 h
SNOC 1 h
SNOC 3 h
NOS2 48 h
b
Locus (hg19)
chr14: 53,016,000
1 kb
chr14: 53,020,000
Refseq genes
TXNDC16
GPR137C
TruSeq EPIC probes
chr14: 53,015,536
chr14: 53,018,868
chr14: 53,019,130
chr14: 53,019,478
chr14: 53,020,000
chr14: 53,020,675
c
Locus (hg19)
chr5: 137,798,000
1 kb
chr5: 137,803,000
Refseq genes
EGR1
TruSeq EPIC probes
chr5:137,798,139
chr5:137,799,705
chr5:137,799,810
chr5:137,800,711
chr5:137,801,158
chr5:137,801,728
chr5:137,802,354
chr5:137,803,032
d
Locus (hg19)
chr17: 26,902,500
0.5 kb
chr17: 26,904,000
Refseq genes
ALDOC
TruSeq EPIC probes
chr17:26,902,047
chr17:26,903,471
chr17:26,903,857
chr17:26,904,104

**Supplementary Figure 4 | Effect of SNO on DNA methylation in promoter regions of selected gene loci.** Genome browser representation of genes significantly upregulated after SNOC exposure. Black boxes show regions targeted by Illumina-optimized capture probes (TruSeq EPIC probes). HeLa cells were exposed to SNOC for up to 3 h or transduced with NOS2 for 48 hr. Graphs show percentage of methylated cytosines at a representative site in each targeted region. **a**, *CA9*; **b**, *GPR137C*, **c**, *EGR1*; **d**, *ALDOC*. Source data are provided as a Source Data file.

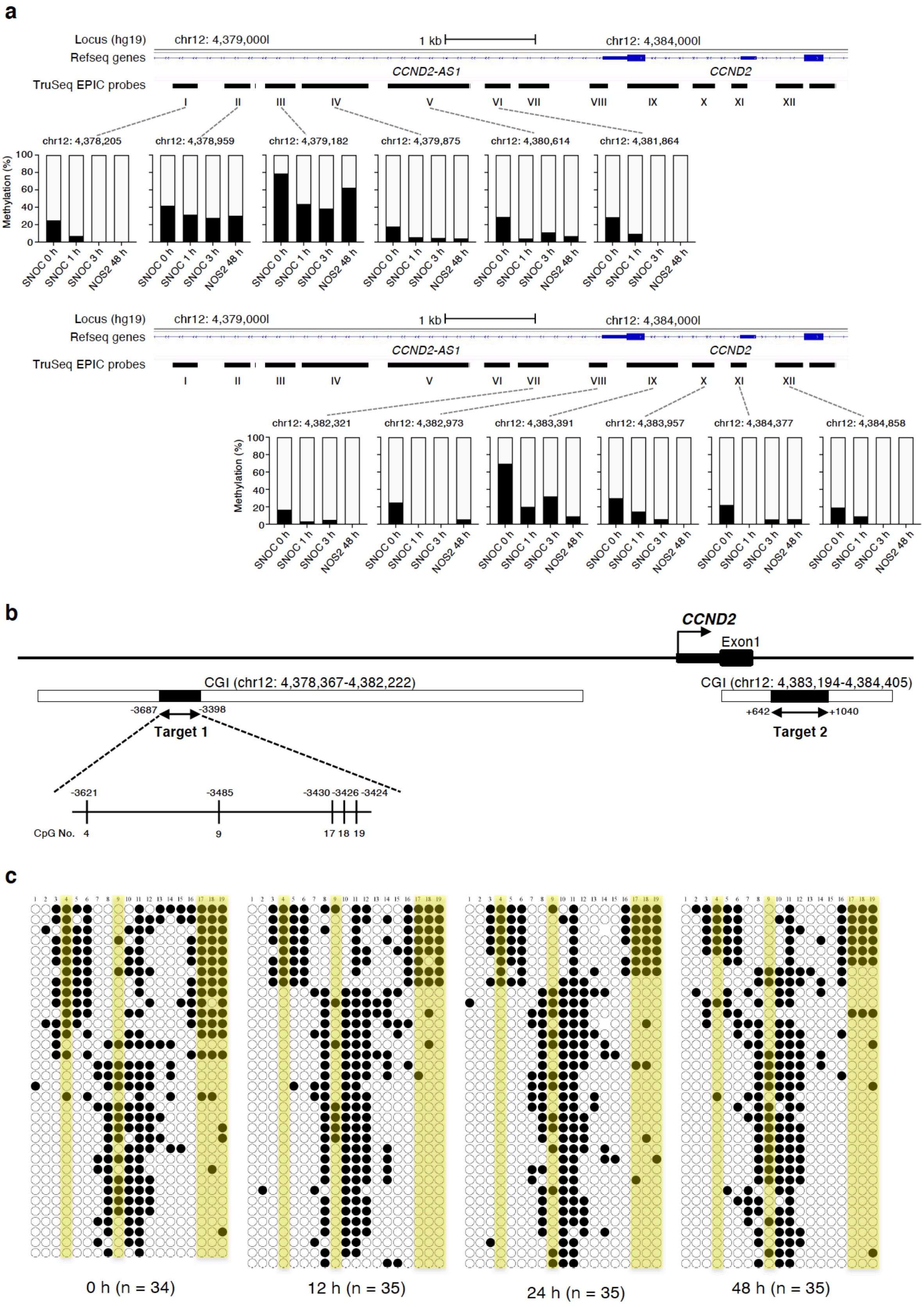
a
Locus (hg19)
chr12: 4,379,000
1 kb
chr12: 4,384,000
Refseq genes
CCND2-AS1
CCND2
TruSeq EPIC probes
I II III IV V VI VII VIII IX X XI XII
chr12: 4,378,205
chr12: 4,378,959
chr12: 4,379,182
chr12: 4,379,875
chr12: 4,380,614
chr12: 4,381,864
chr12: 4,382,321
chr12: 4,382,973
chr12: 4,383,391
chr12: 4,383,957
chr12: 4,384,377
chr12: 4,384,858
Methylation (%)
0 20 40 60 80 100
SNOC 0 h
SNOC 1 h
SNOC 3 h
NOS2 48 h
b
CCND2
Exon1
CGI (chr12: 4,378,367-4,382,222)
CGI (chr12: 4,383,194-4,384,405)
-3687
-3398
Target 1
+642
+1040
Target 2
-3621
-3485
-3430 -3426 -3424
CpG No. 4 9 17 18 19
c
0 h (n = 34)
12 h (n = 35)
24 h (n = 35)
48 h (n = 35)

**Supplementary Figure 5 | NO-induced demethylation in CpG sites of *Ccnd2* gene. a**, Representative methylated CpG sites in *Ccnd2* gene. **b,** Schematic representation of CpG islands in *Ccnd2* gene. Two different CpG islands, -3687 to -3398 (designated as Target 1) and +642 to +1040 (designated as Target 2) are indicated. **c**, HeLa cells were exposed to GSNO for varying periods, and DNA was then extracted for bisulfite sequencing. Methylation levels at CpG sites (Target 1) within the promoter region of *Ccnd2* were detected by bisulfite sequencing. Experiments were performed using 34 -35 independent samples. Closed circles, methylated CpG sites. Open circles, demethylated CpG sites. Source data are provided as a Source Data file.

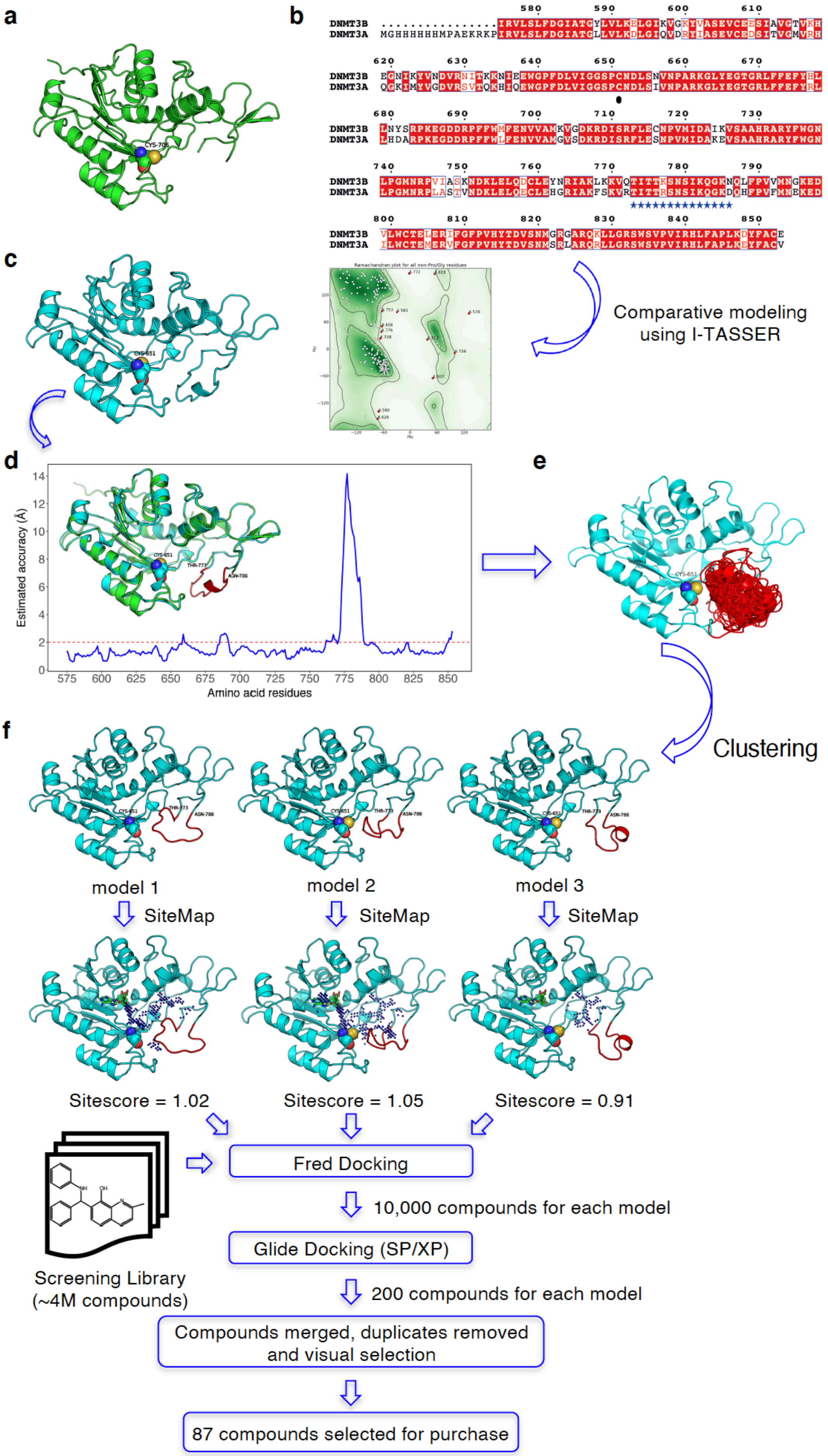

a
b
c
d
e
f
Comparative modeling using I-TASSER
Estimated accuracy (Å)
Amino acid residues
Clustering
model 1
model 2
model 3
SiteMap
Sitescore = 1.02
Sitescore = 1.05
Sitescore = 0.91
Fred Docking
10,000 compounds for each model
Glide Docking (SP/XP)
200 compounds for each model
Screening Library (~4M compounds)
Compounds merged, duplicates removed and visual selection
87 compounds selected for purchase

**Supplementary Figure 6 | Comparative modelling of DNMT3B. a,** DNMT3A crystal structure used as a template for homology modelling of DNMT3B. **b,** Sequence alignment of DNMT3B with DNMT3A. The catalytic residue, which is also the *S*-nitrosylation site, Cys651, is marked by a black circle. The loop from Thr773 to Asn786 is indicated by blue asterisks. **c**, Initial models were built using I-TASSER server. The model with the highest expected TM-Score was selected, and its Ramachandran plot is shown. **d,** The loop from Thr773 to Asn786 was predicted to have low accuracy. **e,** This loop was therefore rebuilt using the loop-modelling protocol of Rosetta. **f,** Virtual screening protocol. The top scoring 100 loop models were then clustered and three representative DNMT3B models were selected. Hierarchical structure-based virtual screening protocol to identify compounds interfering with *S*-nitrosylation without affecting enzymatic activity of DNMT3B. Small molecule binding pockets were first predicted on the surface of three DNMT3B models. Hierarchical molecular docking was then performed to filter a library of small molecules based on their energetic and geometric compatibilities. About one hundred molecules were finally selected based on diversity and interactions with the binding pocket.

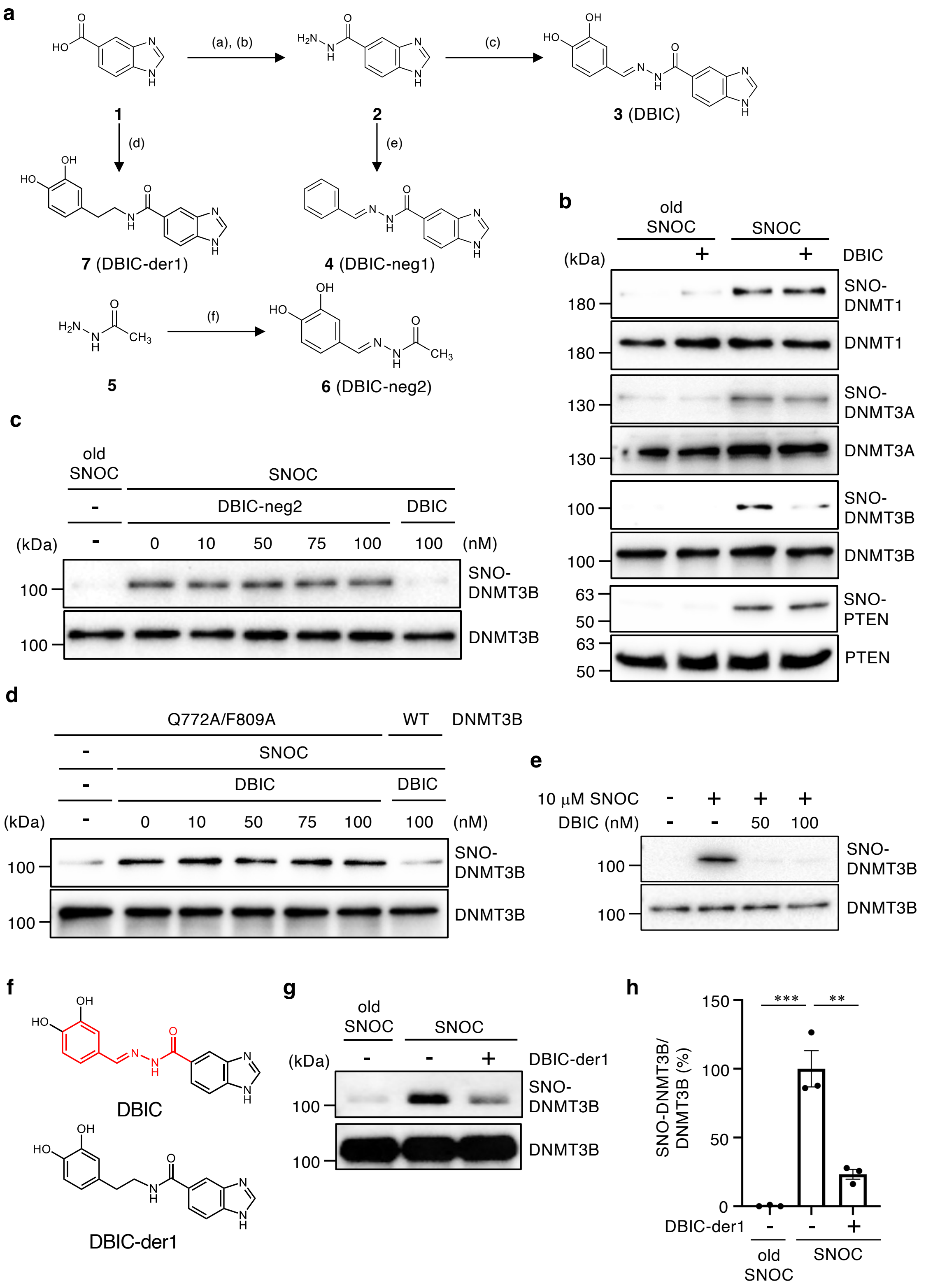


**Supplementary Figure 7 | Synthesis of compounds and their inhibitory effects on *S*-nitrosylation of DNMT3B. a**, Synthesis of compound 3 (DBIC), compound 4 (DBIC-neg1),

and compound 6 (DBIC-neg2). (a) EtOH, $H_2SO_4$, reflux, 24 hr; (b) $H_2N$-$NH_2$・$H_2O$, EtOH, reflux, 38 hr, 76%; (c) 3,4-dihydroxybenzaldehyde, DMF, 100 °C, 21.5 h, 91%; (d) 2-(3, 4-dihydroxyphenyl)ethylamine hydrochloride, 1-(3-dimethylaminopropyl)-3-ethylcarbodiimide, 4-dimethylaminopyridine, DMF, RT, 48 hr, 5.7%; (e) benzaldehyde, DMF, 100°C, 4.5 h, 84%; (f) 3,4-dihydroxybenzaldehyde, EtOH, reflux, 2 hr, 72%. **b**, Effects of DBIC (100 nM) on the formation of *S*-nitrosylation of DNMTs and PTEN. **c**, HEK293 cells were preincubated with varying concentrations of DBIC-neg2 or DBIC for 1 hr prior to SNOC exposure. SNO-DNMT3B formation was detected by biotin-switch assay. The data shown represent one of two separate experiments. **d**, HEK293 cells were transfected with WT- or Q772/F809A-DNMT3B, and were then preincubated with varying concentrations of DBIC for 1 hr prior to SNOC exposure. SNO-DNMT3B formation was detected by biotin-switch assay. The data shown represent one of four separate experiments. **e**, DBIC inhibits formation of recombinant SNO-DNMT3B *in vitro*. **f,** Chemical structures of DBIC and DBIC-der1. The benzylidene carbohydrazide of DBIC is shown in red. **g,** Effects of DBIC-der1 (100 nM) on formation of *S*-nitrosylation of DNMT3B. **h,** Ratio of SNO-DNMT3B/total DNMT3B quantified by densitometry. Values are mean ± s.e.m. ($n$ = 3; $^{**}P < 0.01$, $^{***}P < 0.001$ by one-way ANOVA with Bonferroni's *post-hoc* test). Source data are provided as a Source Data file.

**a**

| Target Class | Assay Target | Mode | % Response Average |
|---|---|---|---|
| GPCR | ADORA2A | Agonist | 0 |
| | ADORA2A | Antagonist | 24.7 |
| GPCR | ADRA1A | Agonist | 0.3 |
| | ADRA1A | Antagonist | 0 |
| GPCR | ADRA2A | Agonist | 16 |
| | ADRA2A | Antagonist | 0 |
| GPCR | ADRB1 | Agonist | 0 |
| | ADRB1 | Antagonist | 0 |
| GPCR | ADRB2 | Agonist | 0.1 |
| | ADRB2 | Antagonist | 3.9 |
| GPCR | AVPR1A | Agonist | 0.5 |
| | AVPR1A | Antagonist | 0 |
| GPCR | CCKAR | Agonist | 0.5 |
| | CCKAR | Antagonist | 0 |
| GPCR | CHRM1 | Agonist | 0.4 |
| | CHRM1 | Antagonist | 0.3 |
| GPCR | CHRM2 | Agonist | 13.2 |
| | CHRM2 | Antagonist | 0 |
| GPCR | CHRM3 | Agonist | 0.3 |
| | CHRM3 | Antagonist | 0 |
| GPCR | CNR1 | Agonist | 2.5 |
| | CNR1 | Antagonist | 0 |
| GPCR | CNR2 | Agonist | 4.5 |
| | CNR2 | Antagonist | 7.2 |
| GPCR | DRD1 | Agonist | 0.7 |
| | DRD1 | Antagonist | 6.9 |
| GPCR | DRD2S | Agonist | 0 |
| | DRD2S | Antagonist | 2.4 |
| GPCR | EDNRA | Agonist | 1 |
| | EDNRA | Antagonist | 0 |
| GPCR | HRH1 | Agonist | 0.6 |
| | HRH1 | Antagonist | 0 |
| GPCR | HRH2 | Agonist | 0 |
| | HRH2 | Antagonist | 7.4 |
| GPCR | HTR1A | Agonist | 5.4 |
| | HTR1A | Antagonist | 0.2 |
| GPCR | HTR1B | Agonist | 0 |
| | HTR1B | Antagonist | 0.3 |
| GPCR | HTR2A | Agonist | 0 |
| | HTR2A | Antagonist | 0 |
| GPCR | HTR2B | Agonist | 0 |
| | HTR2B | Antagonist | 9.7 |
| GPCR | OPRD1 | Agonist | 4.9 |
| | OPRD1 | Antagonist | 3.8 |
| GPCR | OPRK1 | Agonist | 1.6 |
| | OPRK1 | Antagonist | 0 |
| GPCR | OPRM1 | Agonist | 0.6 |
| | OPRM1 | Antagonist | 18.1 |
| NHR | AR | Agonist | 0 |
| | AR | Antagonist | 0 |
| NHR | GR | Agonist | 0.3 |
| | GR | Antagonist | 3.1 |
| Transporter | DAT | Blocker | 5.9 |
| Transporter | NET | Blocker | 0 |
| Transporter | SERT | Blocker | 5.3 |
| Ion Channel | CAV1.2 | Blocker | 0 |
| Ion Channel | GABAA | Opener | 0 |
| | GABAA | Blocker | 0 |
| Ion Channel | hERG | Blocker | 1.4 |
| Ion Channel | HTR3A | Opener | 0.6 |
| | HTR3A | Blocker | 0 |
| Ion Channel | KvLQT1/minK | Opener | 8.1 |
| | KvLQT1/minK | Blocker | 7.7 |
| Ion Channel | nAChR(a4/b2) | Opener | 0.2 |
| | nAChR(a4/b2) | Blocker | 0 |
| Ion Channel | NAV1.5 | Blocker | 2.3 |
| Ion Channel | NMDAR (1A/2B) | Opener | 0 |
| | NMDAR (1A/2B) | Blocker | 0 |

**b**

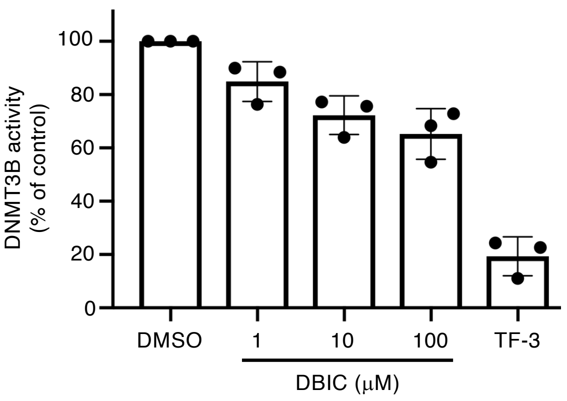


**c**

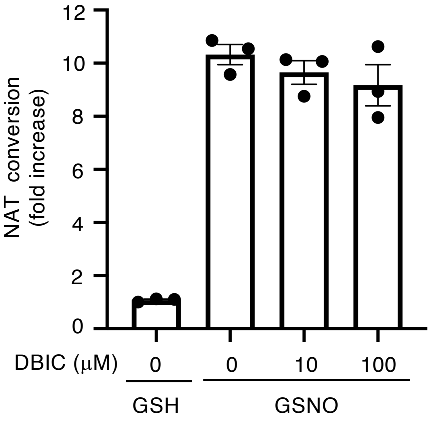


**d**

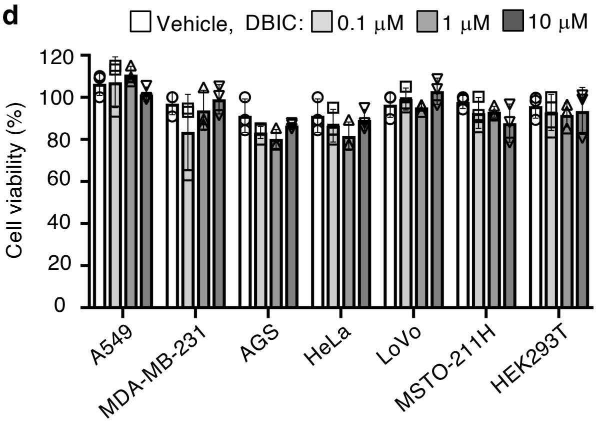

**Supplementary Figure 8 | Biological characterization of DBIC. a**, Pharmacological profile screening for 10 μM DBIC. Data were generated by DiscoverX via the Safety47$^{TM}$ panel of functional assays for human targets for safety and off-target screening in a cell-based system. Responses under 50% are considered insignificant in this assay according to the manufacturer (DiscoverX, Eurofins). **b**, Effect of DBIC on enzymatic activity of DNMT3B. Recombinant DNMT3B was incubated *in vitro* with various concentrations of DBIC or theaflavin 3,3'-digallate (TF-3) for 1 hr. DNMT3B enzymatic activity was assayed with the DNMT Direct Activity Assay Kit. Values are mean ± s.e.m. ($n$ = 3). **c**, Inability of DBIC to scavenge NO. The indicated concentration of DBIC was incubated with fresh 10 μM GSNO for 20 min at RT. Then, 2, 3-diaminonaphthalene (DAN) (100 μM) was incubated for 30 min. Conversion of 2, 3-naphthotriazole (NAT) from DAN was measured spectrofluorometrically in order to follow the amount of *S*-nitrosothiol in the solution. If DBIC had scavenged NO, then nitrosothiol species (derived from GSNO) would not have been detected by this assay, but in fact nitrosothiol levels remained statistically unaltered in the presence of DBIC. Values are mean ± s.e.m. ($n$ = 3). **d**, No cytotoxic effect of DBIC in several types of cell lines. Cells were incubated with various concentrations of DBIC. After 48-h incubation, MTT (3-(4,5-dimethylthiazol-2-yl)-2,5-diphenyltetrazolium bromide) assay was performed. The relative cell viability compared to control (DMSO treated) cells was calculated as below: Cell viability (%) = OD595 DBIC/OD595 DMSO × 100. Values are mean ± s.e.m. ($n$ = 3). Source data are provided as a Source Data file.

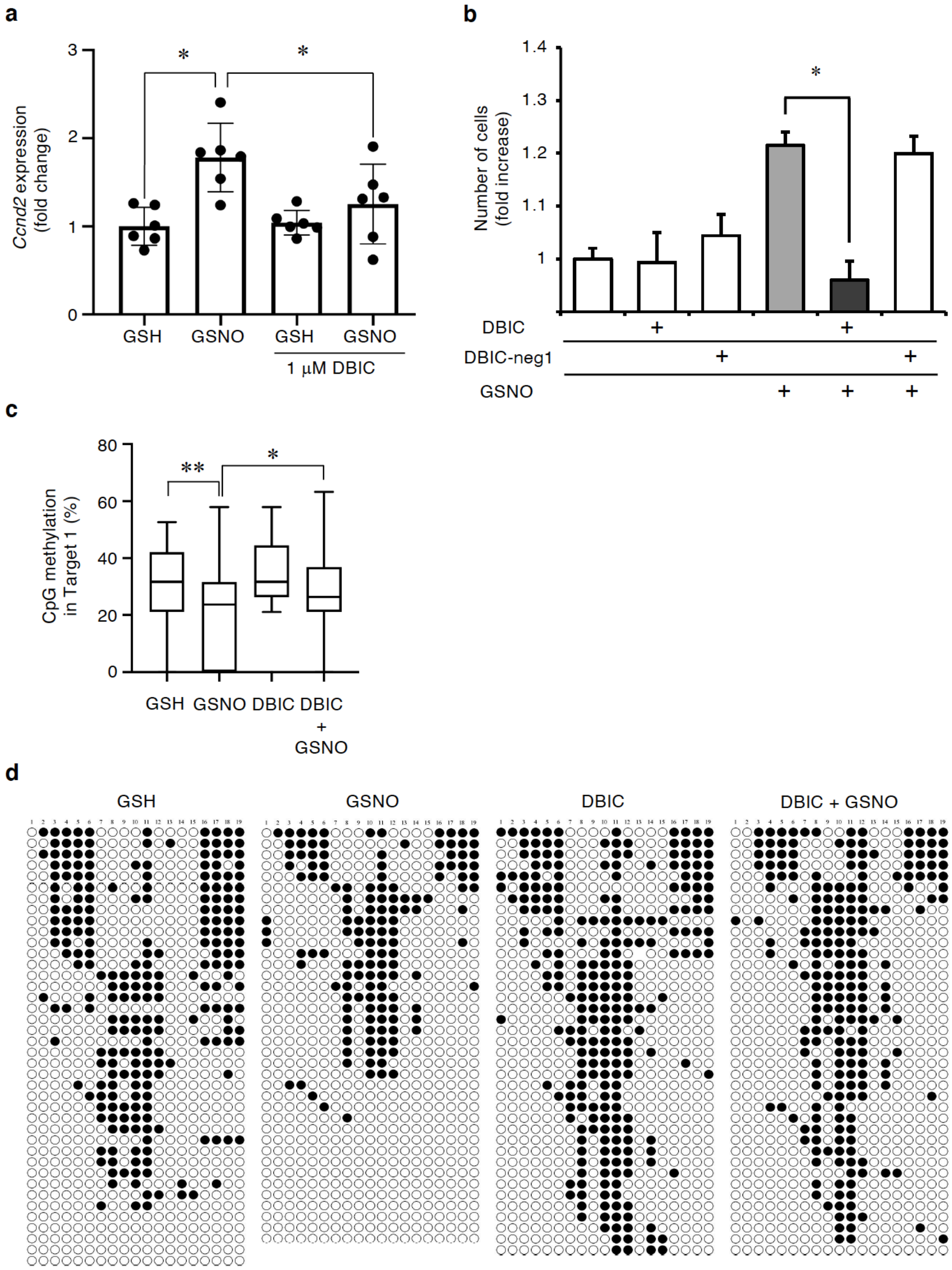


**Supplementary Figure 9 | Effect of DBIC on CpG methylation. a,** Effect of DBIC on GSNO-induced *Ccnd2* gene expression in HeLa cells. *Ccnd2* mRNA levels were detected by

qPCR. Values are expressed as mean ± s.e.m. ($n = 6$, *$P < 0.05$, **$P < 0.01$ by one-way ANOVA with Tukey's *post-hoc* test). **b**, Effect of DBIC on cell proliferation evoked by NO. AGS cells were preincubated with 100 nM DBIC or DBIC-neg1 for 1 hr, and then exposed to 10 μM GSNO. After a 72-h incubation, cells were stained with Hoechst 33258 and observed by epifluorescence microscopy. The number of cells was counted in six randomly chosen fields. Values are mean ± s.e.m. ($n$ = 6-18; ***$P < 0.001$ by one-way ANOVA with Tukey's *post-hoc* test, ns: not significant). **c,d**, HeLa cells were exposed to DBIC for 1 hr prior to GSNO (100 μM) exposure. After 24 hr, methylation levels of CpG sites (Target 1) within the promoter region of *Ccnd2* were detected by bisulfite sequencing. NO-induced decreases in methylated CpG were ameliorated by DBIC treatment. For box plots (**c**), the center lines represent the median, and the box limits are the 25th and 75th percentiles. Whiskers outline minimum to maximum values. Whiskers indicate minimum to maximum values ($n$ = 38-40; *$P < 0.05$, **$P < 0.01$ by two-tailed Wilcoxon's rank-sum test). Closed circles, methylated CpG sites. Open circles, demethylated CpG sites. Source data are provided as a Source Data file.

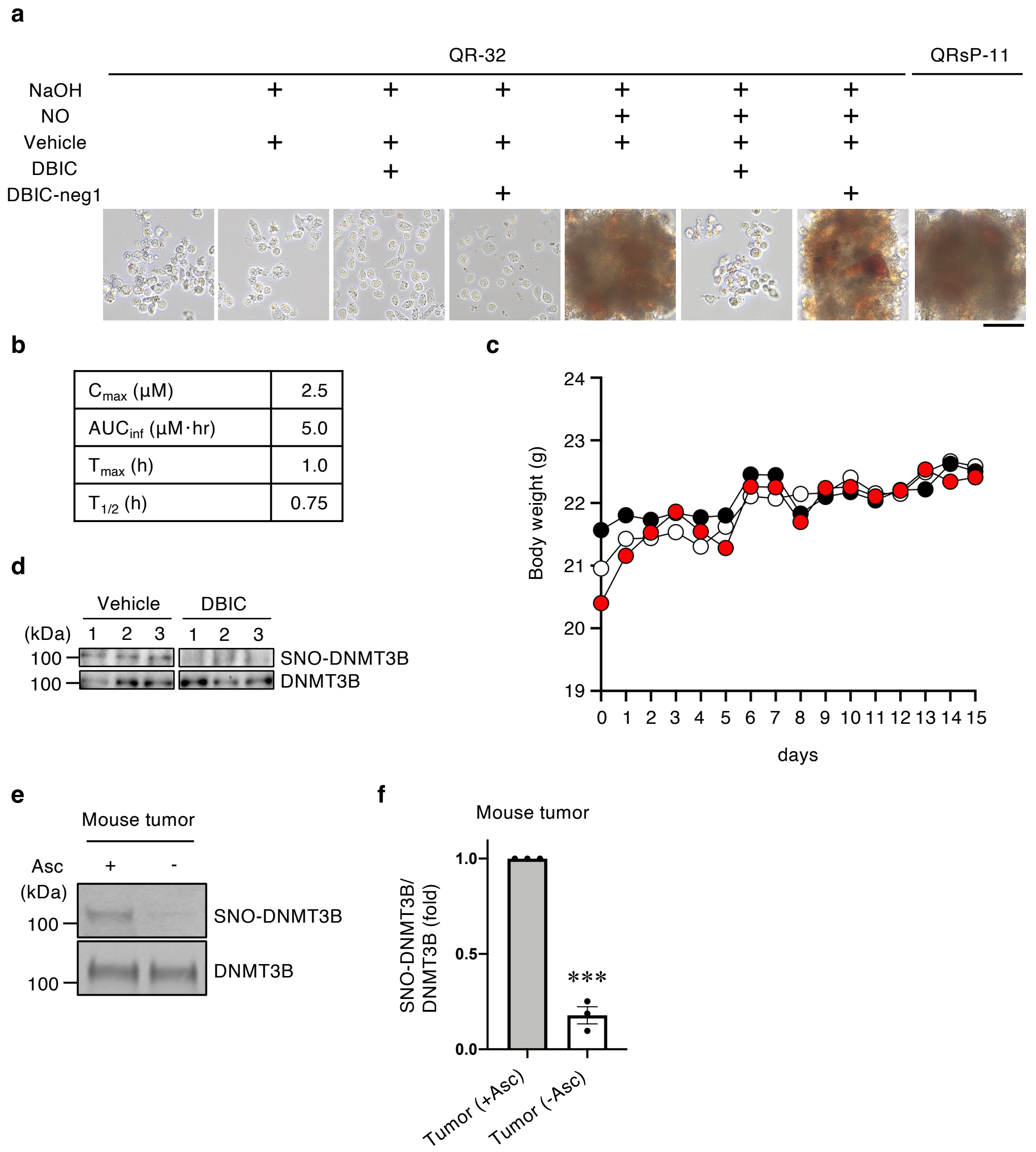


**Supplementary Figure 10 | Effects of DBIC *in vivo*. a,** Poorly tumorigenic and nonmetastatic mouse QR-32 cells were serially exposed to 126 μM of the NO donor NOC18 every 3 days for 1 month in the presence or absence of 100 nM DBIC. The cells were then transferred into 3D culture. Spheroidal aggregate formation of QR-32 cells and QRsP-11 tumor cells (as a positive control) were assessed 3 days after plating. Scale bar, 50 μm. All data shown represent one of six separate experiments. **b**, Pharmacokinetic parameters after single intraperitoneal (i.p.) administration of DBIC (25 mg/kg). $C_{max}$ (maximum plasma concentration), $AUC_{inf}$ (area under-the-curve concentration from time zero to infinity), $T_{max}$

(time of maximum plasma concentration), $T_{1/2}$ (plasma half–life). **c,** Changes in mouse body weight during 15 days of DBIC administration (n=6). Open circles, no treatment. Closed circles, vehicle treatment. Red circles, 25 mg/kg/day DBIC treatment. **d**, Effect of DBIC on decreasing SNO-DNMT3B formation *in vivo* in subcutaneous tumor (quantified in **Fig. 4g**). **e**, SNO-DNMT3B formation in mouse tumors. **f**, Ratio of SNO-DNMT3B/total DNMT3B quantified by densitometry. Values are mean ± s.e.m. ($n = 3$; ***$P < 0.001$ by two-tailed Student's $t$-test). Source data are provided as a Source Data file.

**Supplementary Figure 11 | Whole process concerning synthesis of compound 3 (DBIC), compound 4 (DBIC-neg1), compound 6 (DBIC-neg2) and compound 7 (DBIC-der1).**

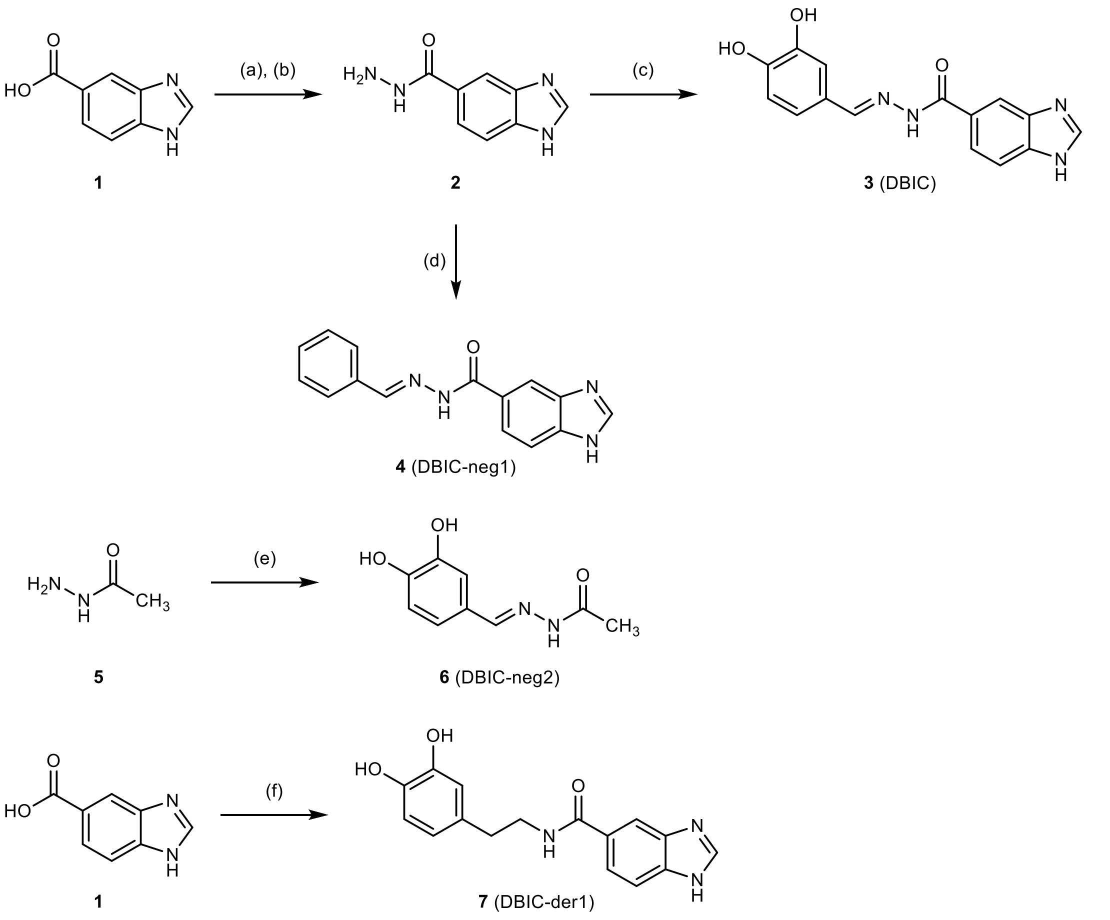


Melting points were determined on a Yanagimoto micro melting point apparatus. $^{1}$H NMR spectra were run on a Varian 400-MR (400 MHz). A silica gel column, Chemcosorb 5Si-U (Chemco). Merck silica gel 60 (230 ± 400 mesh) .

**Supplementary Figure 12 | Synthesis of Compound 2 (commercially available).**

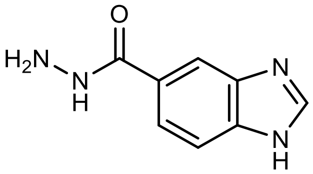

To a solution of 1*H*-benzo[*d*]imidazole-5-carboxylic acid (**1,** 1.00 g, 6.17 mmol) in EtOH (15 ml), conc. $H_2SO_4$ (0.5 ml) was added dropwise. The mixture was refluxed for 19.5 h. Ice water (170 ml) was added to the reaction solution and was adjusted pH 6 by $NaHCO_3$. The solution was extracted by AcOEt (100 ml x 3), the organic layer was washed with brine, dried by $MgSO_4$, and the solvent was removed to give oily product (1.05 g, 5.54 mmol), which was used without the further purification. A mixture of the oily product (1.04 g, 5.33 mmol) and hydrazine hydrate (1.04 ml, 21.3 mmol) in EtOH (5 ml) was refluxed for 66.5 h. After cooling, the resulting crystals were filtered and washed with EtOH to give **2** (0.826 g, 4.69 mmol) in 76% yield, purple sandiness, mp 253–257°C; $^1$H-NMR（400 MHz, DMSO-$d_6$）δ：4.56 (2H, s), 7.68 (1H, d, $J$=8.4 Hz), 7.79 (1H, dd, $J$=8.4 Hz), 8.18 (1H, s), 8.40 (1H, s), 9.83 (1H, s), 8.34 (1H, s), 12.69 (1H, s)

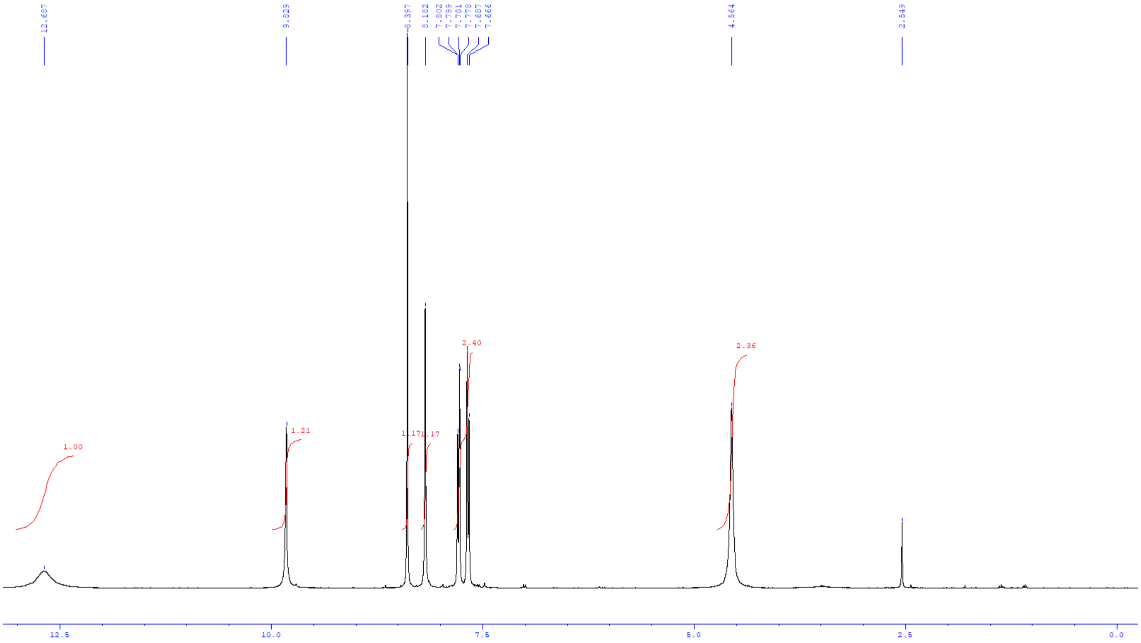

**Supplementary Figure 13 | Synthesis of Compound 3 (DBIC, commercially available).**

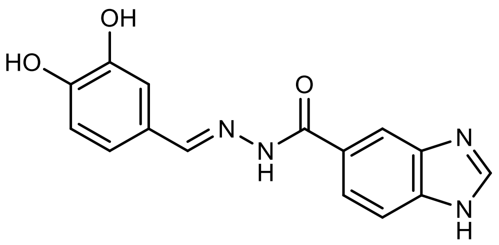

A mixture of **2** (0.10 g, 0.57 mmol) and 3,4-dihydroxybenzaldehyde (0.79 mg, 0.57 mmol) in DMF (3 ml) was heated at 100°C for 21.5 h. After cooling, water (300 ml) was added to the mixture. The resulting crystals were filtered and washed with water and EtOH to give **3** (0.154 g, 0.52 mmol) in 91% yield, colorless sandiness, mp 286─290°C; $^{1}$H-NMR（400 MHz, DMSO- $d_6$）δ：6.84 (1H, d, $J$=8.0 Hz), 6.98 (1H, dd, $J$=8.0 Hz), 7.30 (1H, d, $J$=1.2 Hz), 7.72 (1H, d, $J$=8.4 Hz), 7.84 (1H, d, $J$=8.4 Hz), 8.27 (1H, s), 8.34 (1H, s), 8.42 (1H, s), 9.34 (1H, s), 9.44 (1H, s), 11.67 (1H, s), 12.80 (1H, s); analysis (% calcd, % found for $C_{15}H_{12}N_4O_3 \cdot 1/2\ H_2O$: C (59.01, 59.14), H (4.29, 4.18), N (18.35, 18.19); $^{13}$C-NMR (400 MHz, DMSO-$d_6$, ppm) δ: 113.88, 113.91, 115.29, 115.43, 116.67, 121.58, 126.51, 126.64, 131.53, 132.21, 133.65, 143.27, 143.28, 146.73, 149.11, 149.94, 162.94.

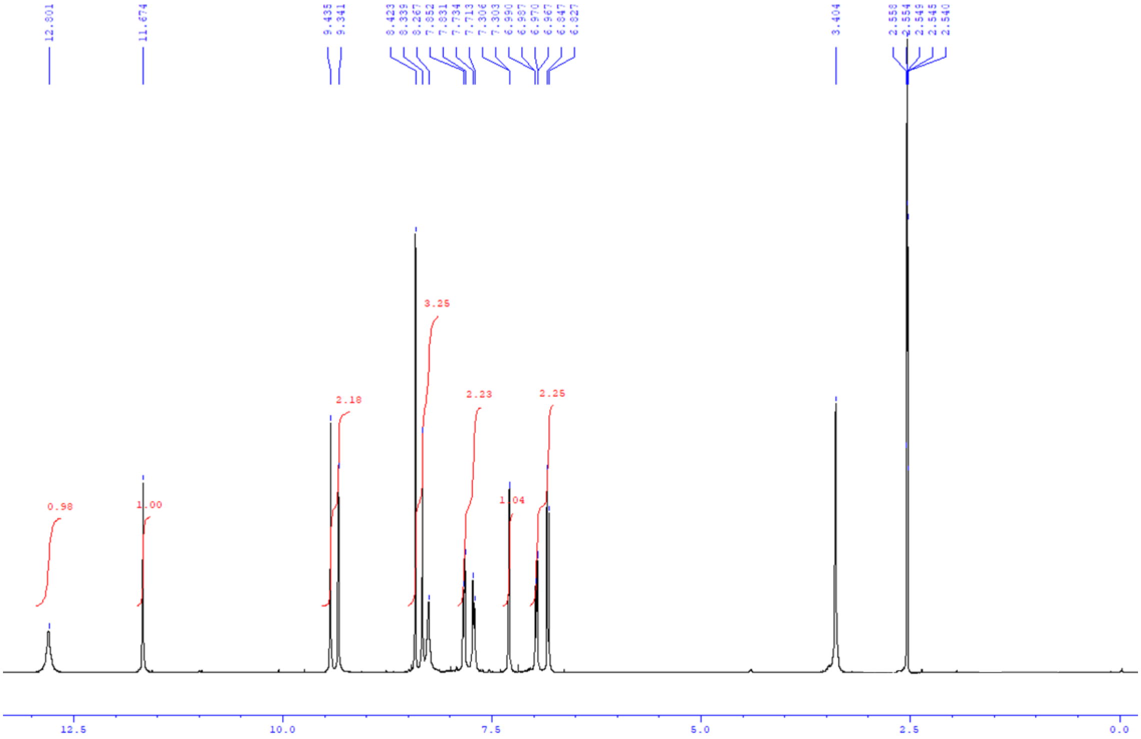

12.801
11.674
9.435
9.341
8.423
8.339
8.267
7.852
7.831
7.734
7.713
7.306
7.303
6.990
6.987
6.970
6.967
6.847
6.827
3.404
2.558
2.554
2.549
2.545
2.540
0.98
1.00
2.18
3.25
2.23
1.04
2.25
12.5
10.0
7.5
5.0
2.5
0.0


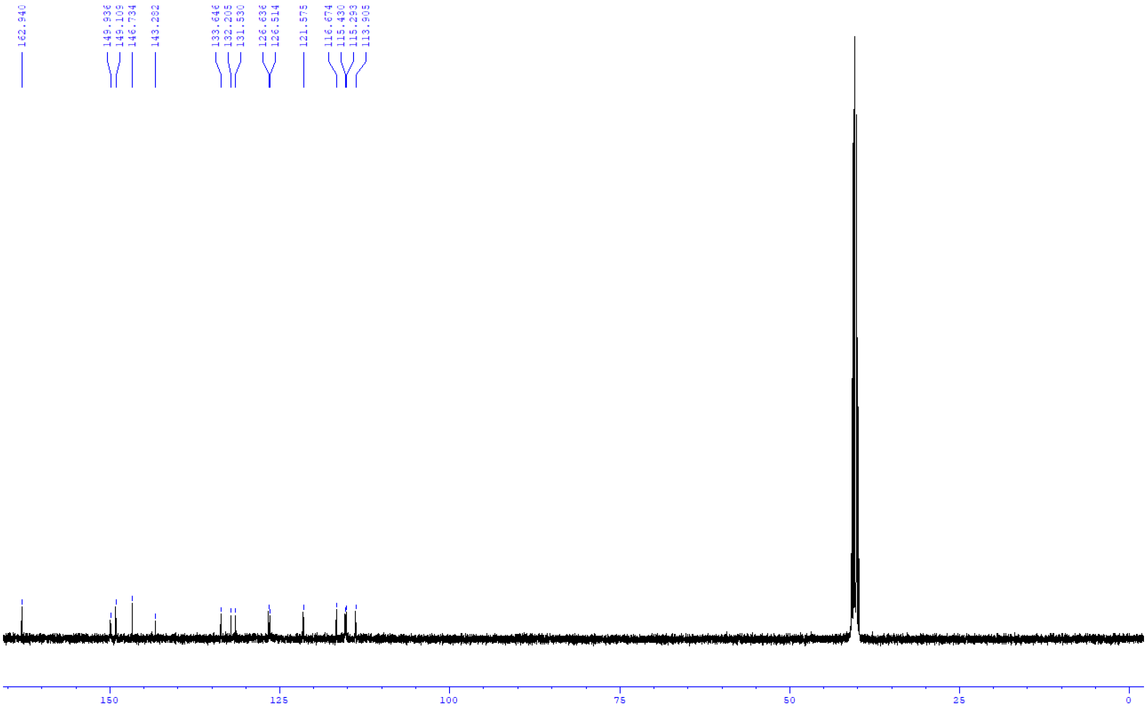

162.940
149.936
149.109
146.734
143.282
133.646
132.205
131.530
126.636
126.514
121.575
116.674
115.430
115.293
113.905
150
125
100
75
50
25
0

**Supplementary Figure 14 | Synthesis of Compound 4 (DBIC-neg1, commercially available).**

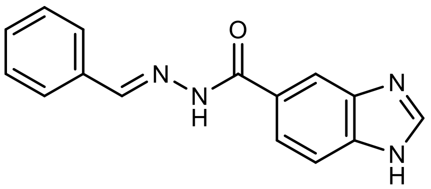

A mixture of **2** (0.10 g, 0.57 mmol) and benzaldehyde (0.69 ml, 0.68 mmol) in DMF (3 ml) was heated at 100°C for 4.5 h. After cooling, water (200 ml) was added to the mixture. The resulting crystals were filtered and washed with water and EtOH to give **4** (0.127 g, 0.48 mmol) in 84% yield, colorless sandiness, mp 298–301°C; $^{1}$H-NMR（400 MHz, DMSO-$d_6$）δ：7.50-7.52 (3H, m), 7.78-7.88 (4H, m), 8.31 (1H, brs), 8.44 (1H, s), 8.54 (1H, s), 11.93 (1H, s), 12.82 (1H, s); analysis (% calcd, % found for $C_{15}H_{12}N_4O$): C (68.17, 67.90), H (4.58, 4.47), N (21.20, 21.05); $^{13}$C-NMR (400 MHz, DMSO-$d_6$, ppm) δ: 115.53, 115.83, 122.07, 122.81, 127.94, 129.75, 130.86, 135.42, 139.07, 140.52, 144.70, 148.17, 164.57.

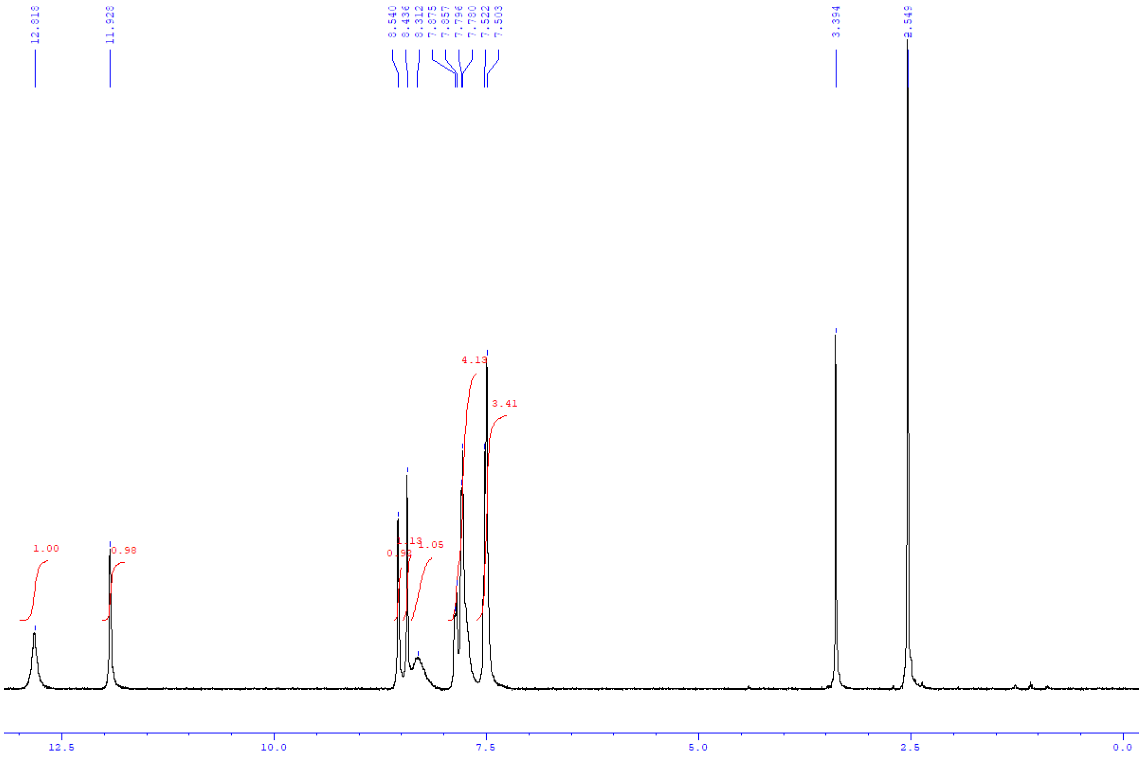

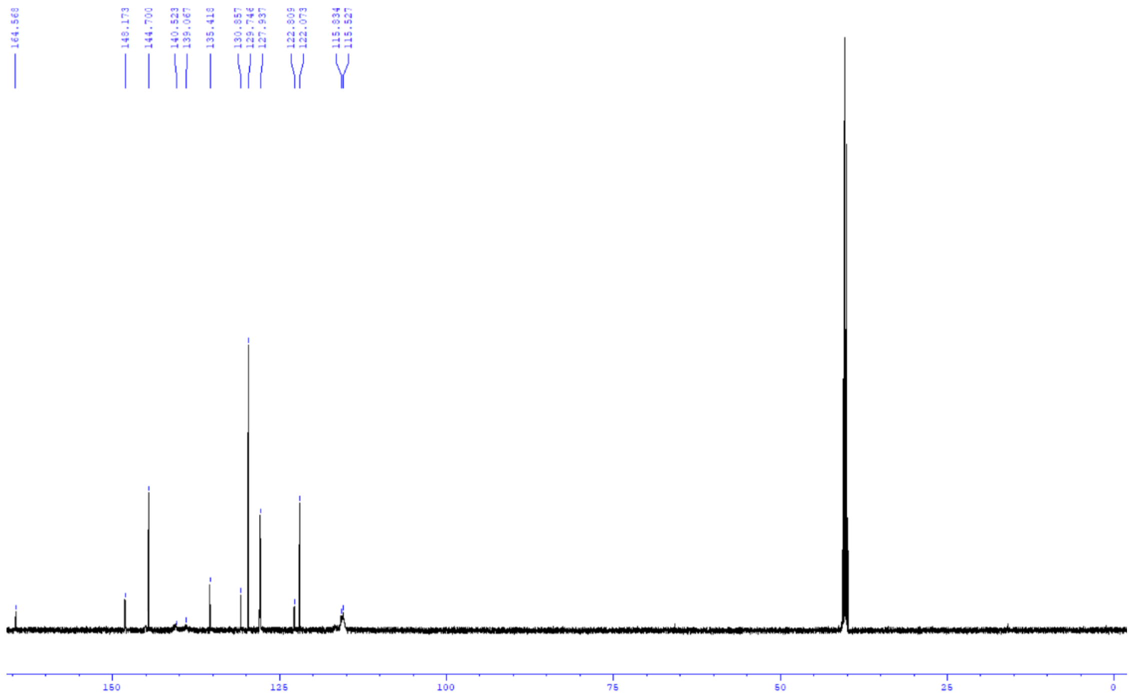

164.568
148.173
144.700
140.523
139.067
135.418
130.857
129.746
127.937
122.809
122.073
115.834
115.527
150
125
100
75
50
25
0

**Supplementary Figure 15 | Synthesis of Compound 6 (DBIC-neg2, commercially available).**

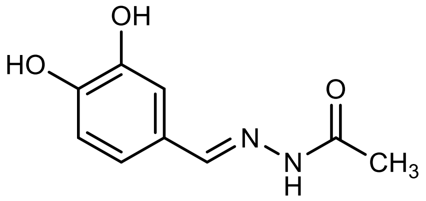

To a solution of acetohydrazide (**5,** 0.200 g, 2.70 mmol) and 3,4-dihydroxybenzaldehyde (0.373 mg, 2.70 mmol) in EtOH (5 ml) was added. The mixture was stirred at reflux for 2 h. After cooling, the resulting crystals were filtered to give the *E*,*Z* mixture (3:2) **6** (0.378 g, 1.95 mmol) in 72% yield, mp 208—217°C; $^{1}$H-NMR（400 MHz, DMSO-$d_6$） ***E*-form**; δ：1.89—2.14 (3H, m), 6.74 (1H, d, J=8.0 Hz), 6.83—6.87 (1H, m), 7.10 (1H, d, J=2.0 Hz), 7.78 (1H, s), 9.17 (1H), 9.32 (1H), 10.98—11.09 (1H, m). ***Z*-form**; δ：1.89—2.14 (3H, m), 6.74 (1H, d, *J*=8.0 Hz), 6.83—6.87 (1H, m), 7.15 (1H, d, *J*=2.0 Hz), 7.92 (1H, s), 9.17 (1H), 9.32 (1H), 10.98—11.09 (1H, m) ; analysis (% calcd, % found for $C_9H_{10}N_2O_3$): C (55.67, 55.54), H (5.19, 5.14), N (14.43, 14.38); $^{13}$C-NMR (400 MHz, DMSO-$d_6$, ppm) *E*-form δ: 20.77, 112.96, 116.11, 120.41, 126.29, 143.70, 146.20, 148.07, 172.07; *Z*-form δ: 22.15, 113.13, 116.04, 120.91, 126.32, 143.70, 146.63, 148.28, 165.72.

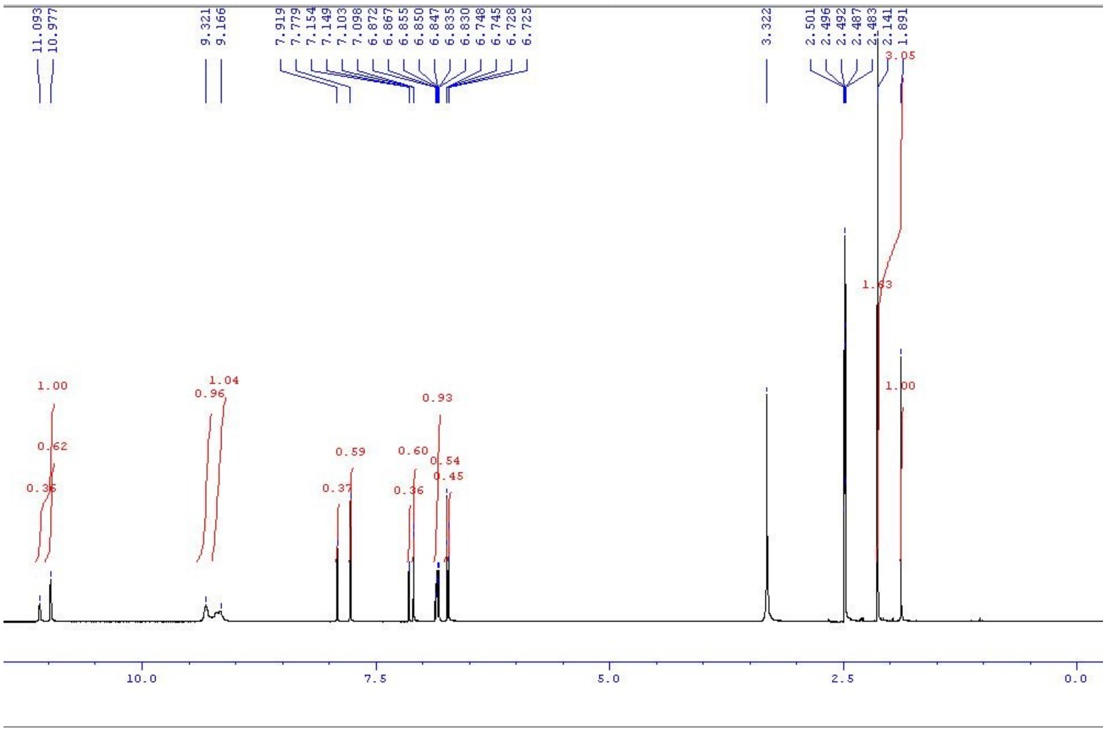

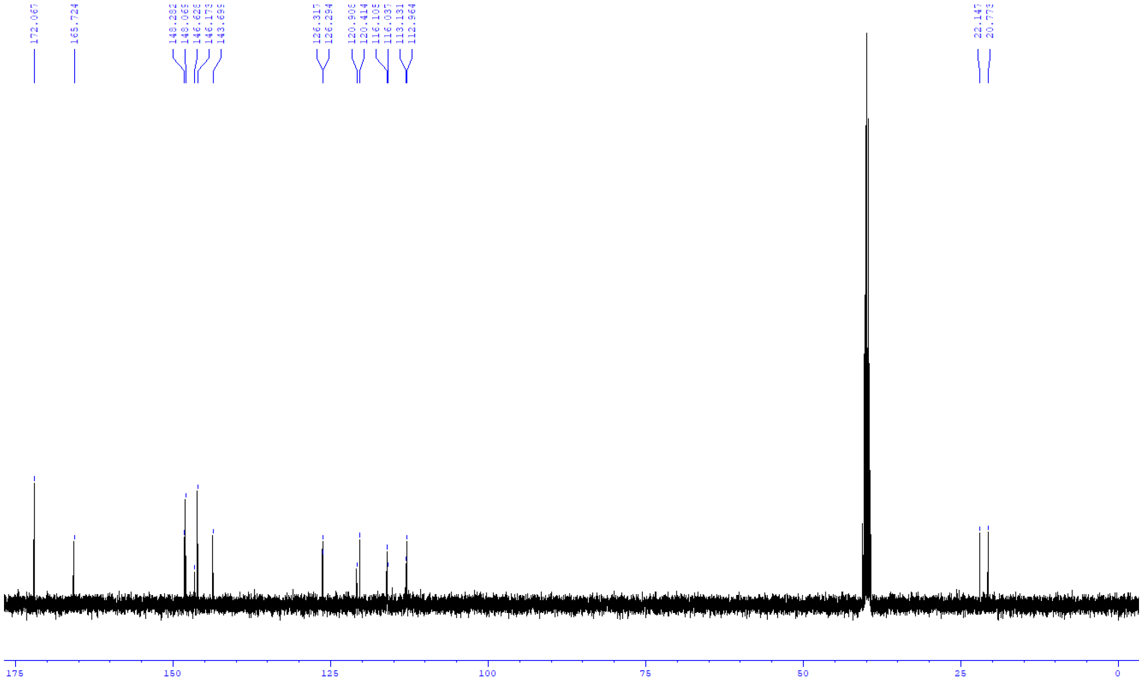
172.067
165.724
148.282
148.065
146.628
146.173
143.695
126.317
126.294
120.908
120.414
116.105
116.037
113.131
112.964
22.147
20.773
175
150
125
100
75
50
25
0

**Supplementary Figure 16 | Synthesis of Compound 7 (DBIC-der1).**

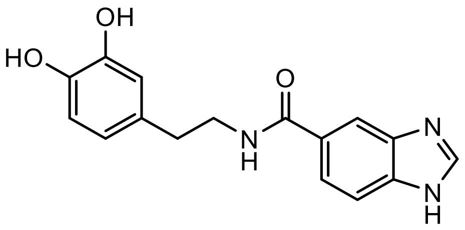

To a solution of 1*H*-benzo[*d*]imidazole-5-carboxylic acid (**1**, 324.3 mg, 2.0 mmol), 2-(3, 4-dihydroxyphenyl)-ethylamine hydrochloride (379.3 mg, 2.0 mmol), 1-(3-dimethylaminopropyl)-3-ethylcarbodiimide (421.7 mg, 2.2 mmol) and 4-dimethylaminopyridine (268.8 mg, 2.2 mmol) in DMF (10 mL) was added. The mixture was stirred at room temperature for 48 h. After stirring, the reaction solution was purified by silica gel column chromatography ($CHCl_3$/MeOH, 5:1) to gain **7** (34.1 mg, 0.12 mmol) in 5.7% yield, colorless sandiness; mp 210─219°C (decomposition), $^1$H-NMR（400 MHz, DMSO-$d_6$, ppm) δ: 2.71 (2H, t, *J*=7.6 Hz), 3.45 (2H, q, *J*=7.6 Hz), 6.53 (1H, dd, *J*=8.0 Hz), 6.68-6.70 (2H, m), 7.59─7.80 (2H, m), 8.08─8.39 (2H, m), 8.55 (1H, brs), 8.70 (1H, s), 8.83 (1H, s), 12.69─12.77 (1H, m); HRMS (ESI) m/z: Calcd for $C_{24}H_{15}N_3NaO_3$: 320.1011; Found: 320.1006 $[M^{+}Na]^{+}$; $^{13}$C-NMR (150 MHz, DMSO-$d_6$, ppm) δ: 32.90, 40.87, 115.30, 116.32, 116.59, 119.75, 121.68, 127.58, 128.50, 144.29, 144.56, 145.80, 167.17.

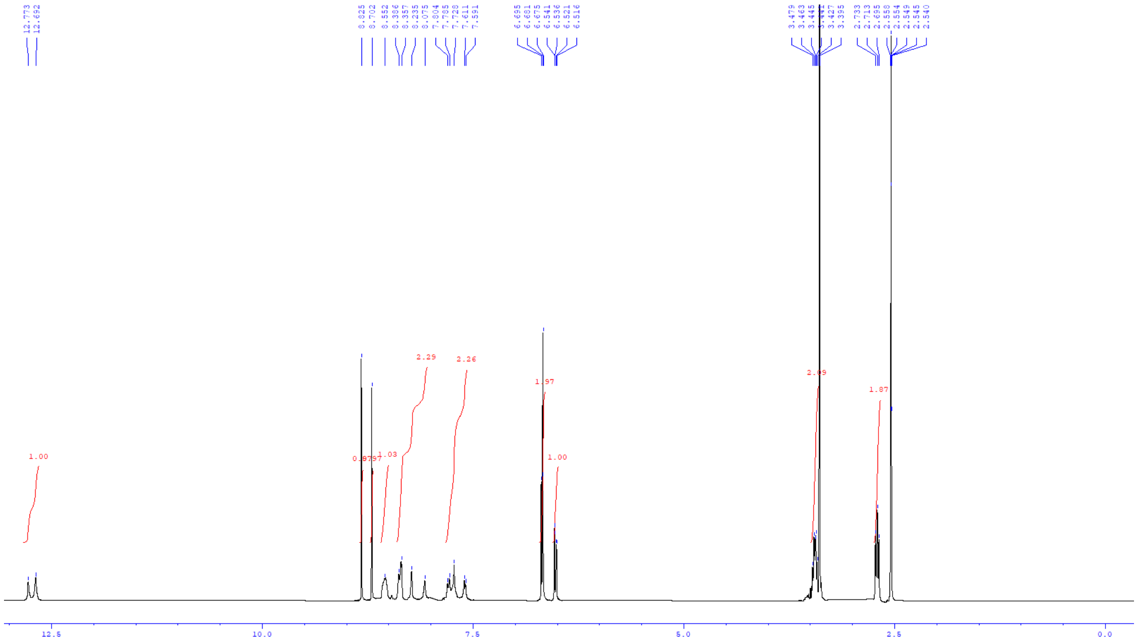

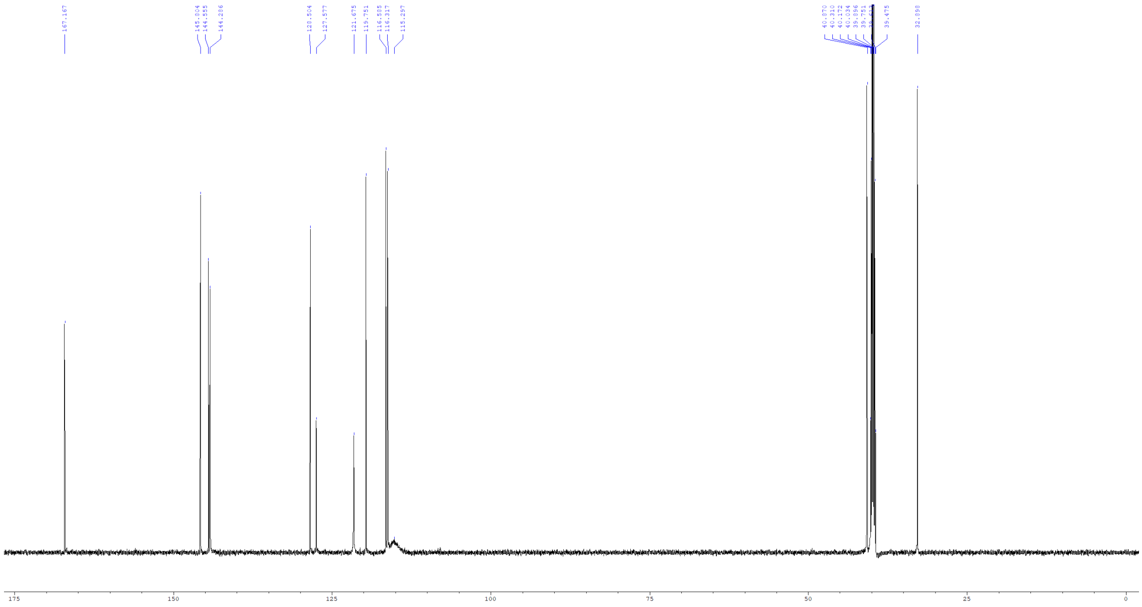

**Supplementary Data 1 | Changes in cytosine methylation status in response to NO exposure.** HeLa cells were either transduced with NOS2 for 48 hr or exposed to 200 μM SNOC, 1 or 3 h after which genomic DNA was isolated from the cells and then fragmented. Target bisulfide sequencing libraries were prepared, followed by 101 bp paired-end sequencing on a NextSeq550 system. The ratio of the number of sequenced methylated cytosine reads to the total number of reads for each locus was evaluated.
