# Supplementary material for: Pivotal role for S-nitrosylation of DNA methyltransferase 3B in epigenetic regulation of tumorigenesis
Source: Nat Commun. 2023 Feb 4;14:621. doi: 10.1038/s41467-023-36232-6 (PMC9899281; doi:10.1038/s41467-023-36232-6)
Supplement: Supplementary file 2 — Description of additional Supplementary File [file 41467_2023_36232_MOESM2_ESM.pdf]

## **Descriptions of additional Supplementary Files**

Supplementary Data 1 | Changes in cytosine methylation status in response to NO exposure. HeLa cells were either transduced with NOS2 for 48 hr or exposed to 200 μM SNOC, 1 or 3 h after which genomic DNA was isolated from the cells and then fragmented. Target bisulfide sequencing libraries were prepared, followed by 101 bp paired-end sequencing on a NextSeq550 system. The ratio of the number of sequenced methylated cytosine reads to the total number of reads for each locus was evaluated.
