# Supplementary material for: Pivotal role for S-nitrosylation of DNA methyltransferase 3B in epigenetic regulation of tumorigenesis
Source: Nat Commun. 2023 Feb 4;14:621. doi: 10.1038/s41467-023-36232-6 (PMC9899281; doi:10.1038/s41467-023-36232-6)
Supplement: Supplementary file 4 — Reporting Summary [file 41467_2023_36232_MOESM4_ESM.pdf]

nature portfolio

Corresponding author(s): Stuart A. Liptom
Takashi Uehara

Last updated by author(s): Dec 20, 2022

Reporting Summary

Nature Portfolio wishes to improve the reproducibility of the work that we publish. This form provides structure for consistency and transparency in reporting. For further information on Nature Portfolio policies, see our Editorial Policies and the Editorial Policy Checklist.

Statistics

For all statistical analyses, confirm that the following items are present in the figure legend, table legend, main text, or Methods section.

n/a	Confirmed	
☐	☒	The exact sample size (*n*) for each experimental group/condition, given as a discrete number and unit of measurement
☐	☒	A statement on whether measurements were taken from distinct samples or whether the same sample was measured repeatedly
☐	☒	The statistical test(s) used AND whether they are one- or two-sided<br>*Only common tests should be described solely by name; describe more complex techniques in the Methods section.*
☒	☐	A description of all covariates tested
☐	☒	A description of any assumptions or corrections, such as tests of normality and adjustment for multiple comparisons
☐	☒	A full description of the statistical parameters including central tendency (e.g. means) or other basic estimates (e.g. regression coefficient) AND variation (e.g. standard deviation) or associated estimates of uncertainty (e.g. confidence intervals)
☐	☒	For null hypothesis testing, the test statistic (e.g. *F*, *t*, *r*) with confidence intervals, effect sizes, degrees of freedom and *P* value noted<br>*Give P values as exact values whenever suitable.*
☒	☐	For Bayesian analysis, information on the choice of priors and Markov chain Monte Carlo settings
☒	☐	For hierarchical and complex designs, identification of the appropriate level for tests and full reporting of outcomes
☒	☐	Estimates of effect sizes (e.g. Cohen's *d*, Pearson's *r*), indicating how they were calculated

Our web collection on statistics for biologists contains articles on many of the points above.

Software and code

Policy information about availability of computer code

Data collection	ChemiDoc MP Imaging System with Image Lab version 5.2.1 (Bio-Rad Laboratories) for Western blot;<br>QuantStudio3 Real-Time PCR System with QuantStudio Design & Analysis Software version 1.5.1 (Thermo Fisher Scientific) and StepOnePlus Real-Time PCR System with StepOne Software version 2.3 (Thermo Fisher Scientific) for qPCR;<br>NextSeq 550 System with NextSeq Control Software version 4.0.0 (Illumina) for RNA-seq and targeted methylation sequencing;<br>Biacore T200 with Biacore T200 software version 3.0 (GE Healthcare) for Surface Plasmon Resonance;<br>GloMax 96 Microplate Luminometer with GloMax 96 Software version 1.9.3 (Promega) and TriStar2 LB 942 Multimode Microplate Reader with ICE software version 1.0.6.0 (Berthold Technologies) for in vitro DNMT activity assay;<br>SpectraMax M3 Multi-Mode Microplate Reader with SoftMax Pro version 7.1 (Molecular Devices) for cell-based nuclear DNMT activity assay;<br>Spectrofluorometer F-2700 with FL Solutions Software version 4.1 (Hitachi High-Tech) for DAN assay;<br>iMark Microplate Reader with Microplate Manager 6 Software version 6.3 (Bio-Rad Laboratories) for the measurement of 5-methylcytosine levels;<br>Epifluorescence microscopy FSX100 with FSX-BSW software version 03.02.12 (Olympus) for cell growth assay;<br>Varian 400-MR with VnmrJ version 3.2 (Varian) for NMR data collection.
Data analysis	Microsoft Excel for Mac version 16.54 was used to calculate pharmacokinetic data and to analyze quantitative data from Western blot, qPCR, spectrometers, fluorometers and luminometers;<br>GraphPad Prism version 8.4.3 (GraphPad Software) was used to generate graphs and perform statistical analysis;<br>Image J version 1.53e (National Institutes of Health) for the quantification of images from Western blot and cell staining;<br>QUMA (http://quma.cdb.riken.jp/top/quma_main_j.html) for processing bisulfite sequencing data;<br>CLC Genomics Workbench version 20.0.4 (Qiagen) for the differential expression analysis of RNA-seq;

MethylSeq version 2.0.0 in BaseSpace Sequence Hub (Illumina) for targeted methylation sequencing;
Integrative Genomics Viewer version 2.8.2 (UC San Diego and the Broad Institute of MIT and Harvard) for the schematic representation of genomic regions of interest;
Proteome Discoverer version version 1.4 (Thermo Fisher Scientific) with MASCOT search engine software version version 2.6.0 (Matrix Science) for LC-MS/MS analysis;
ALICE2 version 6 (JOEL) for NMR data analysis;
ChemDraw version 20.0 (PerkinElmer) for drawing chemical structure;

Following softwares were used for predicting DNMT3B structure;
I-TASSER On-line Server: ZhangGroup, (https://zhanggroup.org/I-TASSER/)
Rosetta version 3.5, (https://www.rosettacommons.org/)
SiteMap version 3.5, Schrödinger Release 2015-3: Schrödinger, LLC, New York, NY. (https://www.schrodinger.com/products/sitemap)

Following softwares were used for virtual screening;
OMEGA version 2.5.1.4: OpenEye, Cadence Molecular Sciences. (https://www.eyesopen.com/omega)
LigPrep version 3.5, Schrödinger Release 2015-3: Schrödinger, LLC, New York, NY. (https://www.schrodinger.com/products/ligprep)
OEDocking version 3.2.0.2: OpenEye, Cadence Molecular Sciences. (https://www.eyesopen.com/oedocking)
Glide version 5.7, Schrödinger Release 2015-3: Schrödinger, LLC, New York, NY. (https://www.schrodinger.com/products/glide)
Maestro version 10.3, Schrödinger Release 2015-3: Schrödinger, LLC, New York, NY. (https://www.schrodinger.com/products/maestro)

Graphics were prepared by;
PyMOL version 1.5.0.5: Schrödinger, LLC, New York, NY. (https://pymol.org)

For manuscripts utilizing custom algorithms or software that are central to the research but not yet described in published literature, software must be made available to editors and reviewers. We strongly encourage code deposition in a community repository (e.g. GitHub). See the Nature Portfolio guidelines for submitting code & software for further information.

Data

Policy information about availability of data

All manuscripts must include a data availability statement. This statement should provide the following information, where applicable:

- Accession codes, unique identifiers, or web links for publicly available datasets
- A description of any restrictions on data availability
- For clinical datasets or third party data, please ensure that the statement adheres to our policy

All data analyzed by targeted DNA methylation sequencing and RNA-seq were deposited in the DNA Data Bank of Japan under the accession number DRA012230 (https://ddbj.nig.ac.jp/resource/sra-submission/DRA012230) and DRA012332 (https://ddbj.nig.ac.jp/resource/sra-submission/DRA012332), respectively.

Human research participants

Policy information about studies involving human research participants and Sex and Gender in Research.

Reporting on sex and gender	The OriGene Tissue Biorepository is comprised of a comprehensive library of over 120,000 high quality human biospecimens representing over 12,000 donor cases. Tissue was collected from US- based large academic medical centers, and samples were acquired only after going through the stringent IRB-approval (Institutional Review Board) process at each medical center. While consent forms were not provided to OriGene, quality assurance mechanisms were instituted to validate compliance with bioethics policies for patient protection.<br>All samples were banked under what is called the "Common Rule" (Federal policy for the protection of human subjects, 45 CFR 46), and data collected were HIPAA compliant. The collections and storage standard operation procedures are compliant with all existing federal, state, local, and institutional requirements. All tissue samples were excised by licensed medical doctors.<br>All materials and associated clinical information are coded and any patient identifiers removed so that no donor can be identified by OriGene or medical researchers. All samples and clinical data were collected and stored within HIPAA guidelines.
Population characteristics	N/A
Recruitment	N/A
Ethics oversight	N/A

Note that full information on the approval of the study protocol must also be provided in the manuscript.

Field-specific reporting

Please select the one below that is the best fit for your research. If you are not sure, read the appropriate sections before making your selection.

☒ Life sciences ☐ Behavioural & social sciences ☐ Ecological, evolutionary & environmental sciences

Life sciences study design

All studies must disclose on these points even when the disclosure is negative.

Sample size	In vitro study, no statistical method was used to predetermine the sample size. The chosen sample size are based on the numbers used for previous publications. For all biochemical and cell biological analyses, qPCR or western blotting experiments or related investigation are repeated at least 3 or 2 times for similar results. In vivo study, no sample size calculations were performed. Sample size of mice experiments was determined based on the previous experiments (ex. Okada F. et al., Br J Cancer, 1992 and Onuma K. et al., Nutrients, 2015).
Data exclusions	In vitro study, data were not excluded from analysis. In vivo study, on principle, data were only excluded for failed experiments, reasons for which included mistakes in the technique and microbial contamination.
Replication	In vitro study, all experiments were repeated independently as indicated in each Figure Legend. All attempts at replication were successful. In vivo study, all experiments and measures were made at least in triplicate (specific replicates are given for each particular experiment) obtaining in all of the cases the similar results. The experimental findings were reliably reproduced.
Randomization	No formal randomization method was used.
Blinding	In vitro study, investigators were not blinded to the sample identities during data collection due to none of the analyses reported involved procedures that could be influenced by investigator bias. In vivo study, mice experiments and statistical analysis were performed by independent researchers in a blinded manner. Mice experiments were performed without any compounds information. All results were analyzed and confirmed by two independent biostatisticians. No blinding was used for in vitro experiments.

Reporting for specific materials, systems and methods

We require information from authors about some types of materials, experimental systems and methods used in many studies. Here, indicate whether each material, system or method listed is relevant to your study. If you are not sure if a list item applies to your research, read the appropriate section before selecting a response.

Materials & experimental systems

n/a	Involved in the study	
☐	☒	Antibodies
☐	☒	Eukaryotic cell lines
☒	☐	Palaeontology and archaeology
☐	☒	Animals and other organisms
☒	☐	Clinical data
☒	☐	Dual use research of concern

Methods

n/a	Involved in the study	
☒	☐	ChIP-seq
☒	☐	Flow cytometry
☒	☐	MRI-based neuroimaging

Antibodies

Antibodies used

Described in "METHODS/Materials": Anti-DNMT3B antiserum was produced by immunization of rabbits with the KLH (keyhole limpet haemocyanin)-conjugated peptide derived from N-terminus of human DNMT3B (MKGDTRHLNGEEDAGGRC) (Sigma Genosys).
The following commercial antibodies were purchased from indicated vendors.
anti-FLAG M2 covalently conjugated to horseradish peroxidase (HRP) (Sigma-Aldrich, clone M2)
anti-NOS2 (Millipore, ABN26)
anti-PTEN (Cell Signaling Technology, 138G6)
anti-GFP (Nacalai Tesque, GF200)
anti-DNMT1 (Cell Signaling Technology, D59A4)
anti-DNMT3A (Sigma-Aldrich, D8695)
anti-rabbit IgG HRP-linked F(ab')2 fragment from donkey (Cytiva, NA9340)
anti-mouse IgG HRP-linked F(ab')2 fragment from sheep (Cytiva, NA9310)
anti-rabbit IgG secondary antibody from goat (LI-COR, 926-32211).

Validation

We specifically decreased endogenous protein by siRNA to validate a prepared anti-human DNMT3B antiserum (Sigma-Genosys).
The other antibodies employed in our study were validated by the manufacturers and used according to the manufacturers' instructions.
anti-FLAG M2 covalently conjugated to horseradish peroxidase (HRP) (Sigma-Aldrich, clone M2)
https://www.sigmaaldrich.com/catalog/product/sigma/a8592?lang=en&region=US
anti-NOS2 (Millipore, ABN26)
https://www.sigmaaldrich.com/catalog/product/mm/abn26?lang=en&region=US
anti-PTEN (Cell Signaling Technology, 138G6)
https://www.cellsignal.com/products/primary-antibodies/pten-138g6-rabbit-mab/9559?site-search-type=Products

anti-GFP (Nacalai Tesque, GF200)
https://www.nacalai.co.jp/global/download/pdf/Epitope_Tag_Antibody.pdf
anti-DNMT1 (Cell Signaling Technology, D59A4)
https://www.cellsignal.com/products/primary-antibodies/dnmt1-d59a4-rabbit-mab/5119?_=1562485658607&Ntt=D59A4&tahead=true
anti-DNMT3A (Sigma-Aldrich, D8695)
https://www.sigmaaldrich.com/catalog/product/sigma/d8695?lang=en&region=US
anti-rabbit IgG HRP-linked F(ab')2 fragment from donkey (Cytiva, NA9340)
https://www.sigmaaldrich.com/US/en/product/sigma/gena93401ml
anti-mouse IgG HRP-linked F(ab')2 fragment from sheep (Cytiva, NA9310)
https://www.sigmaaldrich.com/US/en/product/sigma/gena93101ml
anti-rabbit IgG secondary antibody from goat (LI-COR, 926-32211)
https://www.licor.com/documents/rfm2hw40wf33p06f3ndjrcorwi5usbft

Eukaryotic cell lines

Policy information about cell lines and Sex and Gender in Research

Cell line source(s)	Source of QR-32/QRsP-11 and FPCK-1-1/FPCKpP1-4 cell line were described in "METHODS/Cell culture". The permission of distributors (Drs. N. Kawaguchi and T. Kitagawa, the cancer institute of JFCR) is required for the usage of FPCK-1-1 cell line. Other cell lines (HEK293T, AGS, HeLa and RAW264.7) were purchased from American Type Culture Collection (ATCC).
Authentication	Cell lines used were not authenticated.
Mycoplasma contamination	Cell lines used in this study were routinely tested to be negative for mycoplasma by DAPI staining and PCR analysis.
Commonly misidentified lines (See ICLAC register)	No commonly misidentified cell line was used.

Animals and other research organisms

Policy information about studies involving animals; ARRIVE guidelines recommended for reporting animal research, and Sex and Gender in Research

Laboratory animals	For carcinogenesis experiments, C57BL/6 mice (female, 5 weeks old) obtained from Nippon SLC (Hamamatsu).<br>8-week-old male Crl:CD1 (ICR) mice were used for pharmacokinetic studies.
Wild animals	No wild animals were used.
Reporting on sex	As described in Methods, pharmacokinetic studies were performed using 8-week-old male Crl:CD1 ICR mice. Mouse acute toxicity studies 4-week-old female ICR mice.
Field-collected samples	No field-collected samples ware used in this study.
Ethics oversight	The experimental protocol was approved by the Committee of the Institute for Animal Experimentation of Tottori University (14-Y-14). As described in Methods, animal experiments were conducted in accordance with the animal experimental protocol and guidelines of the Tokyo University of Pharmacy and Life Sciences Animal Experimentation Regulations after review by the Institutional Animal Care and Use Committee (permission numbers L18-10 and L19-26) and approval by the President of the Tokyo University of Pharmacy and Life Sciences.<br>PK studies were performed using male ICR mice in Nemoto Science Co. Ltd. according to the animal experimental protocol and procedures approved by the Institutional Animal Care and Use Committee.

Note that full information on the approval of the study protocol must also be provided in the manuscript.
